# Cross‐scale Material‐Structure Synergy for 2D Metamaterials: Toward Customizable Intelligent Electromagnetic Manipulation in Multiphysics Fields

**DOI:** 10.1002/advs.77742

**Published:** 2026-09-16

**Authors:** Shuhao Wang, Xing Yang, Liping Liu, Yang Peng, Yangyang Wang, Hualiang Lv

**Affiliations:** ^1^ Advanced Laser Technology Laboratory of Anhui Province College of Electronic Engineering National University of Defense Technology Hefei China; ^2^ Institute of Optoelectronics Fudan University Shanghai People's Republic of China

**Keywords:** artificial intelligence, dynamic regulation, EM low observability, inverse design, multispectral compatibility, metasurfaces, stealth technology

## Abstract

Electromagnetic (EM) low‐observable technology is increasingly expected to simultaneously deliver ultrathin geometry, broadband response, dynamic adaptability, and multispectral compatibility ‐capabilities that conventional stealth materials struggle to achieve because of intrinsic constraints such as the Rozanov and Snoek limits and their largely static responses. This review systematically examines recent advances in two‐dimensional metamaterials (metasurfaces) from the perspective of cross‐scale material–structure synergy, with particular emphasis on customizable and intelligent electromagnetic manipulation across multiphysics domains. We first discuss conventional lossy and signature‐controllable materials and identify their performance bottlenecks. We then examine the fundamental mechanisms underlying metasurface‐enabled regulation, including wavefront shaping, localized resonance, and spatial dispersion, and review representative advances in EM, thermal, optical, and acoustic stealth. Particular attention is given to active tuning through electrical, optical, thermal, magnetic, and mechanical stimuli, as well as programmable coding, data‐driven inverse design, artificial intelligence, multispectral compatibility, and multifunctional integration. Finally, key challenges in conformal integration, environmental robustness, scalable fabrication, and coordinated multifunctionality are identified, and future opportunities in embodied intelligence, bionic design, embedded sensing‐control, low‐cost manufacturing, and standardization are discussed toward adaptive, intelligent, and multifunctional low‐observable systems.

## Graphical abstract

Recent advances in metasurface‐enabled low‐observable technologies are reviewed from the perspective of cross‐scale material–structure synergy. Electromagnetic, thermal, optical, and acoustic stealth are highlighted together with dynamic tuning, programmable coding, data‐driven inverse design, artificial intelligence, multispectral compatibility, and multifunctional integration, outlining key challenges and future directions toward adaptive, intelligent, and multifunctional low‐observable systems.

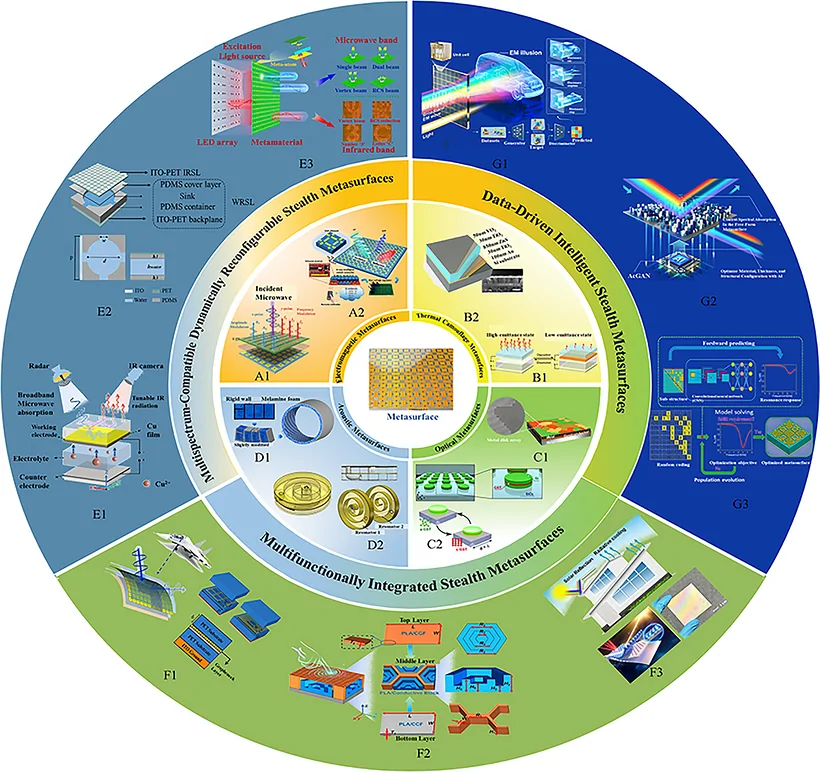

## 1 Introduction

As a fundamental medium for information transmission, energy transfer, and target detection, Controllable EM wave modulation has become a critical pillar of advances in modern electronic information systems, intelligent equipment, EM compatibility, and intelligent sensing [1]. Detection technologies are developing toward multi‐physics integration. Meanwhile, practical scenarios impose higher requirements on intelligent performance and environmental adaptability. Collectively, these trends pose unprecedented challenges to EM low‑observability technologies. Stealth materials must achieve efficient wave absorption or scattering within single spectral bands. They must also enable dynamically tunable EM manipulation under coupled multiphysics conditions (optics, microwaves, acoustics, thermal fields). Such dual capabilities satisfy integrated multifunctional requirements including radar stealth, infrared camouflage, acoustic stealth, and thermal management [2, 3, 4, 5].

Traditional EM low‐observable systems mainly fall into two material categories. The first group refers to lossy media including carbon‐based materials, ferrites, and conductive polymers, which convert incident EM energy into heat via dielectric or magnetic loss mechanisms. Nevertheless, such materials are constrained by inherent physical limitations. The Rozanov criterion indicates that low‐frequency absorption inevitably demands increased material thickness, while the Snoek limit severely impairs the high‐frequency magnetic loss capacity of magnetic substances. Most lossy materials possess fixed operating frequency bands once fabricated, making them incapable of adapting to dynamically variable EM environments [6, 7].

The second category covers signature‐controllable materials such as infrared low‐emissivity coatings, thermochromic materials, and camouflage coatings, which achieve concealment by tuning target thermal radiation and optical reflection signatures. These materials generally suffer from weak multispectral compatibility, slow dynamic response, and limited functionality. For instance, conventional infrared camouflage coatings generally fail to meet low‐scattering requirements in radar bands, and static camouflage schemes fail to match time‐varying environmental backgrounds [8, 9].

These inherent drawbacks prevent conventional materials from fulfilling the comprehensive performance requirements of modern equipment, namely ultrathin and lightweight form factors, dynamic tunability, and multispectral compatibility. The root cause lies in the traditional design paradigm that separates materials and structures, where material properties define functional boundaries and macroscopic structures merely serve as supporting substrates, thereby preventing collaborative subwavelength design and precise programming of multiphysics field responses.

To overcome these inherent limitations, a paradigm shift from single‐scale material optimization to cross‐scale material–structure synergy is essential. The electromagnetic performance of low‐observable systems arises from coupled physical processes across three interdependent design levels. At the molecular level, intrinsic material properties, including electronic band structure, electric and magnetic moments, and charge polarization, determine the fundamental loss mechanisms and tunability. At the microscale, filler morphology, interface quality, defect engineering, and local heterogeneity govern energy‐dissipation efficiency, impedance matching, and localized resonance. At the macroscale, architectural features such as periodic patterns, gradient distributions, multilayer stacks, and three‐dimensional topological frameworks determine the overall propagation, scattering, and wavefront behavior of electromagnetic waves. Optimization confined to a single length scale may therefore improve one aspect of performance while remaining insufficient for practical stealth requirements. The Rozanov and Snoek limits illustrate the trade‐offs that arise when intrinsic material properties and structural constraints are optimized independently. Achieving ultrathin profiles, broadband response, dynamic tunability, and multispectral compatibility consequently requires coordinated design across all three levels.

**FIGURE 1:**
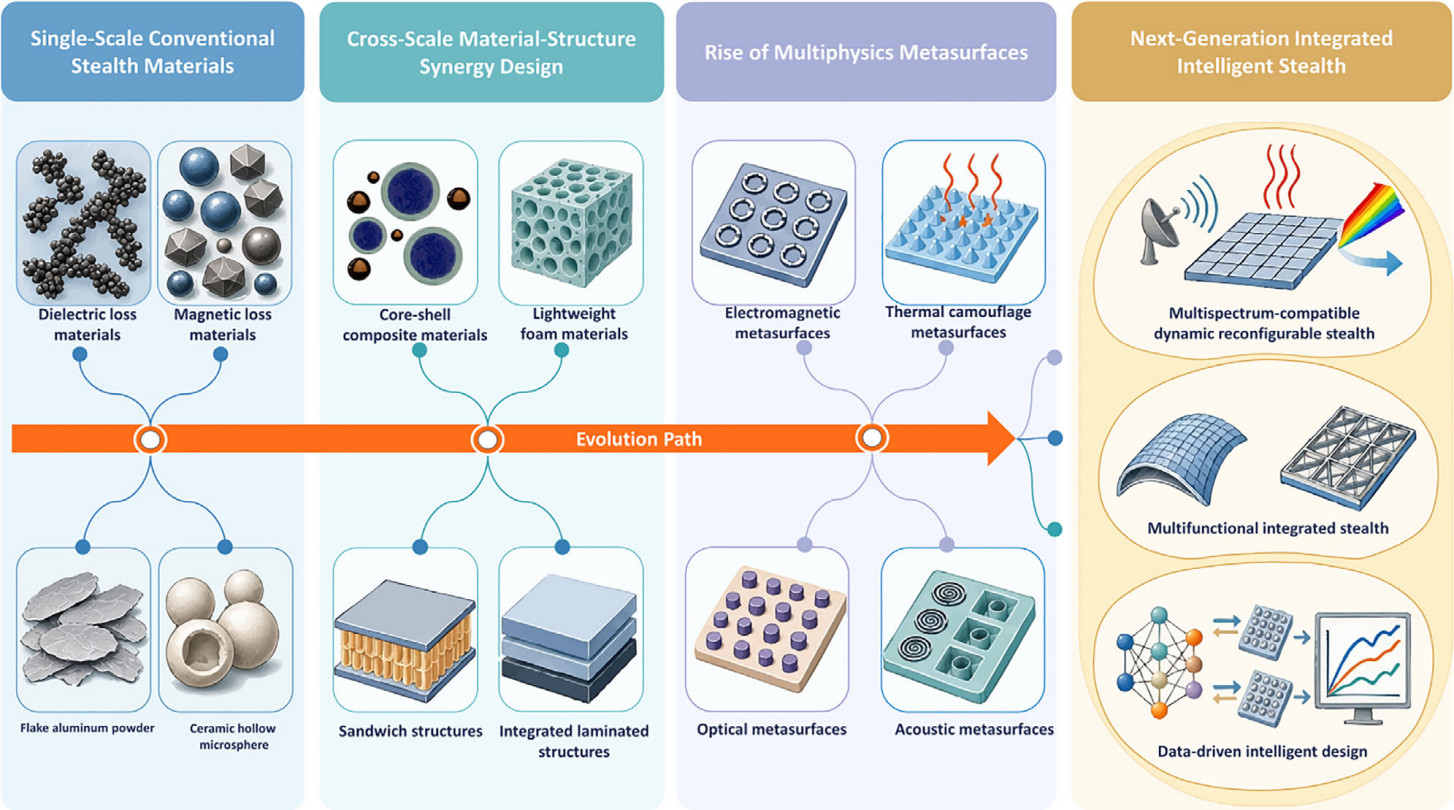
Evolution roadmap of electromagnetic stealth materials and systems.3.

Metamaterials offer an alternative route to address these constraints, with their core advancement lying in a paradigm transition from single material modification to multiscale material‐structure synergistic design [10, 11]. Metasurfaces, a class of two‐dimensional metamaterials constructed from periodically arranged subwavelength meta‐atoms with customized geometries, break the modulation limits of three‐dimensional bulk materials. Through elaborate optimization of meta‐atom geometry, topological architecture, and constituent materials, metasurfaces support active programmable control over EM wave phase, amplitude, and propagation trajectories within subwavelength thicknesses [12, 13]. Their unique superiority stems from three core physical mechanisms. The first is wavefront shaping based on the generalized Snell's law, which introduces continuous inhomogeneous phase gradients to generate anomalous reflection/refraction, beam focusing, and EM vortex beams, eliminating the reliance of conventional optical devices on curved profiles and long optical propagation paths. The second is localized resonance, where meta‐atoms confine EM energy at subwavelength scales through electric dipole, magnetic dipole, or multipole resonances to drastically boost energy dissipation efficiency. The third is spatial dispersion, which broadens the effective modulation bandwidth via collective array responses and overcomes the narrowband constraint of individual resonant units [14, 15, 16]. Compared with traditional bulk materials, metasurfaces feature ultrathin profiles (thinner than one‐tenth of the working wavelength) and lightweight characteristics; by integrating active media (graphene, vanadium dioxide, liquid crystals) or electronic components (PIN diodes, varactor diodes), they can dynamically reconfigure EM responses and open up feasible routes for multispectral‐compatible adaptive modulation [17, 18, 19]. In recent decades, research on stealth metasurfaces has evolved from fundamental mechanism exploration to multi‐scenario practical verification, giving rise to diversified technical branches: EM metasurfaces, thermal camouflage metasurfaces, acoustic metasurfaces, and optical metasurfaces, which realize multispectral modulation across radar, infrared, visible, and acoustic bands [20, 21, 22, 23]. Dynamic tuning strategies have advanced from single‐field stimulation (electrical, optical, and thermal excitation) to multiphysics coupled regulation, and modulation precision has been upgraded from discrete state switching to continuous smooth modulation. Meanwhile, the design paradigm has shifted from trial‐and‐error iteration and pure topological optimization toward data‐driven inverse design. The introduction of intelligent algorithms, including deep learning and generative adversarial networks, has greatly raised the achievable performance upper limit and design efficiency, albeit with inherent limitations in data dependence, interpretability, and fabrication transferability that warrant critical examination [24, 25, 26]. The evolution from conventional stealth materials to intelligent metasurface systems is summarized in Figure 1. This original roadmap highlights four representative stages: single‐scale conventional stealth materials, cross‐scale material–structure synergistic design, multiphysics metasurfaces, and next‐generation integrated intelligent stealth systems. The progression reflects a shift from passive and isolated material responses toward coordinated regulation of EM, thermal, optical, and acoustic signatures through hierarchical architectures, dynamic tuning, multifunctional integration, and data‐driven design.

**FIGURE 2:**
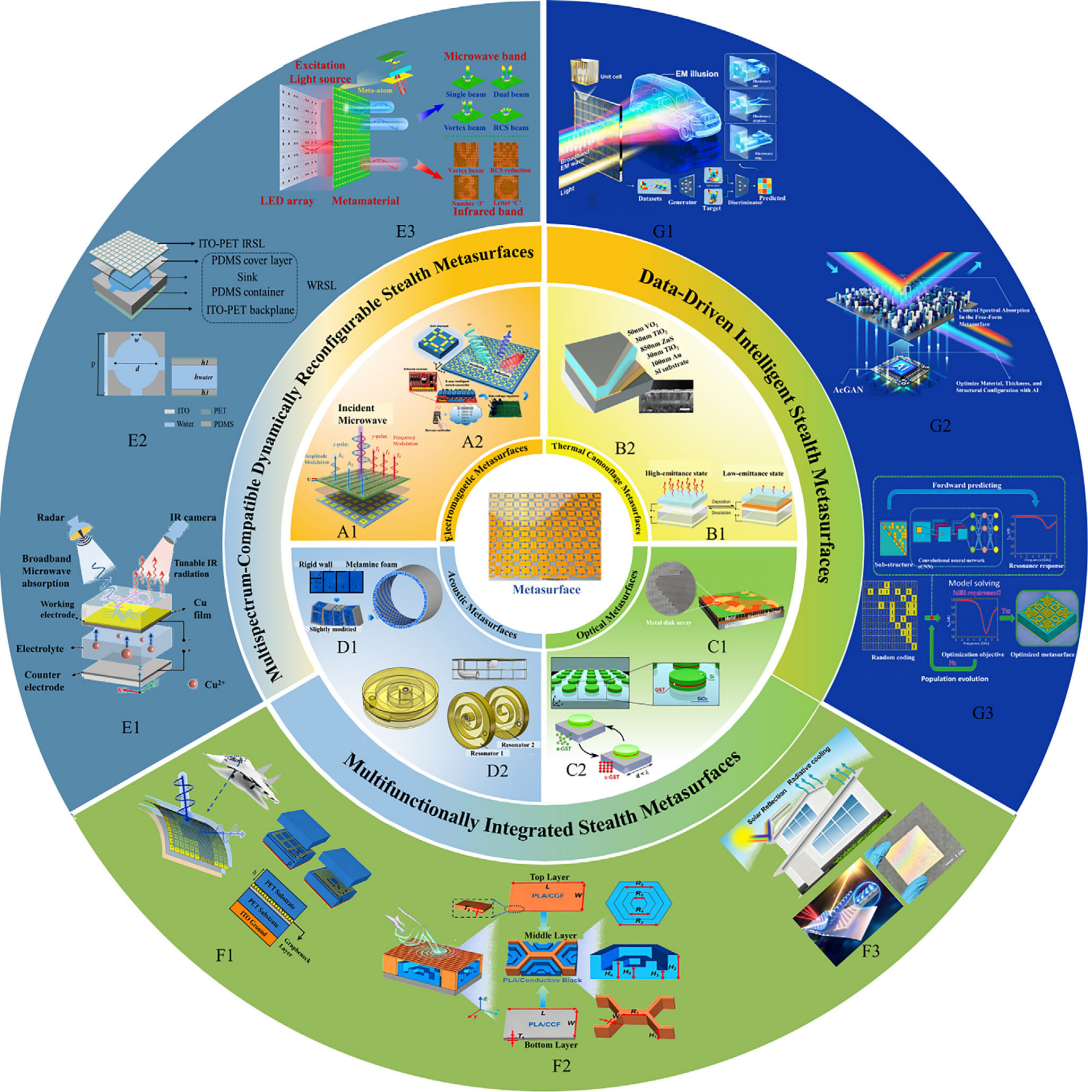
Two‐dimensional metamaterials (metasurfaces) for stealth applications in EM (A1, A2), thermal management (B1, B2), optical (C1, C2) and acoustic (D1, D2) fields, alongside advances in multispectral‐compatible dynamic stealth (E1–E3), multifunctional integration (F1–F3) and data‐driven design (G1–G3). (Reprinted with permission [25, 27, 28, 29, 30, 31, 32, 33, 34, 35, 36, 37, 38, 39, 40, 41, 42]. Copyright 2023 Elsevier Ltd; Copyright 2022 Tsinghua University Press; Copyright 2025 Wiley‐VCH; Copyright 2019 Wiley‐VCH; Copyright 2022 Wiley‐VCH; Copyright 2020 The Optical Society; Copyright 2024 Elsevier Ltd; Copyright 2021 Elsevier Ltd; Copyright 2024 Chinese Laser Press; Copyright 2025 Elsevier Ltd; Copyright 2025 De Gruyter; Copyright 2024 Elsevier Ltd; Copyright 2026 Elsevier Ltd; Copyright 2025 Springer Nature; Copyright 2025 Wiley‐VCH; Copyright 2025 De Gruyter; Copyright 2024 Elsevier Ltd).

Nevertheless, several practical barriers continue to hinder the engineering application of metasurfaces. Large‐area fabrication remains costly, and device performance may deteriorate under harsh operating conditions. The compatibility and coordinated operation of multiple functional modules also remain insufficient. Further advances are therefore required to enable conformal integration with curved platforms, flexible devices, and intelligent robotic systems while ensuring mechanical reliability and long‐term operational stability. Against this background, this review takes metasurfaces as its central focus and systematically examines the evolution of EM stealth materials and technologies. It first analyzes the design strategies and inherent limitations of conventional low‐observable materials. It then introduces the fundamental mechanisms of metasurface‐enabled EM wave manipulation, including the equivalence principle and surface‐impedance model, and clarifies the advantages of metasurfaces over conventional bulk materials. Subsequent sections review recent advances in EM, thermal, optical, and acoustic stealth metasurfaces, with particular emphasis on cross‐scale material–structure synergy and intelligent tuning strategies. Emerging directions, including data‐driven inverse design, multispectral‐compatible dynamic stealth, and multifunctional integration, are subsequently discussed. Finally, the outlook highlights prospective developments in bionic design, embedded intelligent control, scalable fabrication, and standardization toward adaptive and multifunctional stealth systems. Representative developments covered in this review are summarized in Figure 2, including metasurface‐enabled stealth across EM, thermal, optical, and acoustic domains, as well as emerging advances in multispectral dynamic camouflage, multifunctional integration, and data‐driven intelligent design.

## 2 Design Strategies of Traditional EM Wave Low‐Detectability Materials

### 2.1 Lossy Materials

Whether in microwave, laser, or the currently popular terahertz bands, realizing low detectability via EM wave absorption is regarded as a widely used route. EM absorbers refer to functional media that convert incident EM wave energy into alternative forms of energy, such as thermal energy, via intrinsic loss mechanisms for energy dissipation. Two key parameters determine microwave‐absorption performance: impedance matching and EM attenuation capacity. Incident waves hitting absorber surfaces can be divided into reflected, absorbed and transmitted parts. Proper impedance matching between materials and air serves as the core design standard for high‐efficiency absorbers. Materials with matched impedance can trap a larger portion of the incoming EM energy for further dissipation. The characteristic impedance formula reads:

(1) $$Z=\left|\frac{Z_{\mathrm{in}}}{Z_{0}}\right|=\sqrt{\frac{\mu_{r}}{\varepsilon_{r}}}\;\left|\tanh\left(j\frac{2\pi \mathrm{fd}}{c}\sqrt{\mu_{r}\varepsilon_{r}}\right)\right|$$

In this formula, *Z_in_* means input impedance and *Z*_0_ stands for free‐space impedance. Other parameters include frequency *f*, composite thickness *d*, light speed *d*, complex permeability μ_*r*_ and complex permittivity ε_*r*_. Reflection loss below −10 dB corresponds to effective absorption bandwidth (EAB), where more than 90% of incident wave energy is absorbed inside the material.

Based on the impedance matching principle, EM energy dissipation mechanisms fall into two categories: dielectric loss and magnetic loss. Complex permittivity and complex permeability are core parameters governing the ultimate microwave absorption (MA) performance. The real parts (ε_*r*_^′^, μ_*r*_^′^) characterize EM energy storage capability, while the imaginary parts (ε_*r*_^′′^, μ_*r*_^′′^) reflect EM energy loss capability [43].

(2) $$\begin{array}{c} \varepsilon_{r}=\varepsilon^{\prime }-j\varepsilon^{\prime \prime }\\ \mu_{r}=\mu^{\prime }-j\mu^{\prime \prime }\\ \tan\delta_{\varepsilon }=\varepsilon^{\prime \prime }/\varepsilon^{\prime }\\ \tan\delta_{\mu }=\mu^{\prime \prime }/\mu^{\prime } \end{array}$$

Dielectric loss describes the EM energy dissipation induced in media under EM irradiation. The imaginary component ε_*r*_^′^′ of the relative permittivity ε_*r*_ quantifies dielectric dissipation during wave‐matter interaction, which is commonly characterized by the dielectric loss tangent *tan*δ_*e*_. Magnetic loss is another key metric for evaluating EM absorbers, arising when materials are subjected to an alternating magnetic field. During magnetization processes (e.g., hysteresis, eddy currents, and domain‐wall resonance), magnetic media irreversibly convert a portion of incident EM energy into heat. The magnetic loss tangent *tan*δ_*m*_ and complex permeability serve as the dominant indicators for quantifying the magnitude of magnetic loss in absorbing materials.

#### 2.1.1 Dielectric Loss Materials

##### 2.1.1.1 Carbon‐Based Materials

**FIGURE 3:**
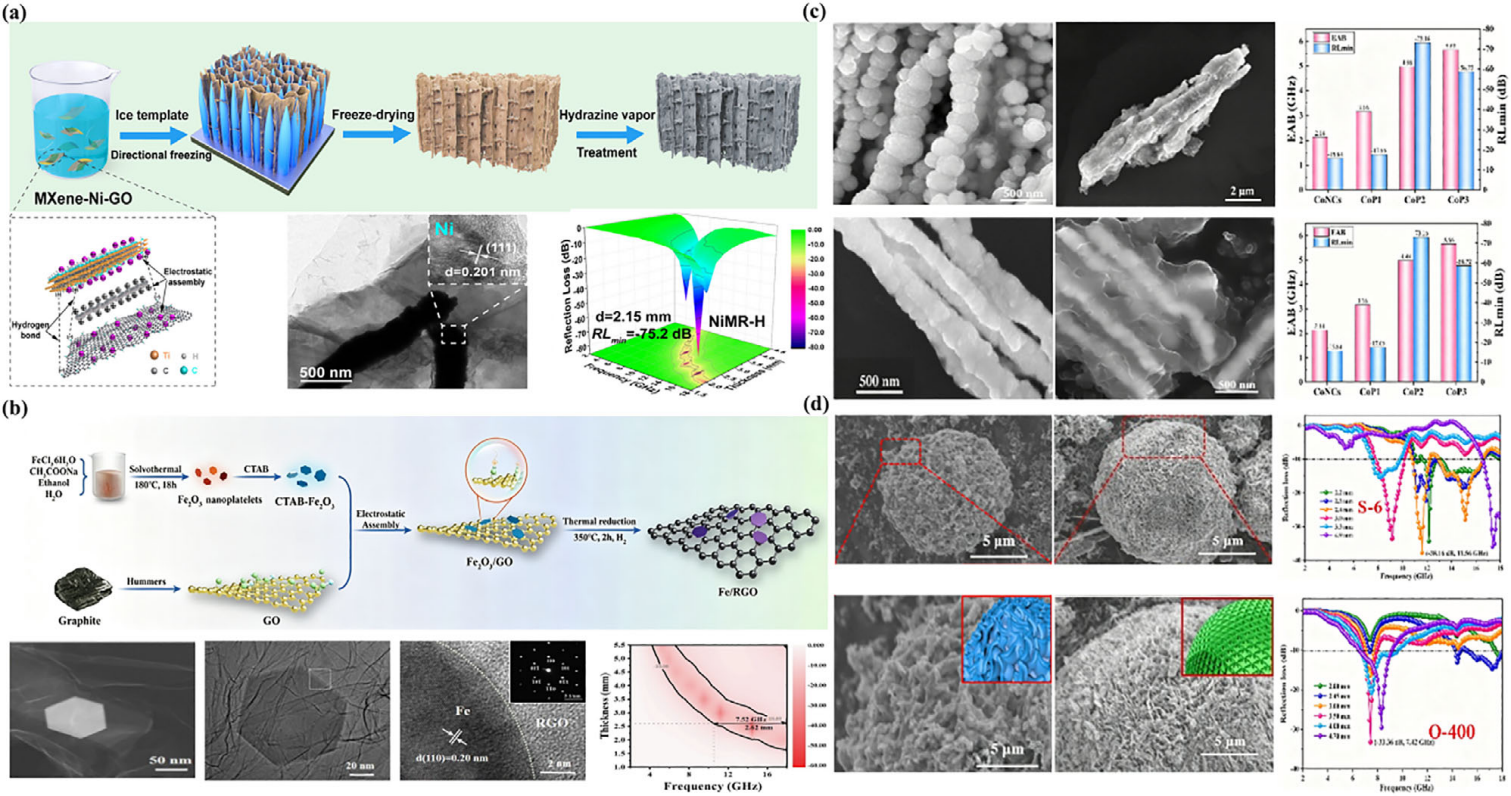
Dielectric loss materials. (a) Multifunctional Magnetic Ti_3_C_2_T_x_ MXene/Graphene Aerogel. Reprinted with permission [45]. Copyright 2021 American Chemical Society. (b) Trace Fe‐decorated graphene: Interfacial carrier injection enables efficient broadband absorption. Reprinted under the terms and conditions of the Creative Commons Attribution (CC BY) license (https://creativecommons.org/licenses/by/4.0/) [46]. Copyright 2024 The Authors, published by Springer Nature. (c) One‐dimensional M(Co, Ni)@polyaniline core‐shell nanochains: Magnetic‐dielectric synergistic absorption with tunable thickness and ultrahigh reflection loss. Reprinted with permission [47]. Copyright 2022 Elsevier. (d) Sulfuration‐induced vacancy/interface engineering of Co(OH)F/Zn_0.76_Co_0.24_S/Co_3_S_4_ composites for boosted microwave absorption. Reprinted with permission [48]. Copyright 2021 Elsevier.

Carbon‐based materials are widely adopted as EM absorbers due to their outstanding dielectric properties, lightweight feature, and superior chemical stability. The material system has evolved from conventional carbon black to carbon fibers, carbon nanotubes (CNTs), graphene, MXene, and their multifunctional composites. CNTs possess unique one‐dimensional tubular architectures, large specific surface areas, favorable electrical conductivity, and tunable microstructures, making them ideal candidates for high‐efficiency dielectric loss absorbers. Zhang et al. [44] fabricated Co@CNT/porous carbon composites via MOF‐derived pyrolysis. Benefiting from the synergistic coupling of Co nanoparticles, CNTs, and porous carbon substrates, the composite achieves a minimum reflection loss (RL) of −56.23 dB at a matching thickness of 2.3 mm, together with a maximum effective EAB of 7.36 GHz. MXene, a family of two‐dimensional (2D) transition metal carbides/nitrides first reported in 2011, exhibits tremendous potential for EM absorption by virtue of its layered stacking structure, high electrical conductivity, large specific surface area, and abundant surface functional groups. High intrinsic conductivity easily leads to severe impedance mismatch, which can be effectively mitigated via rational structural design (e.g., constructing three‐dimensional porous aerogel frameworks). For instance, Liang et al. [45] synthesized magnetic Ni nanochain‐embedded Ti_3_C_2_T_x_ MXene/reduced graphene oxide (RGO) aerogels through directional freezing and hydrazine vapor reduction, as illustrated in Figure 3a. The as‐prepared Ni/MXene/RGO (NiMR‐H) aerogel features an ultra‐low density of only 6.45 mg/cm^3^ and delivers EM absorption (EMA) performance that represents the state of the art among all reported MXene‐based absorbers, with a RL_min_ of −75.2 dB and a broad EAB covering 7.3 GHz. Graphene possesses interconnected porous networks, large specific surface areas, and exceptional conductivity, so it has great potential for radar‐absorbing materials. Zhang et al. [46] revealed that the excellent wave‐absorbing capacity of Fe/RGO composites originates from carrier injection induced by Fe nanosheets anchored on graphene substrates, which strengthens both polarization loss and conductive loss, as depicted in Figure 3b. At an ultralow filler loading of merely 2 wt%, the composite realizes a RL_min_ of −53.38 dB at a thickness of 2.45 mm, and an EAB of 7.52 GHz at 2.62 mm. At high filler loadings, carbon materials tend to exhibit excessive conductivity. This creates poor impedance matching and generates intense surface reflection instead of energy absorption. Their absorption peaks tend to be narrow and shift as filler content changes. Such materials are suitable for lightweight thin‐film coatings, but may not work well for wideband or low‐frequency stealth demands.

##### 2.1.1.2 Conducting Polymers

Intrinsically conducting polymers (ICPs) are attractive candidates for EM absorbers on account of their tunable electrical properties, low mass density, designable microstructures, and outstanding corrosion resistance. Nevertheless, pristine ICPs suffer weak EM energy dissipation capacity, which restricts their standalone application in EMA. To address this limitation, ICPs are usually coated or encapsulated with other functional substances to construct heterogeneous architectures rich in dielectric‐magnetic multi‐interfaces. As reported by Dai et al. [47] one‐dimensional M(Co, Ni)@polyaniline (PANI) core‐shell nanochains were synthesized via in situ polymerization under a parallel magnetic field, as presented in Figure 3c. This structural design takes full advantage of the synergistic effect between PANI's dielectric loss (dipole and interfacial polarization) and the magnetic loss of Co/Ni nanochains (natural resonance and exchange resonance). Notably, the one‐dimensional nanochain can act like an antenna to enhance EM wave capture efficiency, while abundant core‐shell heterogeneous interfaces greatly accelerate interfacial polarization relaxation. Such composite design overcomes the performance bottlenecks of single‐component materials. At a filler loading of 30 wt%, the material realizes strong wave attenuation with RL_min_ as low as −73.16 dB and an ultra‐broad EAB up to 5.88 GHz, which verifies the irreplaceable status of ICPs in fabricating high‐performance EMA composites. Conductive polymers provide tunable conductivity via doping/dedoping and good corrosion resistance, but their thermal stability is limited, and achieving uniform doping over large areas remains challenging. They are suitable for flexible or smart coatings where environmental adaptability is needed, but not for high‐temperature or high‐power scenarios.

##### 2.1.1.3 Transition Metal Dichalcogenides

Transition metal dichalcogenides (TMDs) represent an emerging family of promising EM absorbers, benefiting from their tunable metallic components, abundant crystal morphologies, controllable defect densities, and excellent dielectric characteristics. Among multiple optimization strategies for TMD‐based absorbers, defect engineering is an effective strategy, and the introduction of sulfur (S) vacancies is the most widely investigated route. S vacancies not only serve as localized charge centers to trigger intense dipole polarization, but also balance conductive loss and impedance matching by regulating carrier concentrations; simultaneously, they introduce extra defect energy levels within the bandgap to broaden the effective absorption frequency range. Liu et al. [48] introduced high‐density S vacancies, lattice distortions, and heterogeneous interfaces into Co(OH)F/Zn_0_._76_Co_0_._24_S/Co_3_S_4_ composites via controlled sulfidation, as shown in Figure 3d. The synergistic coupling of these structural defects and multi‐heterointerfaces remarkably reinforces interfacial polarization and conductive loss. The optimized Zn‐Co sulfide sample (S‐6) achieves an ultra‐broad EAB of 5.85 GHz at a thickness of 2.2 mm, along with a RL_min_ of −38.16 dB at 2.4 mm, which significantly outperforms its oxide counterparts. Despite great progress in TMD‐based EM absorbers, several critical challenges remain unsolved: complicated synthetic processes with difficult precise structural control, inferior environmental and thermal stability that shortens service life, and excessive dependence on microscale defects that causes nonlinear degradation of wave‐absorbing performance.

#### 2.1.2 Magnetic Loss Materials

In recent years, magnetic loss materials, including magnetic metal nanoparticles, their alloys, and oxides, have attracted extensive research interest. Benefiting from unique magnetic properties and mature synthetic techniques, these materials occupy a vital position in the field of EM wave absorption.

##### 2.1.2.1 Fe/Co/Ni and Their Alloys

**FIGURE 4:**
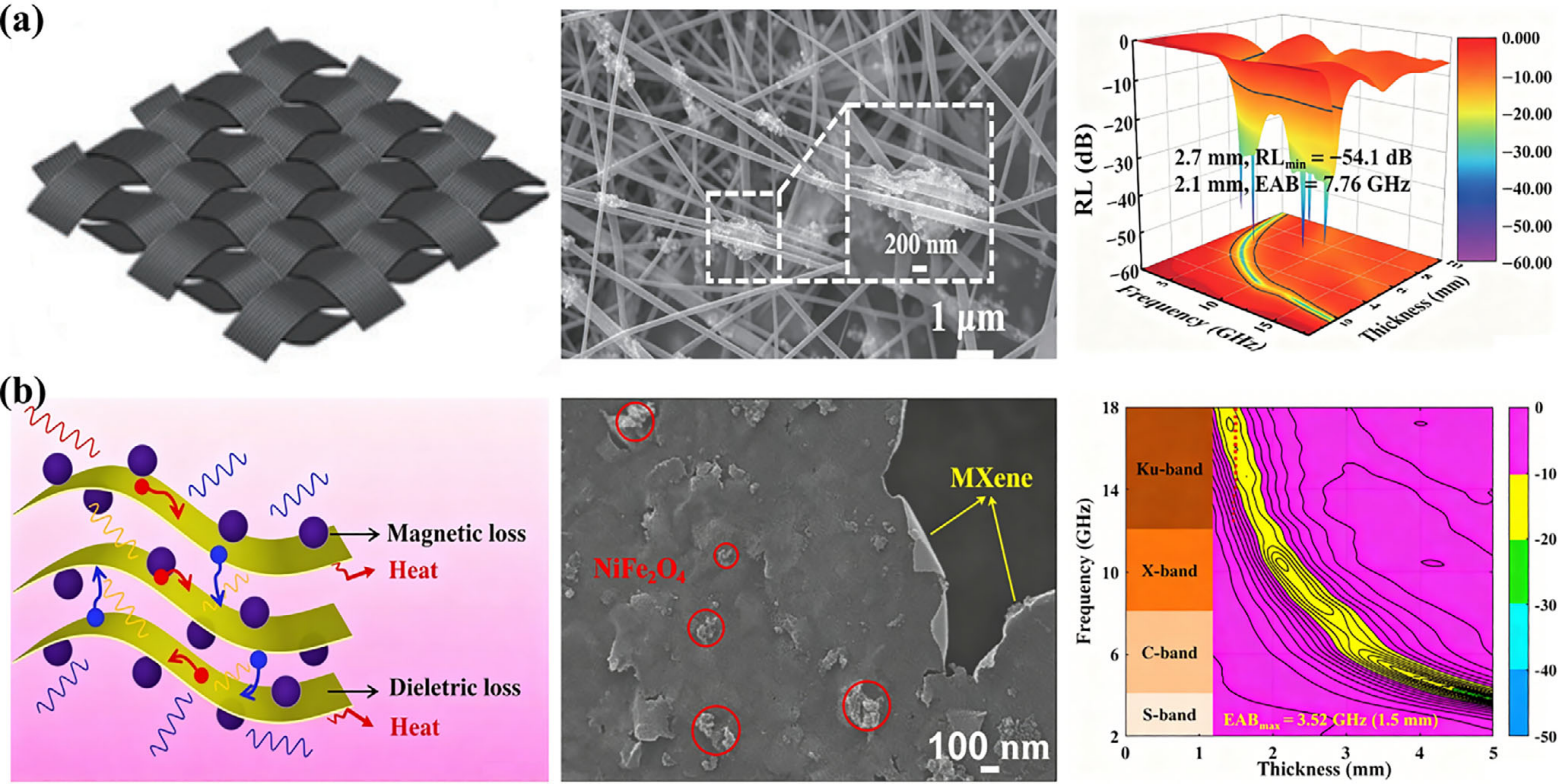
Magnetic loss materials. (a) Flexible, waterproof Fe_0.64_Ni_0.36_/MXene/CNFs nanofibrous membranes with multicomponent heterostructures for efficient EM wave absorption. Reprinted with permission [50]. Copyright 2022 Tsinghua University Press. (b) Electrostatic self‐assembled magnetic NiFe_2_O_4_/Ti_3_C_2_T_x_ MXene nanocomposites. Reprinted with permission [52]. Copyright 2021 Springer Nature.

Magnetic metal powders generally feature high saturation magnetization and permeability, which makes them classic EM absorbers. Current research mainly optimizes EMA performance by regulating particle size, surface morphology, and elemental composition. Yu et al. [49] fabricated three‐dimensional dendritic α‐Fe microstructures via electric‐field‐induced electrochemical reduction. The intricate branched polycrystalline architecture significantly boosts magnetic anisotropy and coercive force. The prepared material achieves a reflection loss (RL) of −32.3 dB at a thickness of 1.9 mm, while its EAB nearly covers 12 GHz at 1.5 mm. This study verifies that dimensional and morphological co‐design can synergistically optimize magnetic loss and impedance matching, providing a feasible strategy for lightweight, broadband metal‐based absorbers. Nevertheless, magnetic metals exhibit strong inherent chemical reactivity and are prone to oxidation, leading to poor environmental stability and restricting their direct practical application. To overcome this drawback, composite design is widely adopted by combining magnetic metals with dielectric media. Such composite structures can form protective encapsulation to resist corrosion and improve chemical durability; meanwhile, tuning EM parameters and activating multi‐loss synergies optimizes impedance matching, thus achieving stronger and wider EM wave attenuation. Following this design idea, Zhang et al. [50] fabricated flexible Fe_0.64_Ni_0.36_/MXene/carbon nanofiber (CNFs) composite membranes via electrospinning, as illustrated in Figure 4a. This composite constructs a multi‐dimensional heterogeneous system composed of zero‐dimensional magnetic alloy nanoparticles, two‐dimensional MXene nanosheets, and one‐dimensional carbon nanofibers. During high‐temperature carbonization, the NiFe_2_O_4_ precursor nanoparticles are reduced to Fe_0.64_Ni_0.36_ magnetic alloy grains, which are uniformly embedded and anchored within the continuous conductive network formed by carbon nanofibers and MXene. The carbon/MXene protective network effectively isolates magnetic alloys from ambient oxidation and greatly enhances structural stability. The composite delivers outstanding comprehensive EMA performance. At an ultralow filler loading of 12.5 wt%, a RL_min_ of −54.1 dB is obtained at 2.7 mm, alongside an ultra‐broad EAB of 7.76 GHz at a thin matching thickness of 2.1 mm. Fe/Co/Ni and their alloys provide high saturation magnetization and strong magnetic loss in the low‐to‐medium frequency range, but they are heavy, prone to oxidation, and exhibit eddy current losses at high frequencies, which reduces permeability. They are ideal for low‐frequency, high‐absorption applications (e.g., anechoic chambers) but not for weight‐sensitive or high‐frequency platforms.

##### 2.1.2.2 Magnetic Metal Oxides

Magnetic metal oxides, especially ferrites, have long been regarded as mainstream EM absorption and shielding materials on account of their excellent magnetic behavior, high electrical resistivity, favorable chemical stability, and low cost. However, skin effect and high bulk density are two core bottlenecks that limit the high‐efficiency EMA capacity of conventional ferrites. Constructing hollow/porous microstructures or compounding with lightweight substrates is an effective approach to reduce overall density. Qin et al. [51] reported a lightweight NiO/NiFe_2_O_4_/Ni nickel foam composite. Porous nickel foam acts as a lightweight supporting scaffold, while in situ grown NiO/NiFe_2_O_4_ dielectric layers anchor on the metal framework to realize material‐structure synergism. The foam skeleton lowers material density and generates abundant gas‐solid interfaces to optimize impedance matching and facilitate inward EM wave propagation. Simultaneously, in situ formed oxide heterojunctions trigger strong interfacial and defect polarization, which greatly strengthens dielectric loss. Impressively, the composite realizes an ultra‐broad EAB of 14.24 GHz at an ultrathin thickness of only 0.6 mm, with a normalized effective absorption bandwidth up to 19444.4 GHz·g^−1^·cm^2^. This result demonstrates that integrating magnetic metal oxides with three‐dimensional lightweight metal foams can simultaneously solve the problems of high density, mismatched impedance, and insufficient attenuation capacity, offering new guidance for designing next‐generation lightweight broadband absorbers. In addition, hybridization with high‐permittivity substances can further optimize impedance matching. MXene‐ferrite nanocomposites have become promising EMA candidates in recent years. Guo et al. [52] fabricated NiFe_2_O_4_/MXene composites via electrostatic self‐assembly, as shown in Figure 4b. Positively charged PDDA‐modified NiFe_2_O_4_ nanoparticles electrostatically bind to negatively charged MXene nanosheets, which suppresses MXene restacking and generates abundant heterogeneous interfaces. The composite exhibits excellent EMA performance at an ultralow filler loading of 3 wt%, with RL_min_ reaching −41.83 dB. When the filler content increases to 5 wt% loading, the EAB covers a wide frequency range of 3.44–18 GHz. Ferrites offer good chemical stability, low eddy current loss, and low cost, but their saturation magnetization is lower than metallic magnets, and their high‐frequency performance is severely limited by the Snoek limit. Hexaferrites (e.g., BaFe12O19) extend the operating frequency into the millimeter‐wave range due to their strong anisotropy, yet they are brittle and difficult to process. Ferrites are suitable for moderate‐frequency, cost‐sensitive applications, while hexaferrites are preferred for millimeter‐wave stealth.

#### 2.1.3 Material‐Structure Synergistic Design

This subsection exemplifies the cross‐scale material‐structure synergy at the microscale and macroscale: the atomic/molecular composition determines intrinsic loss mechanisms, the microscale core‐shell or laminate architecture optimizes impedance matching and interfacial polarization, and the macroscale assembly (foam, sandwich) shapes the global EM response. The following cases illustrate how coordinated design across these scales breaks the performance limits of single‐scale approaches.

##### 2.1.3.1 Magnetoelectric Synergistic Wave‐Absorbing Core‐Shell Structures

Traditional single‐component absorbers such as ferrites feature strong magnetic loss but suffer from low permittivity and high bulk density. At high frequencies, skin effect and eddy current loss easily trigger impedance mismatch, severely restricting their application in broadband, lightweight absorbing devices. Carbonaceous materials including graphene and carbon nanotubes possess outstanding electrical conductivity and low weight, which endows them with strong dielectric‐loss capability. Nevertheless, relying solely on dielectric loss cannot realize broadband high absorption; excessively high conductivity also readily leads to impedance mismatch. Against this background, hybridizing materials with complementary loss mechanisms—especially the rational design of microscale composite architectures—has become a core strategy to break the inherent performance limitations of single‐component absorbers and boost EMA capacity. Core–shell nanocomposites have attracted extensive research interest by virtue of their tunable magnetoelectric synergies and unique structural advantages. In such architectures, the core and shell layers can be independently engineered with distinct EM functional media. Typically, a magnetic core encapsulated by a dielectric shell achieves coupled magnetic and dielectric loss, and strengthens wave attenuation via interfacial polarization. Meanwhile, the outer shell serves as a protective layer to improve structural stability. Precise tuning of core–shell composition, particle size and morphology enables flexible regulation of EM parameters, thus optimizing impedance matching and EM wave attenuation capability.

**FIGURE 5:**
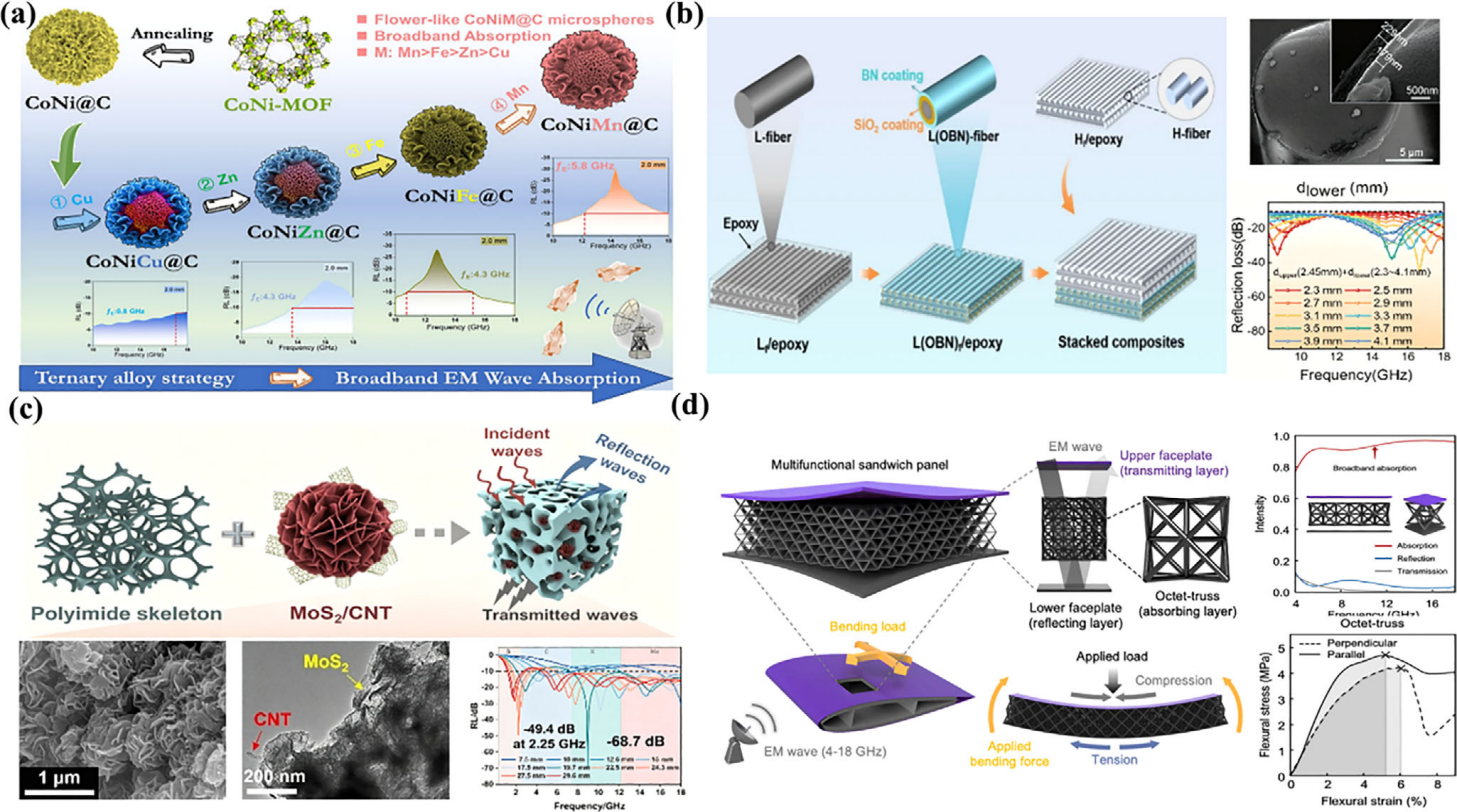
Structure‐material collaborative design. (a) MOF‐derived flower‐like CoNiMn@C microspheres with magnetic alloy@carbon core‐shell architecture. Reprinted under the terms and conditions of the Creative Commons Attribution (CC BY) license (https://creativecommons.org/licenses/by/4.0/) [53]. Copyright 2024 The Authors, published by Springer Nature. (b) Gradient stacking of SiO_2_/BN dual‐coated low‐resistivity SiC fibers and high‐resistivity SiC fibers. Reprinted with permission [57]. Copyright 2025 Science China Press. (c) Lightweight 3D interconnected porous carbon foam reinforced with tunable 1T/2H‐MoS_2_/CNT heterostructures achieves exceptional low‐frequency EMWA. Reprinted with permission [58]. Copyright 2025 Elsevier. (d) Multifunctional seamless meta‐sandwich composite as lightweight, load‐bearing, and broadband‐EM‐wave‐absorbing structure. Reprinted with permission [60]. Copyright 2024 Elsevier.

Constructing magnetic core/carbon shell composites is an effective strategy to enhance EMA performance, and such superiority originates from unique microstructural features. On the one hand, abundant heterogeneous interfaces between magnetic cores and carbon shells efficiently induce interfacial polarization. On the other hand, intrinsic lattice defects inside carbon matrices act as active dipole polarization centers. The synergistic effect of the two loss mechanisms significantly reinforces dielectric loss and optimizes overall wave‐absorbing performance. For example, Che et al. [53] synthesized flower‐like micro‐nano magnetic‐carbon CoNiM@C (M = Cu, Zn, Fe, Mn) microspheres with tunable EM responses, as illustrated in Figure 5a. Introducing ternary alloy elements further optimizes broadband absorption performance. In terms of EAB, the performance follows the order Mn > Fe = Zn > Cu. Among all systems, CoNiMn@C microspheres deliver an EAB of 5.8 GHz.

Coating magnetic cores with secondary semiconductor shells represents another efficient approach to strengthen energy dissipation, broaden absorption bandwidth, and improve overall EMA performance. Typical semiconductor materials including ZnO, MnO_2_, MoS_2_ and TiO_2_ are ideal shell candidates owing to facile synthesis, excellent chemical durability and tunable dielectric loss. The semiconductor shell not only tailors the overall permittivity of core–shell composites to balance the magnetic core and dielectric coating, but also endows absorbers with superior thermal and corrosion resistance, which is critical for long‐term service in harsh environments. Shi et al. [54] fabricated Fe_3_O_4_@black TiO2‐x core–shell heterostructures via vacuum surface modification. The disordered TiO2‐x coating tightly wraps Fe_3_O_4_@TiO_2_ to form multi‐interfacial architectures, where defect dipoles are confined within the black titania layer. The improved wave absorption mainly arises from two factors: multi‐interface coupling and defect dipoles jointly boost polarization loss; hierarchical TiO2–TiO2‐x layers facilitate magnetic field penetration from the Fe_3_O_4_ core and realize efficient magnetoelectric collaborative attenuation. The as‐prepared heterostructure achieves an EAB of 13.0 GHz and a minimum reflection loss RL_min_ of −47.6 dB.

Furthermore, polymer shells are promising candidates for high‐performance absorbers due to low density, easy processing, stable chemical properties and abundant surface functional groups. Polymer coatings can act as insulating layers to optimize impedance matching. In particular, conductive polymer shells such as PANI, PPy and PTh introduce extra conductive loss and generate synergistic effects with core magnetic/dielectric loss, further elevating total wave attenuation capacity. Chen et al. [55] fabricated BCNF/PANI/PVA composite aerogels for EM shielding of integrated control cabinets. At a matching thickness of 6.11 mm, the composite reaches an optimal RLmin of −53.19 dB at 4.16 GHz with an EAB of 2.20 GHz, demonstrating outstanding low‐frequency absorption capability. In addition, polymer shells improve the dispersion uniformity of core fillers inside polymer matrices and enhance composite mechanical and machinable properties. Feng et al. [56] synthesized Ni/NiO/C/MnO_2_@PPy composites via hydrothermal treatment, high‐temperature calcination and in situ polymerization. The optimized NCMP‐40 sample exhibits −56.23 dB at 3.87 mm and an EAB of 6.86 GHz at 1.97 mm, fully covering the full X‐band and Ku‐band range. Radar cross section (RCS) simulations were carried out to evaluate scattering suppression. When coated on perfect electric conductor (PEC) substrates, the NCMP‐40 composite maintains RCS values consistently below −10 dBm^2^ for −60° < θ < 60°, with a minimum RCS of −22.0 dBm^2^ at normal incidence (θ = 0°).

Although these core‐shell structures achieve enhanced absorption via magnetic‐dielectric coupling, their fabrication requires precise control of shell thickness and uniformity, limiting scalability. They are best suited for thin‐film or coating applications where weight is less critical.

##### 2.1.3.2 Integrated Laminated Plates

Rational material integration and layered structural design of laminated plates enable broadband EM absorption within limited thickness. Such laminates consist of stacked multilayer functional films with customized permittivity, permeability and conductivity. The synergistic interaction between adjacent layers optimizes overall impedance matching and wave attenuation, simultaneously realizing ultrathin geometry and wide absorption bandwidth. Li et al. [57] proposed an innovative strategy combining fiber coating and laminated stacking. Single‐layer BN and bilayer SiO_2_/BN coatings were deposited on low‐resistivity SiC fibers (L‐fibers), which were further stacked with high‐resistivity SiC fibers (H‐fibers) to construct gradient‐impedance architectures. The as‐fabricated SiC fiber/epoxy composites feature core–shell interfaces and macroscopic gradient impedance, realizing full‐band absorption across X and Ku bands, as shown in Figure 5b. This work verifies the synergistic mechanism of fiber coating and lamination: surface coatings introduce abundant multiple‐scattering sites and interfacial polarization to strengthen EM attenuation, while stacked gradient structures optimize global impedance matching. This design provides new guidelines for lightweight broadband EMA composites. Nevertheless, realizing strong absorption and ultra‐wide bandwidth under ultrathin thickness remains a major challenge. Especially for low‐frequency EM waves with long wavelengths, thick materials or complex microstructures are required for effective absorption, which conflicts with the lightweight, ultrathin requirements of modern devices.

##### 2.1.3.3 Lightweight Foam Materials

Foam‐based absorbing materials represent another important category of structurally designed EMA composites. Wave‐absorbing functional fillers can be embedded inside foam matrices, or foam skeletons themselves are designed as loss units, combining ultra‐low density with excellent absorption performance. However, conventional foams suffer from poor mechanical load‐bearing capacity and weak high‐temperature resistance, restricting their practical application. To overcome these drawbacks, Zhou et al. [58] replaced conventional foam substrates and adopted high‐performance polyimide as a precursor. Flower‐like MoS_2_/CNT absorbers with tunable 1T‐phase content were synthesized via hydrothermal methods and uniformly dispersed in polyimide solutions. A thermally induced phase separation foaming technique was adopted to expand precursor particles into hollow microspheres; gas compression concentrates MoS_2_/CNT on pore walls. Subsequent thermal cyclization and carbonization at 650°C convert polyimide skeletons into carbon frameworks, producing MoS_2_/CNT‐reinforced carbon foam (MC@CF), as illustrated in Figure 5c. This multifunctional foam delivers prominent low‐frequency absorption performance, with RL_min_ reaching −49.4 dB (S band) and −68.7 dB (C band), and a maximum EAB of 14.9 GHz. Meanwhile, the composite possesses improved mechanical strength, outstanding thermal stability and thermal insulation, offering a feasible lightweight, tough absorber solution for extreme service environments. Although lightweight foam materials provide excellent impedance matching and low density, their mechanical strength is poor and absorption bandwidth is often narrow unless combined with magnetic fillers. They are preferred for weight‐sensitive platforms (e.g., UAVs) but require protective encapsulation.

##### 2.1.3.4 Lightweight and Broadband Integrated Sandwich Structures

Lightweight load‐bearing sandwich structures have been widely adopted in aerospace applications. They consist of two thin high‐strength face sheets and a thick low‐density core layer, forming standard sandwich configurations. The core region can be designed with honeycomb, foam and other topological geometries. Traditional honeycomb structures require extra reinforcement and encapsulation, limiting their adaptability to complex service conditions. Therefore, high‐performance sandwich architectures and carbon fiber composite layouts have been extensively explored. For instance, Du et al. [59] fabricated multiscale carbon fiber/epoxy (CFC/GCA/EP) composites by inserting graphene oxide/carboxymethyl cellulose aerogel (GCA) between carbon fiber cloth layers to construct 3D network sandwich structures. The preparation process includes electrochemical oxidation, GO grafting and directional freeze‐drying to form ordered porous networks. Structural characterization demonstrates that the 3D GCA network extends EM propagation paths and provides abundant interfacial polarization sites while optimizing free‐space impedance matching. The composite achieves an EAB of 8.92 GHz and RL_min_ = −61.7 dB at a thickness of 2 mm. Mechanically, graphene oxide nanosheets enhance fiber‐resin interfacial bonding, deflect crack growth and disperse internal stress. The mode I and mode II critical strain energy release rates rise by 11.16% and 32.56%, respectively, while peak impact force increases by 21.36%, fully demonstrating the advantages of structure‐function integration. Lim et al. [60] developed seamless multifunctional metallic sandwich composites based on octet‐truss lattices, as depicted in Figure 5d. The structure is integrally manufactured via multi‐material 3D printing, consisting of a wave‐transparent polylactic acid top sheet, carbon black‐filled octet‐truss EMA core, and carbon black‐reinforced reflective bottom sheet. EM simulation results show that the single‐layer core with a thickness of only 10 mm achieves an average EM absorption efficiency of 95% within 4–18 GHz. In addition, the sandwich composite exhibits excellent mechanical performance with a flexural strength of 4.70 MPa. Its isotropic load‐bearing capacity eliminates mechanical anisotropy caused by printing direction or external loading, demonstrating robust structural reliability. Lightweight/broadband integrated sandwich structures achieve both low weight and wide bandwidth by combining a lossy core with impedance‐matching skins, but their performance is sensitive to core thickness and skin conductivity. They are promising for large‐area conformal applications but demand careful optimization of the core‐skin interface.

### 2.2 Signature‐Controllable Low Detectability Technology

Beyond the loss‐type absorbing mechanisms discussed above, another important stealth paradigm relies on manipulating target signature signals rather than dissipating incident energy. Signature‐controllable stealth materials operate without relying on external EM wave energy dissipation. Instead, they modulate intrinsic target characteristic signals, including thermal radiation, visible‐light reflection, and laser echoes, to counter passive detection equipment such as infrared thermal imagers, visible cameras, and lidar systems.

#### 2.2.1 Infrared Stealth Materials

Infrared detectors are energy conversion devices that convert infrared radiation within specific wavebands into measurable electrical signals. Thermal imaging functions by capturing the difference in infrared radiant flux between a target and its surrounding background, which is further converted into two‐dimensional images displayed on a screen. The thermal image contrast C is defined by the disparity of radiant exitance, as shown in Equation (3):

(3) $$C=\frac{E_{0}-E_{b}}{E_{b}}$$

where E_0_ denotes the radiant exitance of the target and E_b_ represents that of the background. A larger contrast value C means the target is easier to be identified by thermal detection. To reduce thermal contrast, it is necessary to narrow the gap of infrared radiation between the target and ambient background. All objects emit spontaneous infrared radiation following the Stefan–Boltzmann law:

(4) $$E=\sigma \varepsilon T^{4}$$

where E is the surface radiant exitance, ε stands for infrared emissivity, σis the Stefan–Boltzmann constant, and T refers to absolute temperature. Two mainstream strategies can realize infrared camouflage via surface radiation regulation: tuning the surface infrared emissivity and adjusting target surface temperature.

##### 2.2.1.1 Infrared Stealth Coatings

**FIGURE 6:**
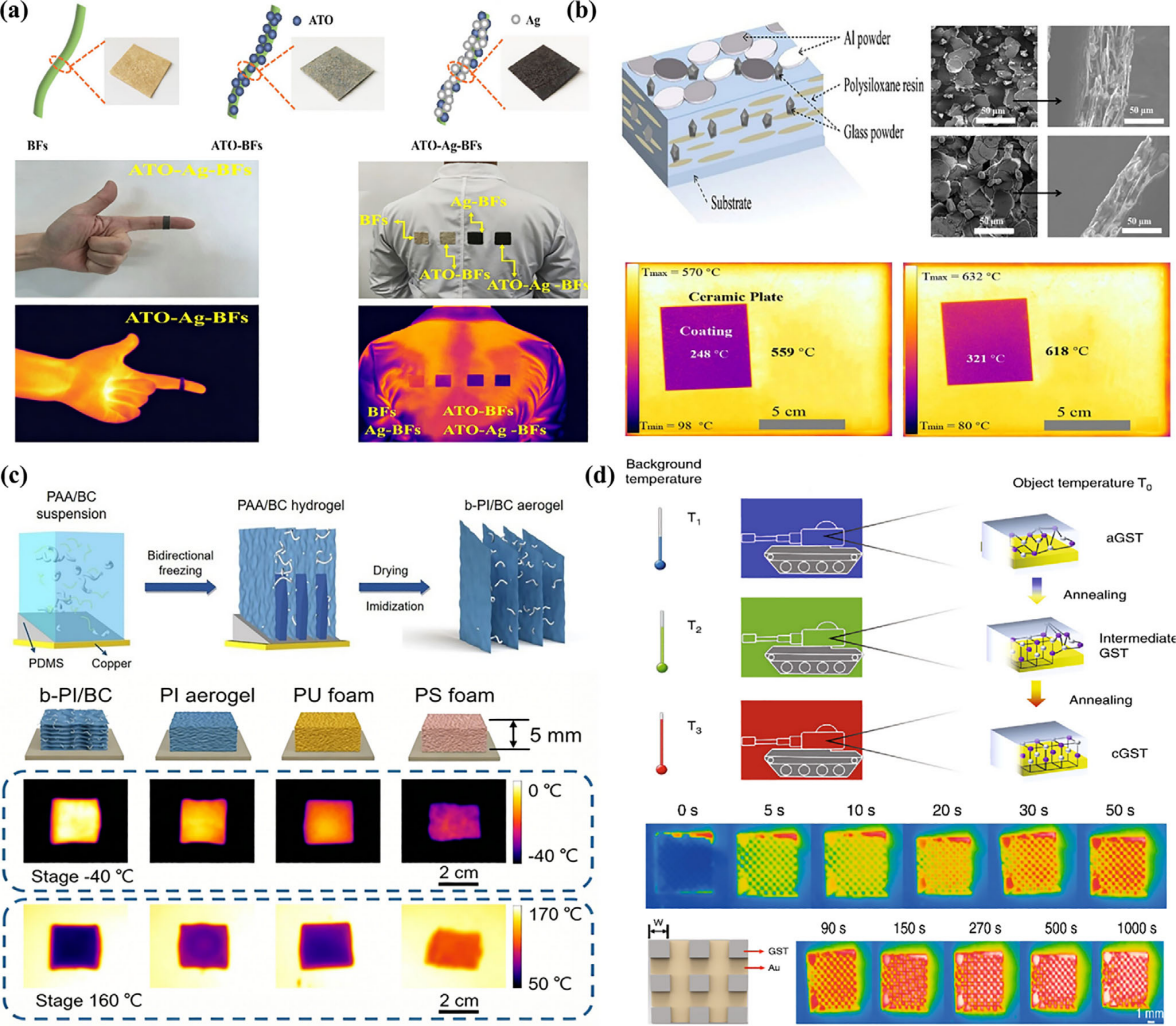
Infrared stealth materials. (a) Sustainable wearable infrared shielding bamboo fiber fabrics loaded with antimony‐doped tin oxide/silver binary nanoparticles. Reprinted with permission [62]. Copyright 2023, Springer Nature. (b) Polysiloxane/Al/glass composite coatings for high‐temperature infrared stealth. Reprinted with permission [63]. Copyright 2023 Elsevier. (c) Bidirectional anisotropic polyimide/bacterial cellulose aerogels by freeze‐drying for super‐thermal insulation. Reprinted with permission [65]. Copyright 2019 Elsevier. (d) Phase‐change GST/Au bilayer structures for dynamic thermal camouflage. Reprinted under the terms and conditions of the Creative Commons Attribution (CC BY) license (https://creativecommons.org/licenses/by/4.0/) [68]. Copyright 2018 The Authors, published by Springer Nature.

Infrared stealth coatings are the most widely adopted stealth media, which consist of low‐emissivity functional fillers and infrared‐transparent binders. Common low‐emissive fillers include metallic pigments, semiconductors, and intrinsically conducting polymers (ICPs). These materials suppress thermal radiation by reflecting most incident infrared light rather than absorbing it. High‐reflection metal powders, such as Al, Zn, Sn, and bronze, serve as core pigments for thermal camouflage coatings. Among them, flaky aluminum powder occupies a dominant position due to high infrared reflectivity and low emissivity. The lamellar stacking structure of aluminum flakes forms a continuous shielding layer inside coatings to lower overall surface emissivity. Jia et al. [61] designed an ultra‐low‐emissive coating for plateau building outer walls, using flaky aluminum as functional filler and two‐component polyurethane as resin matrix with butyl acetate solvent. The coating can efficiently block infrared radiation in the 8–14 µm atmospheric window. Aluminum filler loading is the decisive factor affecting emissivity: at 10 wt%, flakes fail to form a complete shielding network, resulting in high matrix emissivity; when the content rises to 60 wt%, dense oriented stacking delivers an ultra‐low emissivity of 0.035. However, excessive loading up to 80 wt% causes coating warpage and structural defects, pushing emissivity back up to 0.12. Low‐temperature flexibility and thermal cycle tests verify strong interfacial bonding and structural stability. After five thermal cycles, the coating retains over 80% of its original low‐emissive performance in the critical 8–14 µm band, showing great potential for harsh environmental applications. The infrared emissivity of semiconductor fillers is closely correlated with free carrier concentration. Higher carrier density improves carrier mobility and electrical conductivity, thus reducing emissivity. Doping metallic elements is a typical method to increase free carriers for semiconductor infrared pigments. Ye et al. [62] fabricated novel antimony‐doped tin oxide/silver/bamboo fibers (ATO‐Ag‐BFs), as illustrated in Figure 6a. Natural bamboo fiber acts as the substrate, coated with binary ATO‐silver nanoparticle composite films. Schottky junctions generated at the ATO‐Ag interface drive electron transfer from Ag to ATO, drastically increasing interfacial carrier concentration. Meanwhile, abundant free electrons in Ag nanoparticles trigger intense localized surface plasmon resonance. The synergistic effect of the two mechanisms reduces the textile's infrared emissivity to 0.68 within the 8–14 µm band. Thermal imaging experiments prove that the composite can perfectly match target surface temperature with ambient backgrounds, demonstrating excellent flexible wearable infrared camouflage performance.

##### 2.2.1.2 Binders

Binders play a decisive role in film formation and emissivity adjustment. Ideal binders need high infrared transmittance in the 3–5 µm and 8–14 µm atmospheric windows, while meeting strict standards for mechanical strength, environmental durability, and processing feasibility. Binders are divided into inorganic and organic polymer systems. Inorganic binders are mainly silicate, phosphate, borate, metal oxide, and hydroxide cement materials. Their greatest advantage is ultrahigh heat resistance, maintaining structural integrity under hundreds to thousands of degrees Celsius, a property unmatched by organic resins. Therefore, silicate and phosphate inorganic binders are widely studied for ultrahigh‐temperature infrared stealth scenarios such as aero‐engine nozzles and hypersonic vehicle skins. Nevertheless, intrinsic brittleness, poor flexibility, thermal expansion mismatch with metal substrates (which generates internal stress), and complicated high‐temperature sintering procedures restrict their practical application. Organic polymer binders are the most commonly used coating matrixes, with outstanding film‐forming capacity, high flexibility, strong substrate adhesion, and simple fabrication. Most organic polymers exhibit strong intrinsic infrared absorption within atmospheric windows; research thus focuses on developing infrared‐transparent polymers or modifying existing systems via chemical/physical methods to eliminate undesired absorption. Polysiloxanes (silicone resin and silicone rubber) represent the most mature high‐performance organic binders. Their molecular backbone consists of high‐energy Si–Si–O chains with methyl/phenyl side groups. Si–O vibrational absorption appears near 9–10 µm (close to the edge of the 8–14 µm window), while C–H absorption concentrates around 3.4 µm. Adjusting molecular structure and phenyl content enables polysiloxanes to achieve low emissivity across both atmospheric windows. Even so, pure polysiloxanes suffer insufficient mechanical strength and weak high‐temperature adhesion, which can be optimized by blending acrylic/epoxy resin or adding reinforcing fillers. Qi et al. [63] prepared organic–inorganic hybrid AG composite coatings using aluminum flakes, methylphenyl polysiloxane, and glass powder as inorganic reinforcing filler, as shown in Figure 6b. Tuning component thermal evolution realizes coordinated performance improvement. The optimized coating enhances mechanical robustness and raises the wind erosion resistance temperature from 480°C to 600°C, while reducing surface roughness and optimizing infrared optical properties, reaching the minimum emissivity at a moderate high temperature of 590°C.

##### 2.2.1.3 Thermal Insulation Materials

Thermal insulation media suppress heat conduction and narrow the temperature gap between targets and backgrounds to realize infrared camouflage. Novel thermal insulators such as aerogels and hollow microspheres feature ultra‐low thermal conductivity (generally below 0.02 W·m^−1^·K^−1^ at room temperature), with broad prospects for aerospace and military fields. Hollow microspheres possess ultrafine hollow/multi‐layer porous architectures, delivering ultra‐low thermal conductivity and water absorption. As functional fillers, they effectively block heat transfer and lower target infrared radiant exitance. Feng et al. [64] synthesized flexible composite films with a thickness of 0.25 mm, adopting polyaniline/hollow glass microsphere/carbon powder as functional fillers and waterborne polyurethane as binder. The integrated material achieves efficient thermal insulation while retaining visible transparency and mechanical flexibility, with extra functions of ultraviolet shielding and sound absorption. Aerogels with interconnected porous networks exhibit ultra‐low density and excellent thermal insulation. Zhang et al. [65] fabricated bidirectionally anisotropic polyimide/bacterial cellulose (PI/BC) aerogels, as illustrated in Figure 6c. Bidirectional freezing generates oriented layered pore structures, delivering radial thermal conductivity as low as 23 mW·m^−^^1^·K^−^^1^ and axial conductivity of 44 mW·m^−^^1^·K^−^^1^. Doping bacterial cellulose avoids framework shrinkage, endowing the aerogel with lightweight and high mechanical strength. The material realizes intelligent thermal management via radial heat isolation and axial heat conduction, preventing local hot spot accumulation. Ji's research group [66] reported lightweight CuS@rGO aerogels fabricated by hydrothermal reduction, ascorbic‐acid‐assisted thermal reduction, and freeze‐drying. The material integrates radar‐infrared compatible stealth functions. Adjusting CuS loading and reduction procedures optimizes microstructures to form continuous 3D porous networks, granting the aerogel lightweight and reversible compressibility. It maintains an infrared emissivity of 0.6442 across the 3–5 µm and 8–14 µm bands with outstanding infrared camouflage capacity.

##### 2.2.1.4 Phase Change Materials

Phase change materials (PCMs) draw extensive attention for infrared stealth owing to excellent thermal regulation performance. Typical systems include VO_2_ and *Ge_2_Sb_2_Te_5_* (GST). Vanadium dioxide is a classic thermally triggered phase‐change material that undergoes reversible semiconductor–metal transition near 68°C. However, its high phase transition temperature, poor chemical durability, and narrow emissivity tuning range limit practical deployment.

To overcome these defects, Li et al. [67] proposed a multi‐element co‐doping strategy. Five complementary functional elements (W, Mo, Al, Cr, Mn) with equal molar ratios were doped into the VO_2_ lattice via combined hydrothermal and annealing routes to synthesize multi‐element doped VO_2_ (MEVO). High‐valence cations (W^6^^+^, Mo^6^^+^) introduce free electrons to reduce phase transition temperature; low‐valence cations (Al^3^^+^, Cr^3^^+^, Mn^2^^+^) strengthen lattice stability, achieving comprehensive performance optimization. The synthesized MEVO reduces transition temperature to 19.8°C and expands emissivity modulation amplitude to 0.49, maintaining stable performance over 24 h in acidic environments and under 300°C high‐temperature oxidation. Unlike temperature‐driven VO_2_, GST supports precise external electrical control of phase states and shows great potential for active programmable infrared camouflage. Taking this advantage, Qu et al. [68] deposited GST thin films on metal substrates to fabricate thermal camouflage devices, as depicted in Figure 6d. Continuous dynamic adjustment of infrared emissivity is realized by accurately controlling GST crystallization degree via thermal treatment. Experimental results demonstrate that a target fixed at 60°C can thermally match backgrounds ranging from 30°C to 50°C by tuning GST phase states, achieving wide‐range adaptive camouflage. The camouflage effect remains stable over an observation angle of 0° to 60°, and patterned GST films can realize spatial thermal information encoding. This work reveals the great application value of GST‐based phase‐change materials for dynamic, angle‐stable, spatially programmable thermal camouflage, providing guidance for developing next‐generation intelligent infrared stealth technologies.

Among the four main strategies in infrared stealth, low‐emissivity coatings offer simple implementation and excellent high‐temperature stability but provide limited multispectral compatibility; binders determine film‐forming quality and emissivity, with inorganic types excelling in heat resistance yet suffering brittleness, while organic polymers offer flexibility at the cost of intrinsic infrared absorption; thermal insulation materials (aerogels, hollow microspheres) passively suppress thermal contrast without emitting signals but add volume and require encapsulation; phase‐change materials (VO_2_, GST) enable active, programmable emissivity control with nonvolatile memory but face challenges in transition temperature, modulation range, and environmental durability. The choice depends on whether the application prioritizes passive concealment, active tuning, or thermal management.

#### 2.2.2 Visible‐Light Camouflage Materials

Visible‐light camouflage materials serve as core protective media against human visual observation and visible optical reconnaissance. Fundamentally, they modulate target surface optical characteristics, including color, brightness, and texture, to achieve high spectral matching with background environments within the visible spectrum, thereby lowering the recognition risk of visual and optoelectronic detection systems. Effective visible camouflage technologies fall into two complementary technical routes: smoke camouflage screens and material‐based camouflage.

##### 2.2.2.1 Smoke Camouflage Screens

Smoke camouflage screens are traditional countermeasure techniques for visible confrontation. They rapidly generate aerosol and particulate barriers between targets and reconnaissance platforms, shielding objects via visible‐light absorption and scattering. Modern smoke technologies support multispectral‐compatible functions, yet such systems must be equipped with auxiliary early‐warning modules, which adds load on support equipment and limits flexible deployment of smoke interference.

##### 2.2.2.2 Camouflage Coatings and Pattern Design

Conventional visible camouflage coatings adopt thermoplastic acrylic, polyurethane, or silicone resins as binders, mixed with color pigments such as chromium (III) oxide green and titanium dioxide, as well as functional matting fillers including fumed silica and talc. The resulting matte coatings feature glossiness below 10%, which eliminates specular reflection and reduces brightness contrast between targets and surroundings. Pattern design represents another critical module, with digital camouflage as a typical representative. By numerically simulating spectral statistical characteristics of natural scenes such as jungles, deserts, and snowfields, digital camouflage precisely optimizes the shape, area ratio, and spatial distribution of color patches. This blurs target outlines at long distances and disrupts geometric profiles from close‐range observation. Although traditional coating camouflage is easy to implement and takes effect quickly, it requires platforms to remain relatively stationary or operate at low mobility under stable background conditions, which inevitably restricts tactical flexibility.

##### 2.2.2.3 Stimuli‐Responsive Chromic Materials

**FIGURE 7:**
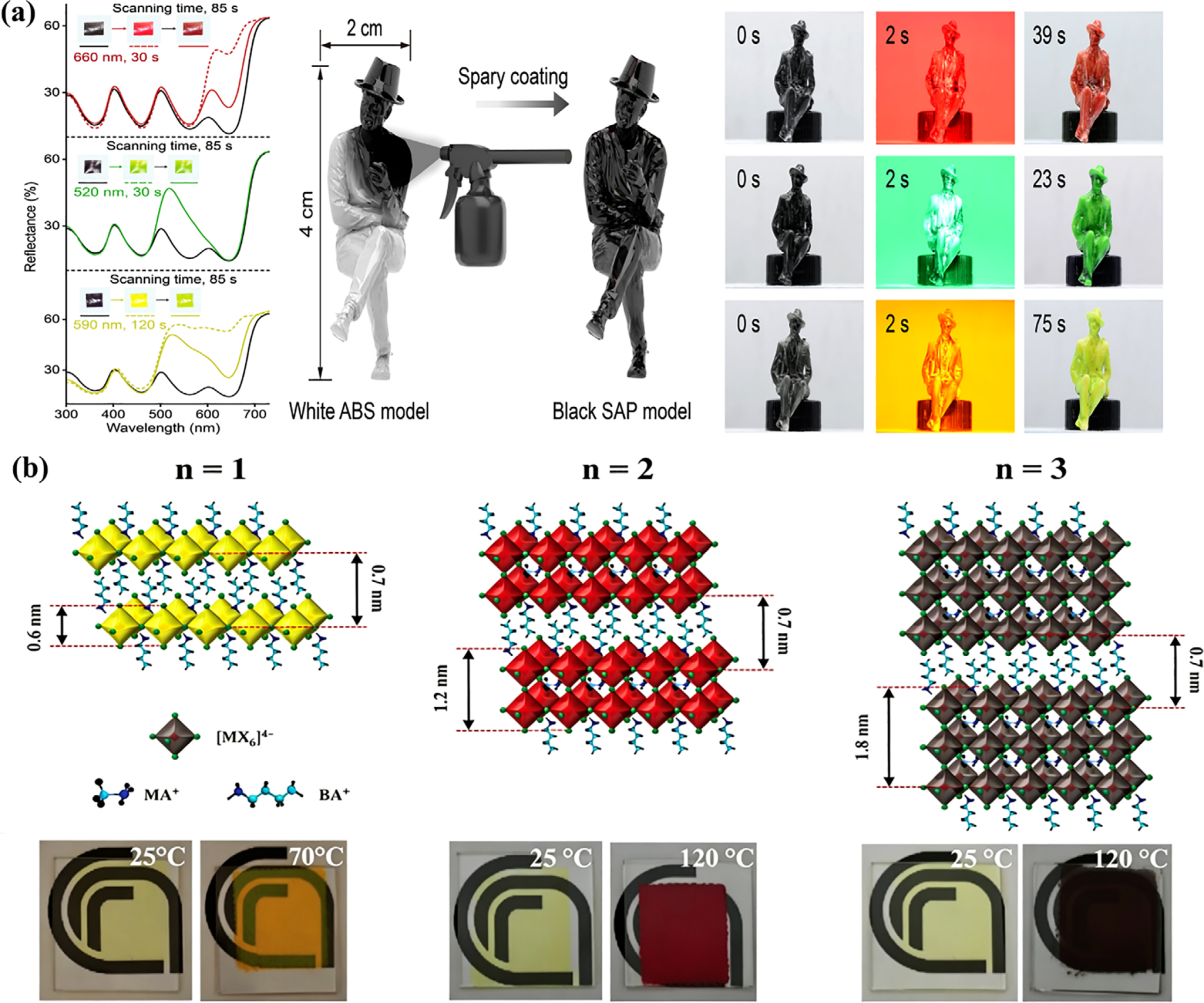
Visible light camouflage materials. (a) Self‐adaptive photochromic materials based on DASA‐organic dye composites. Reprinted under the terms and conditions of the Creative Commons Attribution (CC BY) license (https://creativecommons.org/licenses/by/4.0/) [69]. Copyright 2018 The Authors, published by Science Advances. (b) Thermochromic Printable and Multicolor Polymeric Composite Based on Hybrid Organic–Inorganic Perovskite. Reprinted under the terms and conditions of the Creative Commons Attribution (CC BY) license (https://creativecommons.org/licenses/by/4.0/) [70]. Copyright 2023 The Authors, published by Wiley‐VCH GmbH.

Intelligent chromic materials are developed to adapt to complex dynamic battlefield environments. They can dynamically adjust optical properties under external stimuli such as light, heat, and electricity to realize autonomous background matching camouflage. Photochromic substances undergo reversible molecular rearrangement under specific wavelength irradiation, which alters light absorption spectra and enables switchable color states. Sun et al. [69] fabricated energy‐free adaptive camouflage films based on donor–acceptor Stenhouse adducts (DASAs), as shown in Figure 7a. The material appears dark black under dim environments; upon exposure to natural visible light, DASA molecules experience linear‐to‐cyclic isomerization and form characteristic absorption valleys at designated wavelengths, achieving spontaneous color fusion with surrounding vegetation. Optimizing the mass ratio of DASA and dyes grants the material precise color regulation across 520–660 nm. Experimental tests verify rapid self‐adaptive camouflage in complex plant landscapes, and the system can be processed into flexible films and coatings compatible with diverse substrate surfaces. This research establishes a novel design paradigm that treats active camouflage as an intrinsic material property, offering a low‐cost, lightweight solution for next‐generation intelligent stealth devices. Thermochromic coatings and devices can reversibly change color with fluctuations of surface or ambient temperature to satisfy camouflage demands under thermally variable backgrounds. Perovskite composites stand out as promising thermochromic candidates owing to high visible contrast, fast response, and tunable optical spectra. Cinquino et al. [70] reported printable multicolor polymer composites based on hybrid organic–inorganic perovskites, as illustrated in Figure 7b. The material exhibits prominent thermochromic behavior over 450–800 nm: it maintains transmittance above 80% and high transparency at room temperature, while rapidly turning opaque with transmittance below 10% within 70°C–120°C, with response times on the order of seconds. Different from conventional designs relying on volatile molecule adsorption and desorption, this system adjusts reversible assembly of hybrid organic–inorganic perovskite (PVK) chains via internal hydrogen bonds, maintaining over 80% performance retention after 50 switching cycles. Tuning perovskite film thickness allows precise control of color and phase transition temperature. The printable fabrication technique via inkjet printing enables mass production of intelligent camouflage coatings, providing a versatile material platform for self‐adaptive visible concealment and thermal management integration. Electrochromism refers to the reversible change of optical parameters such as reflectance, transmittance, absorptivity, and emissivity under applied voltage, manifested as switchable color and transparency. Pande et al. [71] designed viologen‐grafted polyhedral oligomeric silsesquioxane (POSS‐viologen) composites for high‐performance red‐green‐blue (RGB) to black multicolor electrochromic switching. Introducing thiophene and phenyl units achieves full coverage of primary RGB hues. Single‐layer devices deliver outstanding electrochromic performance, with a transmittance modulation range up to 67.2%, millisecond‐scale fast response under low driving voltage, and cycling stability exceeding 12 000 times. Stacking multiple RGB functional layers constructs full‐gamut electrochromic devices that reach an extremely low CIELAB luminance parameter L* of 6.99 to realize pure black states for the first time. This work achieves reversible full‐spectrum color tuning under low voltage, providing high‐stability, high‐performance material strategies to meet the core requirements of real‐time background simulation and spectral fusion adaptive visible camouflage.

Among the three stimuli‐responsive chromic materials for visible‐light camouflage, photochromic materials offer passive, autonomous color switching driven by ambient light but suffer from slow response and limited color gamut; thermochromic materials provide fast, printable, large‐area coverage with high contrast yet require temperature fluctuation to activate; electrochromic materials enable full‐gamut, low‐voltage, millisecond‐scale tuning with exceptional cycling stability but demand continuous power supply and complex device architecture. Each mechanism suits different operational scenarios: photochromic for passive ambient‐adaptive concealment, thermochromic for printable large‐area coatings, and electrochromic for precise real‐time programmable camouflage.

Conventional wave‐absorbing materials have seen broad research progress, yet fundamental physical rules create several performance restrictions.

The Rozanov criterion affects low‐frequency stealth performance. Long radar wavelengths tend to demand thick absorbing layers to generate adequate wave coupling. Researchers have designed multiscale composite structures to boost absorption within limited thickness. Material intrinsic traits can still restrict overall absorption capacity. Ultra‐wideband high‐efficiency absorption may be hard to achieve under thin‐layer constraints. Thick stacked structures also conflict with lightweight design standards for aerial equipment platforms.

Magnetic absorbing materials are affected by the Snoek limit. Magnetic permeability declines as working frequency increases, weakening high‐frequency energy loss. Anisotropic micro‐particles and multi‐phase composites can add compound loss channels. Such modified materials tend to carry extra mass and stiffness, which limits their use on thin, curved carrier surfaces.

Most traditional absorbers maintain fixed spectral response after processing. Their stealth capacity cannot adapt to variable detection frequencies and shifting field backgrounds. Phase‐change and color‐adjustable media like VO_2_ can offer partial infrared and visible band regulation. The material switches between insulator and metal states at fixed temperature points, and emissivity only jumps between discrete values. Continuous intermediate adjustment is hard to achieve with such systems. Their adjustable spectral range stays narrow, and external power input may be required to sustain stable radiation characteristics.

## 3 Metasurface‐Based EM Wave Modulation Materials

### 3.1 Modulation Mechanisms of Metasurfaces

The core of EM low‐detectability technology lies in flexible, precise manipulation of EM waves. Conventional stealth media such as ferrites and carbon composites rely on intrinsic loss mechanisms of three‐dimensional bulk materials; their overall performance is inherently capped by fixed chemical compositions and crystal structures. In sharp contrast, metasurfaces bring a shift in design philosophy. By compressing the design dimension from 3D bulk volumes to planar two‐dimensional interfaces, metasurfaces enable programmable EM wave control via artificially engineered subwavelength meta‐atoms. Their theoretical framework is built upon the equivalence principle and surface impedance model derived from EM boundary theory.

i. Paradigm Shift: From “Searching for Natural Materials” to “Designing Artificial Microstructures”

Traditional research focuses on screening or synthesizing bulk substances with ideal complex permittivity ε and complex permeability μ, which is limited by the inherent properties of natural materials. Metasurfaces break this restriction by adopting Generalized Sheet Transition Conditions (GSTCs) as the core equivalence principle. A periodic metasurface array can be macroscopically approximated as an infinitely thin sheet, and field jumps across the interface are governed by electric surface susceptibility χ_*ES*_ and magnetic surface susceptibility χ_*MS*_:

$$n\;\times \;\Delta H=j\omega \varepsilon \chi_{\mathrm{ES}}\cdot E_{t,\mathrm{av}}$$

(5) $$n\;\times \;\Delta E=-j\omega \mu \chi_{\mathrm{MS}}\cdot H_{t,\mathrm{av}}$$

where *E*_*t*,*av*_ and *H*_*t*,*av*_ denote the average tangential electric and magnetic fields; χ_*ES*_ and χ_*MS*_ characterize the EM response of meta‐atoms, determined by geometric layout, areal density N, and electric/magnetic polarizabilities α_e_ and α_m_ [72]:

$${\chi_{ES}}^{xx}=\frac{N\langle \alpha_{e,xx}\rangle }{1-\left(\frac{N\langle \alpha_{e,xx}\rangle }{4r}\right)}$$

(6) $${\chi_{MS}}^{xx}=\frac{-N\langle \alpha_{m,xx}\rangle }{1+\left(\frac{N\langle \alpha_{e,xx}\rangle }{4r}\right)}\;$$

here, r stands for the inter‐unit interaction constant, and areal density satisfies *N* = 1/*p* ^2^ (p = lattice period). These formulas reveal how incident EM fields induce surface polarization via intrinsic polarizabilities α_e_ and α_m_, forming a complete physical description of field‐current conversion. Although the equivalent source model clarifies the underlying physics, direct tuning of χ_*ES*_ and χ_*MS*_ is overly complicated for full‐wave wavefront design. For engineering simplification, the Leontovich surface impedance boundary condition is widely adopted, which reduces complex multi‐parameter design to spatial modulation of scalar surface impedance *Zs*. The surface impedance *Z_s_* and surface admittance *Y_s_* are defined as:

$$\mathrm{Zs}\;\left(x,y\right)=j\omega \mu \chi \mathrm{MS}$$

(7) $$\mathrm{Ys}\;\left(x,y\right)=j\omega \varepsilon \chi \mathrm{ES}$$

The Leontovich impedance boundary condition is expressed as:

$$E_{t}=Z_{s}\;\hat{n}\;\times \;H_{t}$$

(8) $$Z_{s}=R+jX$$

where *E_t_* and *H_t_* are tangential field components, and $\hat{n}$ is the unit normal vector pointing outward from the metasurface. *Z_s_* consists of a resistive real part R and a reactive imaginary part X. All EM responses of a metasurface can be fully described by the spatially varying distribution *Z_s_* (*x*,*y*). Therefore, EM field design is converted into a simple 2D impedance patterning task. Optimizing meta‐atom geometry and areal density allows direct tuning of χ_*ES*_ and χ_*MS*_. This paradigm transition abandons passive screening of bulk materials and turns to active design of planar equivalent boundaries, delivering expanded design freedom and facilitating the development of ultrathin, broadband EM devices.

ii. Functional Mechanism: From Passive Energy Dissipation to Active EM Wave Programming

Conventional loss‐type absorbers operate via passive energy dissipation, where incident EM energy is converted into heat through dielectric and magnetic relaxation. Their optimization faces an irreconcilable trade‐off: strong dielectric/magnetic loss easily causes severe impedance mismatch and high surface reflection, while perfect impedance matching inevitably demands thicker substrates, falling into the bandwidth‐thickness limitation defined by the Rozanov criterion. Metasurfaces overcome this dilemma by precise surface impedance engineering to realize decoupled, synergistic control over wave absorption and propagation.

Perfect absorption: Traditional bulk materials can hardly simultaneously satisfy strong intrinsic loss and ideal impedance matching. In contrast, customized resonant modes in meta‐atoms enable the real part of equivalent surface resistance to match free‐space impedance *Z*_0_ ≈ 377 Ω, while the imaginary reactance X approaches zero, achieving conjugate matching between metasurfaces and free space:

(9) $$Z_{s}=Z_{0}\;\Rightarrow \Gamma =\frac{Z_{s}-Z_{0}}{Z_{s}+Z_{0}\;}=0$$

Chen et al. [73] theoretically proposed and experimentally verified a dual‐band perfect absorber based on dielectric‐metal hybrid metasurfaces for mid‐infrared photodetectors. The structure comprises a lossless silicon grating stacked above a thin gold reflective layer. Guided‐mode resonance in the dielectric layer tightly confines optical energy and couples it into the gold film for efficient thermal dissipation. After geometric optimization, the metasurface achieves near‐perfect absorption rates of 97.5% and 99.7% at wavelengths 6.142 and 7.795 µm, respectively.

Wavefront shaping: Traditional wavefront manipulation devices such as lenses and prisms rely on optical path accumulation during wave propagation, requiring physical thickness and aperture far larger than the operating wavelength. Metasurfaces realize anomalous reflection/refraction, beam focusing and vortex beam generation by introducing spatially inhomogeneous phase delay distribution. Within the impedance model, the phase correlates directly with the reactive component of the impedance; consequently, its spatial gradient ∇X(x, y) maps directly to the additional phase gradient ∇Φ.

(10) $$\nabla \Phi \;\approx \;K_{0}\cdot \frac{\nabla X}{Z_{0}}$$

where $k_{0}=\frac{2\pi }{\lambda_{0}}$ denotes the free‐space wave number and *Z*_0_ is free‐space impedance. This extra tangential wave vector ∇Φ breaks conventional wavevector conservation and leads to the generalized Snell's law [74].

(11) $$\sin\left(\theta_{t}\right)-\sin\;\left(\theta_{i}\right)=\frac{\lambda_{0}}{2\pi n_{i}}\;\frac{d\Phi }{\mathrm{dx}}$$

Equations (10) and (11) indicate that spatial variation of reactance X yields continuous phase modulation of EM waves. Arbitrary wavefront manipulation (beam deflection, focusing, vortex excitation) can be realized on ultrathin planar platforms, breaking the wavelength‐scale thickness restriction of classic optical elements down to the subwavelength regime.

iii. Performance Advancement: From Static Coatings to Programmable Metasurface Skins

The inherent drawbacks of traditional stealth materials originate from their static material nature: fixed frequency responses after fabrication, excessive mass and volume especially for magnetic substrates, and single functionality that cannot satisfy multispectral stealth demands covering radar, infrared and other bands. These defects severely restrict their deployment on modern lightweight, multifunctional platforms. Metasurfaces deliver advantages to resolve the above limitations.

Ultrathin and Lightweight Configuration: Ultrathin, lightweight design is a core demand for aerospace optoelectronic systems, and metasurfaces provide a viable solution. Yao et al. [75] designed an electrically tunable perfect absorber integrating graphene into ultrathin Fabry–Pérot resonant cavities to realize dynamically adjustable critical coupling. The overall device thickness falls into the deep‐subwavelength range below λ/10, with a modulation depth over 95% and a predicted tunable bandwidth up to 20 GHz. This work validates the huge potential of ultrathin reconfigurable optoelectronic devices. The planar, lightweight layout meets strict aerodynamic contour, weight control and multifunctional integration requirements for aerospace carriers.

**FIGURE 8:**
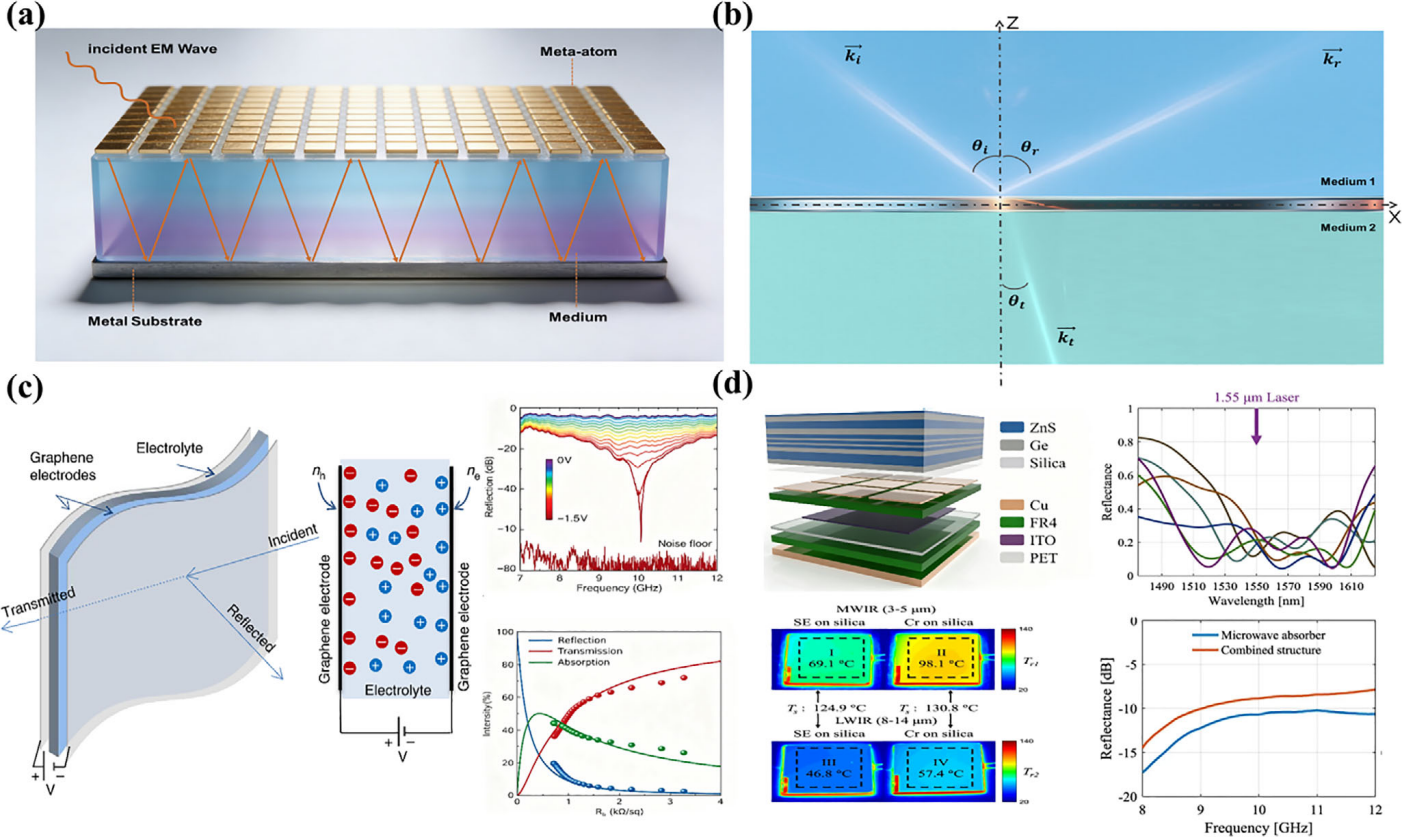
Metasurface‐Based EM Wave Regulation Materials. (a) Schematic illustration of the sandwich‐structure perfect absorber. (b) Schematic diagram of the generalized Snell's law. (c) Dynamic regulation capability: Graphene‐enabled electrically switchable radar‐absorbing surfaces. Reprinted under the terms and conditions of the Creative Commons Attribution (CC BY) license (https://creativecommons.org/licenses/by/4.0/) [76]. Copyright 2021 The Authors, published by Springer Nature. (d) ZnS/Ge multilayer and Cu‐ITO‐Cu metasurface for multispectral camouflage. Reprinted under the terms and conditions of the Creative Commons Attribution (CC BY) license (https://creativecommons.org/licenses/by/4.0/) [77]. Copyright 2021 The Authors, published by Springer Nature.

Dynamic reconfigurability: Conventional materials possess fixed EM responses after processing. By embedding active media (graphene, liquid crystals, phase‐change materials) into meta‐atoms, surface impedance Z_s_ can be dynamically tuned under electrical, thermal or optical excitation, enabling real‐time switching of stealth bandwidth and scattering patterns for intelligent camouflage. A representative study was reported by Balci et al. [76] who fabricated electrically switchable radar‐absorbing metasurfaces. Electrostatic modulation adjusts carrier density on graphene electrodes to dynamically control microwave reflection, transmission and absorption, as shown in Figure 8c. Experimental data show the metasurface reaches a reflection attenuation depth up to 50 dB under operating voltages lower than 5 V, with wide dynamic range and fast response characteristics.

Multispectral compatibility: By integrating multiscale micro‐nano units on a single ultrathin plane, metasurfaces can independently tune EM phase, amplitude and polarization at nanoscale dimensions. Rational impedance design separates the real (absorption) and imaginary (phase) contributions of surface impedance, allowing simultaneous microwave absorption and wavefront control on one planar layer. Furthermore, optimized material and structural layouts support efficient operation across visible‐to‐microwave broadband spectra. Zhu et al. [77] proposed a multilayer ZnS/Ge selective emitter (SE) combined with Cu‐ITO‐Cu microwave‐absorbing metasurfaces for multi‐band spectral regulation, as illustrated in Figure 8d. In the infrared band, multilayer thin films achieve low emissivity within atmospheric windows and high emissivity outside windows for passive radiative cooling. Tuning the thickness of the top ZnS layer generates structural colors matching soil, desert, aquatic and vegetation backgrounds in the visible region. In the microwave regime, the Cu‐ITO‐Cu metasurface attains over 90% wave absorption efficiency and effectively suppresses RCS.

**FIGURE 9:**
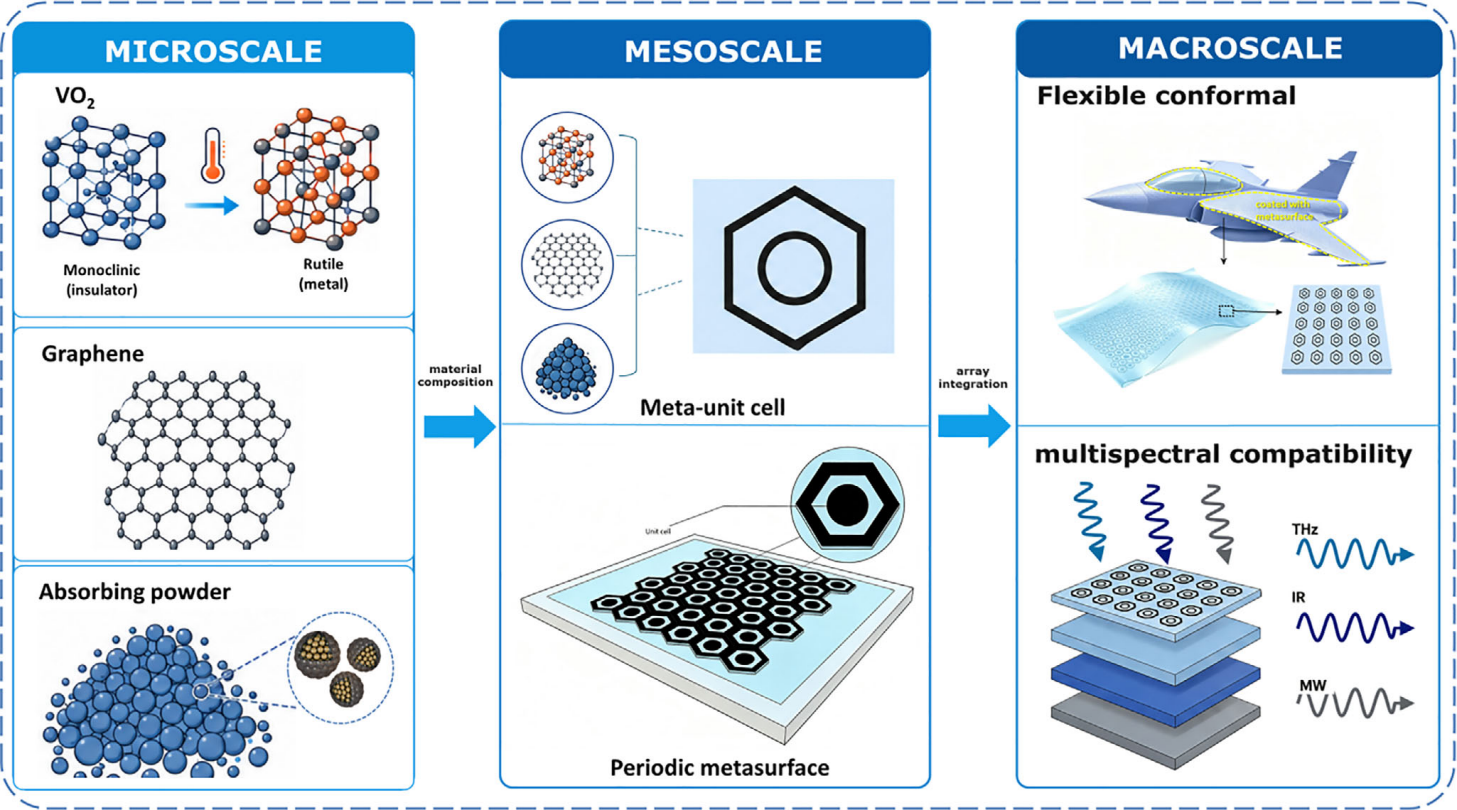
Schematic illustration of cross‐scale material–structure synergy for EM wave manipulation in two‐dimensional metasurfaces.

The remarkable stealth performance of metasurfaces is enabled by cross‐scale material–structure synergy, as illustrated in Figure 9. Material composition governs the intrinsic EM loss and tunable properties at the molecular scale, while the geometry and topology of microscale meta‐atoms regulate localized resonance, impedance matching, and energy dissipation. At the microscale, the arrangement and near‐field coupling of meta‐unit cells provide additional control over the phase, amplitude, polarization, and spatial dispersion of EM waves. At the macroscale, the arrangement of meta‐atom arrays, multilayer architectures, and conformal integration further tailors the global scattering and wavefront behaviors. This multiscale coordination fundamentally surpasses the Rozanov and Snoek limits of conventional single‐scale bulk stealth materials. In summary, metasurface‐based EM modulation transforms the traditional passive dissipation paradigm of three‐dimensional bulk materials into active two‐dimensional surface‐impedance programming. This paradigm shift overcomes the inherent bottlenecks of conventional stealth systems in thickness, weight, and multispectral adaptability, laying a solid physical foundation for next‐generation lightweight, dynamically tunable, and multifunctional stealth technologies.

### 3.2 Various Metasurfaces for Stealth Applications

#### 3.2.1 Electromagnetic Metasurfaces

Radar stealth aims to reduce the RCS of targets via EM wave absorption or diffuse scattering, thus lowering detection probability. RCS quantifies a target's ability to scatter EM signals toward radar receivers under incident illumination, defined as:

(12) $$\lim_{R\to \infty }4\pi R^{2}\frac{I_{r}}{I_{i}}=\lim_{R\to \infty }4\pi R^{2}\frac{{\left|E_{r}\right|}^{2}}{{\left|E_{i}\right|}^{2}}\;$$

where R denotes the distance from the target to the receiving antenna; *I_i_* and *I_r_* represent the incident and scattered power flux densities, respectively; *E_i_* and *E_r_* are the incident electric field intensity at the target and the scattered electric field intensity at the receiver, respectively.

From this definition, smaller RCS shortens the detectable radar range and suppresses target identification. Radar cross‐section reduction can be physically realized via two routes: full EM energy dissipation by absorbers, or redirecting scattered waves to multiple azimuths to dilute the echo signature captured by radar detector. RCS magnitude depends on surface conductivity, geometric profile, material composition and incident angle, and is independent of transmitting power and target‐radar separation. Classic radar stealth strategies include optimized aerodynamic shaping, wave‐absorbing coatings to suppress reflected signals, and passive/active phase cancellation via 180° out‐of‐phase reflected waves. To date, microwave and terahertz stealth metasurfaces have been extensively studied. Multifunctional metasurface modules such as optically transparent microwave absorbers and low‐infrared‐emissivity attenuators expand applicable scenarios, yet early static metasurfaces with fixed functions fail to adapt to agile intelligent detection systems. Therefore, tunable, reconfigurable metasurfaces have become an active area of research.

EM wave absorption describes the conversion of incident EM energy into Joule heat within specified bands by high‐loss resonant architectures, which simultaneously suppress reflection and transmission. For absorbing structures, absorption efficiency A(ω) is determined by reflectivity R(ω)) and transmissivity T(ω):

(13) $$A(\omega )=1-R(\omega )-T(\omega )=1-{\left|S_{11}\right|}^{2}-{\left|S_{22}\right|}^{2}$$

where R(ω) integrates co‐ and cross‐polarized reflection, while T(ω) sums all transmission components. Most metasurface absorbers adopt continuous metallic ground planes to block transmission (T≈0), so reflectivity dominates wave attenuation performance:

(14) $$\begin{array}{c} R(\omega )={\left|S_{11}\right|}^{2}={\left|\frac{Z_{\mathrm{in}}-Z_{0}}{Z_{\mathrm{in}}+Z_{0}}\right|}^{2}=\frac{{\left[Re(Z_{in})-Z_{0}\cos\theta \right]}^{2}+{\left[Im(Z_{in})\right]}^{2}}{{\left[Re(Z_{in})+Z_{0}\cos\theta \right]}^{2}+{\left[Im(Z_{in})\right]}^{2}} \end{array}$$

Z_0_ ≈ 377Ω denotes free‐space intrinsic impedance, and Z_in_ is the input impedance determined by meta‐atom geometry.

In 2008, Landy et al. [78] proposed the first ultrathin subwavelength perfect absorber, consisting of top split‐ring resonators, intermediate dielectric spacer and bottom metallic strips. Its absorption mechanism originates from electric resonance of split rings and magnetic coupling between top and bottom metal layers. Optimized unit geometry realizes impedance matching at target frequency, delivering 88% absorption at 11.5 GHz with thickness merely 0.2 mm (far below the operating wavelength). However, this pioneering design suffers narrow bandwidth and polarization sensitivity. On this basis, researchers worldwide have promoted absorbing metasurfaces toward polarization insensitivity, ultra‐broadband response and lightweight design [79, 80, 81]. To satisfy complex dynamic EM environments and reduce maintenance costs, reconfigurable active absorbing metasurfaces have attracted growing attention.

##### 3.2.1.1 Origami Metasurfaces Driven by Smart Responsive Materials

**FIGURE 10:**
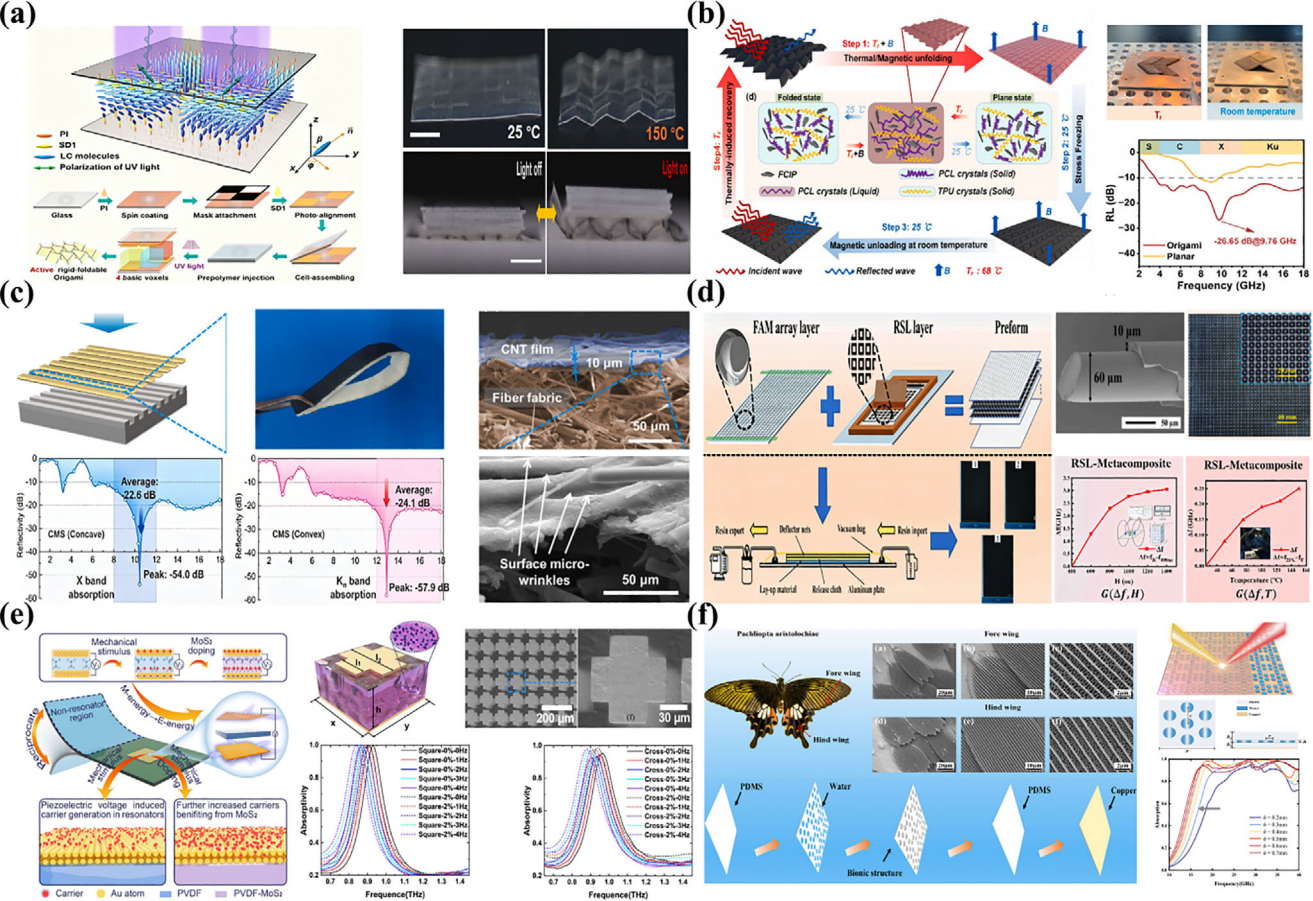
Electromagnetic metasurface. (a) Programmable Liquid‐Crystal‐Elastomer‐Based Reconfigurable Rigid‐Foldable Origami. Reprinted with permission [83]. Copyright 2025 Wiley‐VCH GmbH. (b) Thermally and magnetically tunable origami microwave absorber based on shape memory polymer composite. Reprinted with permission [84]. Copyright 2025 Elsevier. (c) Reconfigurable cementitious metastructure integrated with carbon nanotube frequency selective surface for tunable microwave absorption. Reprinted with permission [86]. Copyright 2025 Elsevier. (d) Ferromagnetic Amorphous Microwire/Resistive Loop Reinforced Multifunctional Metacomposite Absorber. Reprinted with permission [87]. Copyright 2026 Elsevier. (e) Synergistic design of electro‐responsive materials and FSS: Mechanically tunable terahertz metamaterial absorber based on piezoelectric effect of PVDF‐MoS2 composite films. Reprinted with permission [90]. Copyright 2025 Wiley‐VCH. (f) Structural design integrating microfluidic channels: Biomimetic water‐based metamaterial absorber for ultrabroadband microwave absorption. Reprinted with permission [91]. Copyright 2025 Wiley‐VCH.

Origami technology converts planar films into elaborate three‐dimensional architectures, providing an innovative metasurface design route [82]. By adjusting folding patterns and dihedral angles, origami meta‐atoms undergo geometric deformation to dynamically regulate absorption. Such structures employ origami lattices as skeletons and adopt thermo/photoresponsive smart media including liquid crystal elastomers (LCEs) and shape‐memory polymers (SMPs) as actuation layers. Geometric reconstruction reshapes resonant modes to realize contactless broadband modulation while retaining lightweight, highly reconfigurable merits. Wu et al. [83] fabricated programmable rigid foldable Miura‐ori metasurfaces based on LCE, as illustrated in Figure 10a. Combined planar/vertical photoalignment achieves precise molecular arrangement in single LCE films, enabling pixelated units with controllable folding curvature and deformation dynamics. The LCE origami microwave absorber bears load 46.8 times its own weight, and near‐infrared light triggers reflection tuning with a modulation depth of 8.81 dB at 24.28 GHz. Different from optically controlled LCE systems, thermomagnetic SMP origami achieves electricity‐free deformation. Yin et al. [84] designed dual‐field tunable origami absorbers using TPU/PCL shape‐memory matrix filled with flaky carbonyl iron and reduced graphene oxide to construct magneto‐dielectric loss systems, as shown in Figure 10b. Optimized via genetic algorithms, the 3.4 mm‐thick unit switches reversibly between planar and folded states under 68°C heating plus magnetic excitation. The folded multi‐facet structure generates multiple EM scattering, combined with eddy current and polarization loss, yielding ultra‐broad effective absorption covering 3.6–18.0 GHz with RLmin = −26.65 dB; the planar state only supports narrowband absorption from 7.76–9.76 GHz. Contactless tuning makes it suitable for intelligent absorption devices. For transparent low‐cost scenarios, flexible ITO‐PET films are ideal origami substrates. Song et al. [85] fabricated laser‐etched Miura‐ori foldable metasurfaces with 87.2% visible transmittance, supporting reflection modulation over 4.96–38.8 GHz, modulation depth over 10 dB and fractional bandwidth up to 155%. The material is ultra‐light with a cost of only 16 USD, promising applications for satellite optical windows.

Origami metasurfaces support contactless broadband modulation and multi‐state reconfigurability with lightweight structural features. They are suitable for space‐borne platforms that pursue simple and lightweight configurations. Thermal and optical actuation limits their response speed, and repeated folding may cause structural fatigue. Stable operation relies on precise environmental control, increasing system complexity. These metasurfaces fit scenarios with intermittent reconfiguration demands but are incompatible with real‐time, high‐frequency EM switching applications.

##### 3.2.1.2 Composite Lossy Materials & Reconfigurable Structure Synergy

This category integrates dielectric/magnetic loss composites with reconfigurable frameworks such as three‐dimensional gratings, frequency selective surfaces (FSS) and vacuum‐assisted resin transfer molding (VARTM) structures. Multi‐field coupling and mechanical deformation realize dynamic EM tuning; loss media dominate energy dissipation while reconfigurable lattices optimize wave propagation paths, simultaneously offering mechanical load‐bearing capacity and extreme‐environment durability. Wang et al. [86] designed CNT‐FSS/cement metastructures with 3D grating substrates, as illustrated in Figure 10c. The cement grating concentrates incident EM waves, while attachable CNT‐FSS enables targeted selective absorption. Uniform CNT networks form continuous loss channels to attenuate EM energy, and rigid cement substrates guarantee long‐term structural stability. The composite achieves effective absorption across 5.8–18 GHz, and resonant peaks can be dynamically switched by flipping the FSS layer. To introduce magnetothermal dual tuning beyond single loss mechanisms, Yang et al. [87] fabricated ferromagnetic amorphous microwire composite absorbers via VARTM, as shown in Figure 10d. The composite integrates magnetic microwires, carbon resistive loops and glass fiber epoxy, delivering over 60% average absorption across X‐band and above 90% absorption within 9.7–11.9 GHz, alongside axial tensile strength of 487.5 MPa and thermal stability up to 163.41°C. Magnetic excitation blue‐shifts absorption peaks by 2.31 GHz, while thermal stimulation induces a 0.25 GHz red shift, matching aerospace extreme service demands.

Lossy composite structures with reconfigurable frameworks integrate EM absorption, mechanical load‐bearing capacity and multi‐field tunability. They adapt to harsh operating conditions and are viable for aerospace components requiring high structural stability and broadband stealth performance. These structures involve sophisticated fabrication procedures, which increase manufacturing costs. Embedded functional fillers also raise structural weight. Material inherent properties may constrain the tuning range, and the reconfiguration speed remains moderate. Such designs favor engineering scenarios with high durability requirements, yet are less suitable for weight‐sensitive and fast‐tuning platforms.

##### 3.2.1.3 Electrically Responsive Material & FSS Co‐Design

This type of metasurface combines voltage‐tunable media (PIN diodes, graphene, piezoelectrics) with FSS lattices. Applied voltage adjusts conductivity or piezoelectric strain to precisely modulate EM responses; responsive layers control tuning range and sensitivity while FSS optimizes frequency selectivity, featuring fast response and high precision for high‐frequency stealth and communication coexistence. Over the past two decades, lumped components (PIN/varactor diodes, resistors, capacitors) have become mainstream reconfiguration elements for microwave circuits such as antennas and filters, with mature manufacturing, fast switching and wide tuning ranges. Yu et al. [88] proposed high‐selectivity frequency‐selective resonators (FSR). The unit adopts low‐loss F4B dielectric and PMI foam spacer with patterned metal layers. PIN diode variable resistance realizes dynamic absorption‐transmission switching: a 1 dB transmission window at 8.43–8.51 GHz and absorptivity above 0.9 across 4.43–13.45 GHz. Graphene is another outstanding tuning medium. Gate voltage modulates carrier density and sheet conductivity to adjust absorption/reflection, with picosecond response, microwave‐to‐terahertz broadband coverage, mechanical flexibility and CMOS compatibility. Peng et al. [89] designed graphene‐shape memory alloy (SMA) reconfigurable absorbers. Graphene films tune reflection amplitude via bias voltage, while SMA springs adjust cavity height to shift resonant frequency, supporting three working modes: ultra‐broadband absorption, tunable narrowband absorption and amplitude‐modulated absorption. The device absorbs 3.6–18 GHz microwaves and dynamically shifts narrowband peaks within 8.7–13.5 GHz while maintaining polarization insensitivity. Piezoelectric terahertz metasurfaces solve mechanical tuning's high‐frequency limitations. Sun et al. [90] constructed polyvinylidene fluoride (PVDF)‐MoS_2_ piezoelectric tunable absorbers, as shown in Figure 10e. Mechanical excitation generates built‐in voltage via piezoelectric effect to adjust interfacial carrier concentration and equivalent permittivity, continuously red‐shifting absorption peaks without deforming resonators. The maximum frequency offset reaches 62 GHz under 0–4 Hz mechanical excitation, with absorption kept above 99.6% throughout tuning. The non‐contact modulation strategy ensures stable performance after 4000 mechanical cycles with negligible attenuation.

Electrically responsive metasurfaces integrated with frequency selective surfaces enable high‐precision, fast EM modulation at microsecond‐to‐millisecond scales. They support low‐voltage tunability for real‐time radar stealth and adaptive EM regulation. This technology requires continuous power supply and auxiliary control circuits, which increase system complexity and power consumption. Lumped tuning elements may limit modulation resolution. Such metasurfaces are suitable for conventional ground and vehicle‐borne platforms, but are less applicable for ultra‐thin, flexible and low‐power lightweight systems.

##### 3.2.1.4 Microfluidic Channel Integrated Metasurfaces

Liquid media serve as core functional layers embedded in microfluidic architectures. Fluid permittivity and flow characteristics enable reversible contactless EM modulation, featuring simple fabrication, low cost and optical transparency. Inspired by multi‐stage porous butterfly wing structures, Ji et al. [91] proposed water‐based biomimetic metamaterials, as illustrated in Figure 10f. Water acts as tunable dielectric loss medium; polydimethylsiloxane (PDMS) mimics biological epidermis to construct gradient impedance frameworks with hexagonal water cavities. Multiscale resonance and extended wave paths achieve average absorptivity over 95% within 17.11–35.74 GHz at thickness only 2.2 mm, with polarization insensitivity and wide‐angle stability. Further integration of transparent conductors and liquid substrates expands microfluidic applications. Ge et al. [92] designed double‐layer ITO water‐substrate metasurfaces fabricated via magnetron sputtering and laser patterning. Adjusting water thickness switches the device between ultra‐broadband and dual‐narrowband modes, with fractional bandwidth up to 154.9% and average visible transmittance of 60.49%. The complementary advantages of ITO and liquid media break the bottleneck of simultaneous transparency, broad bandwidth and dynamic tuning.

Microfluidic metasurfaces utilize tunable liquid permittivity to achieve broadband EM absorption and optical transparency with low‐cost fabrication. They are applicable to transparent and conformal platforms requiring dual EM and optical performance. The fluid tuning mechanism enables reversible, contactless modulation. This design suffers from slow response speed and relies on auxiliary fluidic components, which complicates system integration. Liquid working media also bring reliability risks under variable temperatures.

To provide an intuitive comparison of different technical approaches for EM stealth metasurfaces, Table 1 summarizes the tuning categories and achievable radar absorption performance of representative works, highlighting the trade‐offs among different strategies.

**TABLE 1:** Comparison of Reported Electromagnetic Stealth Metasurfaces.

Refs.	Technical Approach	Microstructure Design	Tuning Category	Tunable Radar Absorption Performance
[83]	Origami Metasurfaces Driven by Smart Responsive Materials	LCE Miura‐ori	Amplitude modulation	Reflectivity tuning @24.28 GHz, Δσ = 8.81 dB
[84]		SMP origami FCIP+rGO	Mode switching	Mode switching: Planar (7.76–9.76 GHz) → Folded (3.60–18.00 GHz); RL_min_ = –26.65 dB
[85]		ITO‐PET origami	Amplitude modulation	Reflectivity tuning (4.96–38.8 GHz), Δσ > 10 dB, fractional BW = 155%

[86]	Composite Lossy Materials & Reconfigurable Structure Synergy	CNT‐FSS / Cementitious metastructure (CMS)	Mode switching	Mode switching: Suspend (5.8–18 GHz) → Concave X‐band (RL_min_ –54.0 dB @ 10.42 GHz) → Convex Ku‐band (RL_min_ –57.9 dB @ 12.94 GHz)
[87]		Microwire / RSL / Glass‐epoxy VARTM	Frequency modulation	Frequency tuning: Δf peak = +2.31 GHz (10.5 → 12.81 GHz, blue shift under magnetic field)

[88]	Electrically Responsive Material & FSS Co‐Design	PIN diode + FSS AFSS/ACRs	Amplitude modulation	Absorptivity tuning: ΔA = 0.9 @ 4.43–13.45 GHz; 1 dB transmission @ 8.43–8.51 GHz
[89]		Graphene + SMA cavity	Mode switching	Mode: Wideband (3.6–18 GHz) → Narrowband (8.7–13.5 GHz); Freq. tuning: Δf = 4.8 GHz;
[90]		PVDF‐MoS_2_ piezoelectric	Frequency modulation	Frequency tuning: Δf = 62 GHz; absorptivity >99.6%

[91]	Microfluidic Channel Integrated Metasurfaces	Water/PDMS hexagonal cavities	Amplitude modulation	Absorptivity tuning: ΔA = 0.57 (39.1% → 96.2%) @ 17.11–35.74 GHz
[92]		ITO / Water substrate	Mode switching	Mode switching: UWB absorption (12.49–98.21 GHz) ↔ Dual‐band absorption (12.77–17.81 / 37.97–71.57 GHz)

#### 3.2.2 Thermal Camouflage Metasurfaces

The fundamental physical principle of thermal camouflage is governed by the Stefan–Boltzmann law of thermal radiation. This law states that the total thermal radiant exitance emitted from a solid surface is proportional to the surface temperature raised to the fourth power and the material's infrared emissivity [93]:

(15) $$W=\sigma \varepsilon T^{4}$$

where W denotes surface radiant exitance, ε stands for infrared emissivity, σ is the Stefan–Boltzmann constant, and T represents absolute temperature.

**FIGURE 11:**
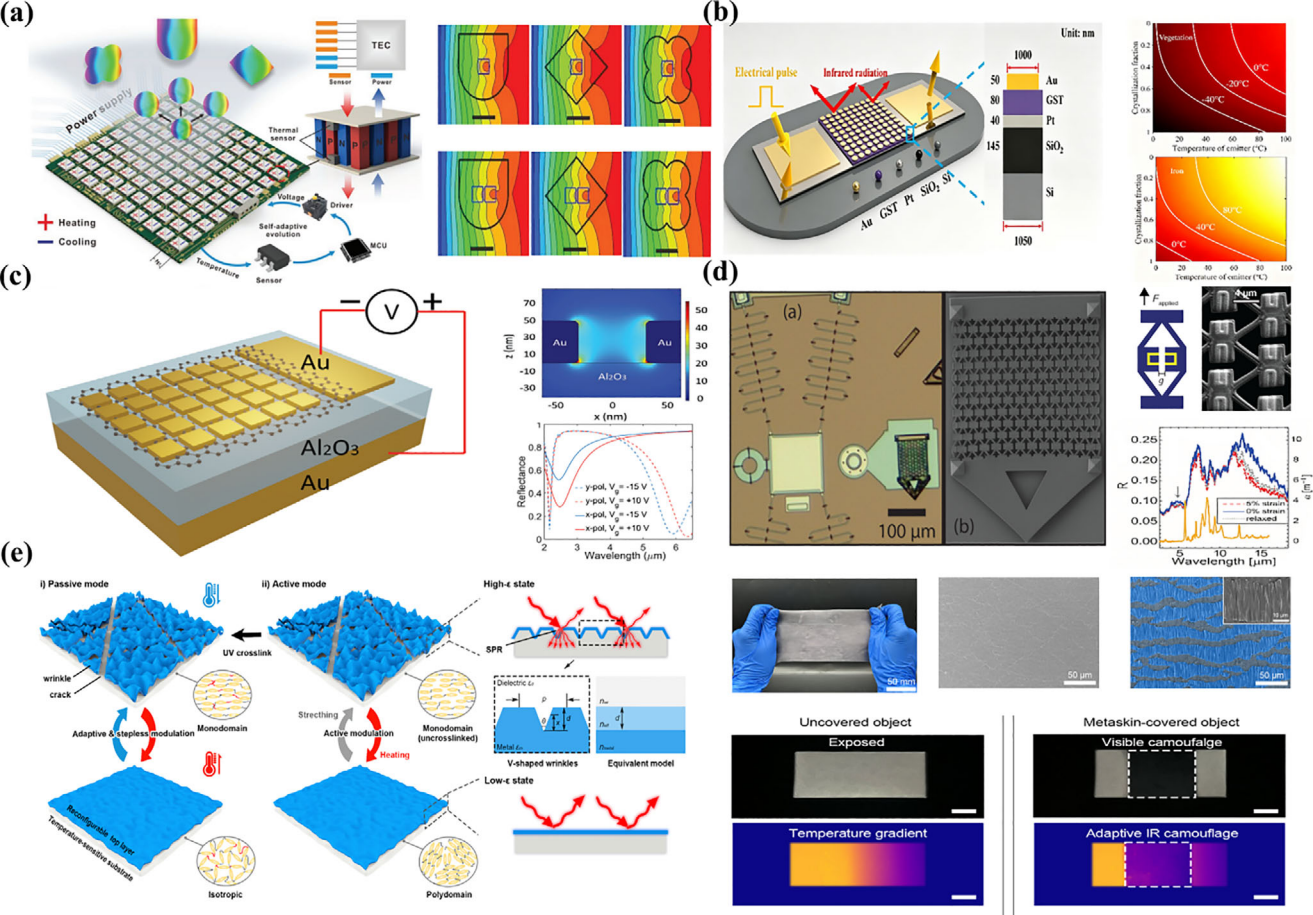
Thermal stealth metasurface. (a) PCB‐Integrated Thermoelectric Cooler Array Metasurface with Programmable Heat Conduction Modulation for Real‐Time Self‐Adaptive Thermal Camouflage. Reprinted under the terms and conditions of the Creative Commons Attribution (CC BY) license (https://creativecommons.org/licenses/by/4.0/) [94]. Copyright 2022 The Authors, published by Wiley‐VCH. (b) Electrically tunable GST‐based MIM metasurface for dynamic infrared thermal camouflage via nonvolatile emissivity switching. Reprinted under the terms and conditions of the Creative Commons Attribution (CC BY) license (https://creativecommons.org/licenses/by/4.0/) [103]. Copyright 2024 the authors. Published by Chinese Laser Press. (c) Electrically Tunable Hybrid Graphene‐Au Plasmonic Metasurface with Al_2_O_3_ Dielectric Layer for Near‐Infrared Reflection Modulation. Reprinted with permission [105]. Copyright 2022 Wiley‐VCH. (d) MEMS‐Enabled Al Asymmetric Split‐Ring Resonator Metasurface on DLW‐Printed IP‐Dip Polymer Scaffold for Strain‐Induced Mid‐Infrared Reflectivity Tuning. Reprinted with permission [107]. Copyright 2018 American Chemical Society. (e) MXene/LCE Bilayer Metasurface with Reconfigurable Wrinkle‐Crack Texture for Dual‐Mode Self‐Adaptive and Active Infrared Thermal Camouflage. Reprinted with permission [108]. Copyright 2025 Wiley‐VCH.

Effective thermal camouflage relies on precise tuning of heat conduction pathways, heat transfer rates and heat flux distribution to match the target's thermal signature with ambient backgrounds, thus reducing thermal contrast and thereby lowering detectability by infrared imagers. In particular, active thermal regulation based on thermoelectric cooling/heating devices enables real‐time absorption or release of heat flux to achieve dynamic thermal homogenization and camouflage. As a representative study, Guo et al. [94] fabricated thermal metasurfaces integrated with a 10 × 10 thermoelectric cooler (TEC) pixel array, as shown in Figure 11a. The system features a fast response time of 3–5 s and achieves pixel‐level heat flux control via real‐time feedback. On copper substrates with volumetric power density up to 2 × 10^7^ W/m^3^, the design realizes planar camouflage for rectangular and rhombic heat sources, homogenizing global temperature gradients and restricting thermal contrast below 0.5°C. This metasurface can eliminate surface hotspots generated by embedded heat sources and even generate programmable virtual thermal decoys to mislead infrared reconnaissance. Another technical route focuses on directional manipulation of thermal radiation. Siegel et al. developed electrically tunable graphene metasurfaces to dynamically steer the primary thermal radiation lobe in the mid‐infrared band under applied bias, supporting continuous angular scanning within ±16°. Thermal radiation energy is redirected toward non‐detection directions, drastically lowering the infrared signal intensity captured along target detection lines [95].

Emissivity regulation technology suppresses the intrinsic infrared radiation of substrates by adopting low‐emissivity media, aligning the thermal signatures of high‐temperature objects with surrounding environments. Infrared signals within atmospheric windows experience relatively low atmospheric attenuation, allowing them to propagate over long distances to infrared detectors. Therefore, infrared stealth materials are required to maintain low emissivity across these wavebands. Over the past decades, extensive research has been carried out on tunable infrared camouflage materials [96, 97, 98]. Dynamic emissivity adjustment can be realized by introducing stimuli‐responsive materials whose optical properties change under external excitation.

Phase‐change materials including VO_2_, In_3_SbTe_2_ (IST) and Ge_2_Sb_2_Te_5_ (GST) serve as core functional layers for thermal metasurfaces. Sharp refractive index shifts during phase transition enable wide‐range emissivity tuning, which has become a widely used dynamic‐modulation strategy. As a typical thermally triggered phase‐change medium, VO_2_ undergoes reversible insulator–metal transition near 68°C, and its dramatic refractive index variation greatly reshapes the resonant response of metasurface units. Xun et al. [99] designed scalable infrared switching metasurfaces based on VO_2–_Al heterostructure integration. The device consists of a CaF_2_ substrate, a 50 nm VO_2_ phase‐change film, and a 200 nm cross‐shaped Al array. It leverages the insulator–metal transition of VO_2_ and plasmonic extraordinary optical transmission to realize dynamic transmittance switching within the 8–14 µm atmospheric window, while blocking transmission in visible and near‐infrared bands. Kang et al. [100] proposed hybrid Si/VO_2_ meta‐atoms for mid‐infrared active metasurfaces. Embedding VO_2_ thin films at different positions on silicon nanodisks enables independent tuning of electric and magnetic dipole resonances, achieving transmittance modulation from 82% down to 28% at 4.6 µm. Beyond thermally driven VO_2_, non‐volatile phase‐change media such as IST and GST further expand the modulation dimension of radiative camouflage. Liu et al. [101] demonstrated pixel‐level electrically reconfigurable thermal metasurfaces using GeTe. The Fabry–Pérot resonant architecture comprises a GeTe top layer, dielectric spacer, and metallic back reflector. Benefiting from the large optical contrast between crystalline and amorphous GeTe states, the metasurface achieves wide‐range emissivity tuning in the 8–14 µm window. The non‐volatile phase transition maintains stable intermediate emissivity states without continuous power supply, and pixelated electrical addressing supports spatially resolved thermal encoding and programmable infrared camouflage with low power consumption and fast response. Wang et al. [102] designed multi‐band compatible laser camouflage films based on IST, adopting a multilayer thin‐film stacking structure of ZnS/Ge/MgF_2_/Ge/IST/W. Electrical pulse excitation triggers IST phase switching to realize reversible emissivity variation from 0.03 to 0.66 in the non‐atmospheric 5–8 µm band, while maintaining stable camouflage performance in visible, near‐infrared and laser bands (1.06 µm, 1.54 µm). Even under a 50°C temperature difference between target and background, the apparent thermal contrast captured by thermal imagers is controlled within 5°C. Xiong et al. [103] reported electrically controlled metal‐insulator‐metal (MIM) thermal emitters based on GST for broadband infrared modulation, as illustrated in Figure 11b. The MIM stack includes a platinum bottom conductive electrode, intermediate GST phase‐change layer, and top gold pillar array. External electrical pulses generate Joule heating to induce GST phase transition. Combined pulse amplitude and duration control, together with optimized thermal management, enables continuous adjustment of GST crystallinity from 0% to 100%. The prepared thermal emitter achieves dynamic emissivity tuning from 0.74 to 0.10 within 8–14 µm, matching the thermal radiation signatures of vegetation and metal backgrounds.

Electrically responsive materials adjust dielectric constants via carrier injection or Fermi level engineering to realize fast, precise regulation of emissivity, reflectivity and thermal radiation direction. Such devices feature ultra‐fast response and high tuning accuracy, where carrier mobility and intrinsic optical response dominate modulation performance. Chae [104] of Southern California fabricated metal‐semiconductor‐metal (MSM) tunable metasurfaces based on GaAs. Epitaxial transfer technology integrates III‐V thin films onto metal substrates. Forward bias triggers carrier injection, and the Moss–Burstein effect reduces the GaAs refractive index by 0.15, generating a spectral blue shift of 180 nm in the 6.4–8.7 µm mid‐infrared band. Cai et al. [105] designed electrically driven hybrid graphene metasurfaces operating in the near‐infrared regime, as shown in Figure 11c. Single‐layer graphene serves as the functional layer, and gold nanostructures act as plasmonic resonators. Hybrid integration of metal plasmonic structures with graphene strengthens interband transition efficiency for near‐infrared tuning. When gate voltage is applied across the Al_2_O_3_ dielectric spacer between graphene and the bottom Au backplane, carrier density in graphene is dynamically modulated to adjust reflectivity. Local field enhancement from gold nanoslits further amplifies graphene interband effects, delivering an electrically controlled reflectivity modulation depth of 17% at 2.42 µm, which is insensitive to carrier mobility fluctuations.

Deformable material‐based metasurfaces reconfigure thermal radiation channels and resonant modes via mechanical deformation, supporting high‐contrast switching between absorption and emission states. Their overall performance is determined by the combination of intrinsic optical properties and structural deformability, with large modulation range and excellent long‐term stability. Liu et al. [106] proposed reconfigurable mid‐infrared radiative metasurfaces (MME) with suspended Au‐Ge‐Au functional films on alumina substrates. Electrostatic actuation controls contact/separation between suspended membranes and the substrate. At constant ambient temperature, applied voltage generates electrostatic force to reshape the unit geometry, realizing absorption modulation ranging from 0.2 to 0.95 at 8.9 µm. Reeves et al. [107] developed tunable infrared metasurfaces supported by flexible polymer scaffolds, as depicted in Figure 11d. The structure integrates IP‐Dip photoresist honeycomb substrates and aluminum resonant rings. The high Poisson's ratio of IP‐Dip converts uniaxial tensile strain into lateral gap variation of meta‐atoms. Tuning the spacing of split‐ring resonators modulates near‐field coupling and resonant wavelengths, producing a maximum red shift of 1 µm under mechanical strain, while reflectivity drops from 80% to 40%. Chen et al. [108] designed plasmonic MXene‐liquid crystal elastomer composite adaptive metasurfaces, as shown in Figure 11e. Synergistic control of surface plasmon resonance and thermally induced LCE deformation enables dual‐mode passive/active infrared camouflage. In passive self‐adaptive mode, the film spontaneously changes from wrinkled plasmonic textures to flat layers with ambient temperature fluctuation, achieving continuous emissivity adjustment up to 41% in the 8–14 µm band with a 1.2 s response speed. In active programmable mode, external thermal or mechanical stimulation switches and locks high/low emissivity states without continuous energy input, realizing long‐term energy‐free thermal camouflage.

To provide an intuitive comparison of different technical approaches for thermal camouflage metasurfaces, Table 2 summarizes the tuning categories, core operating bands, and tunable infrared emissivity performance of representative works, illustrating the adaptive infrared camouflage capabilities across different mechanisms.

**TABLE 2:** Comparison of Reported Thermal Camouflage Metasurfaces.

Refs.	Technical Approach	Microstructural Design	Tuning Category	Core Band (µm)	Tunable Infrared Emissivity Performance
[94]	Thermal Conduction Cloaking	10 × 10 TEC pixel array / Cu substrate	Active thermal regulation	3–14	ΔT < 0.5°C

[95]	PCM	Graphene / Au slit array / SiN_x_ FP cavity	Thermal emission beam steering	6.61	Steering ±16°
[99]		VO_2_(50 nm) / Al cross‐slot array / CaF_2_	Transmittance switching	8–14	ΔT = 0.367 (0.367→0) @10.64 µm
[100]		Si / VO_2_ hybrid nanodisks / CaF_2_	Transmittance modulation	3–5	ΔT = 0.54 (0.82→0.28) @4.6µm
[102]		ZnS/Ge/MgF_2_/Ge/IST/W multilayer	Emissivity modulation	5–8	Δε = 0.63 (0.03→0.66) @5–8 µm
[103]		Pt/GST/Au pillar MIM stack	Emissivity modulation	8–14	Δε = 0.74 (0.74→0) @10.6 µm

[104]	Electrically Responsive Material	GaAs p‐i‐n / Au grating / Au substrate MSM	Wavelength shift	6.4–8.7	Δλ = 180 nm
[105]		Au nanoslit / graphene / Al_2_O_3_ / Au	Reflectance modulation	2.42	ΔR = 0.099 (0.582→0.483) @2.42µm

[106]	Deformable Material	Au‐Ge‐Au cantilever / Al_2_O_3_ substrate	Absorption modulation	8.9	ΔA = 0.75 (0.2→0.95) @8.9µm
[107]		Al ASRR / IP‐Dip honeycomb scaffold	Wavelength shift	7–20	Δλ = 1µm

#### 3.2.3 Optical Metasurfaces

The core goal of visible‐light stealth is to reduce optical detectability of targets within the visible spectrum. Traditional pigment‐based camouflage relies on static passive color matching and struggles to adapt to complex, time‐varying battlefield environments. In contrast, metasurfaces leverage subwavelength nanostructures to precisely manipulate light scattering, reflection, phase and polarization, realizing structural‐color camouflage based on fundamental optical physical effects. Its performance breakthrough stems from synergistic optimization between the optical response of functional media and customized micro‐nano architectures. Fundamentally, rational material selection and structural design achieve seamless visual integration with surrounding backgrounds, and even enable real‐time adaptation to dynamic environments, thereby reduce contrast against optical backgrounds and accomplishing effective concealment.

##### 3.2.3.1 Metallic Plasmonic Metasurfaces

Plasmonic structural‐color metasurfaces mainly utilize surface plasmon resonance (SPR) generated on metallic nanostructures to selectively scatter or absorb light at designated wavelengths, producing vivid structural hues. Surface plasmons are hybrid EM modes formed by coupling collective free‐electron oscillations and optical fields at metal‐dielectric interfaces. Such modes tightly confine optical energy to subwavelength interfacial regions and induce intense local electric field enhancement. Resonance occurs when incident light frequency matches the intrinsic oscillation frequency of free electrons inside metal nanostructures, leading to strong selective absorption or scattering at target wavebands.

Careful tuning of metal nano‐units, including geometry, size, material composition and lattice period, enables precise control over resonant peak position and spectral linewidth, generating arbitrary structural colors covering visible to infrared bands. Nanodisks, nanorods and nanorings fabricated from noble metals (gold, silver) exhibit highly geometry‐sensitive SPR responses. Increasing nanodisk diameter triggers pronounced spectral red shifts of scattering peaks; adjusting the aspect ratio of nanorods modulates resonance under different polarization directions. Such high tunability lays the foundation for generating diversified camouflage colors. For stealth applications, a prominent advantage of plasmonic metasurfaces lies in their ultrathin thickness (tens to hundreds of nanometers), which greatly facilitates lightweight integration and system assembly. Kim et al. [31] proposed metal‐semiconductor‐metal (MSM) metasurfaces formed by integrating aluminum nanodisk arrays with multilayer Ge/Ag films. Localized surface plasmon resonance (LSPR) of aluminum nanodisks shapes visible reflection spectra to generate camouflage color patterns; meanwhile, the structure maintains strong absorption in near‐infrared and short‐wave infrared bands, with mid‐long‐wave infrared emissivity suppressed below 0.05 to weaken thermal radiation signatures. Combined LSPR, Fabry–Pérot resonance and gap plasmon effects integrate visible camouflage, laser stealth and thermal camouflage into a single passive thin film, providing a multispectral compatible solution for unmanned equipment. Nevertheless, plasmonic metasurfaces have inherent drawbacks. First, high‐performance plasmonic units rely on expensive noble metals (Au, Ag), which suffer poor chemical stability; long‐term air exposure causes oxidation and sulfurization, leading to gradual color degradation. Second, intrinsic Joule loss of metals broadens resonant linewidths, limiting the saturation of generated structural colors.

##### 3.2.3.2 Silicon‐Based All‐Dielectric Metasurfaces

**FIGURE 12:**
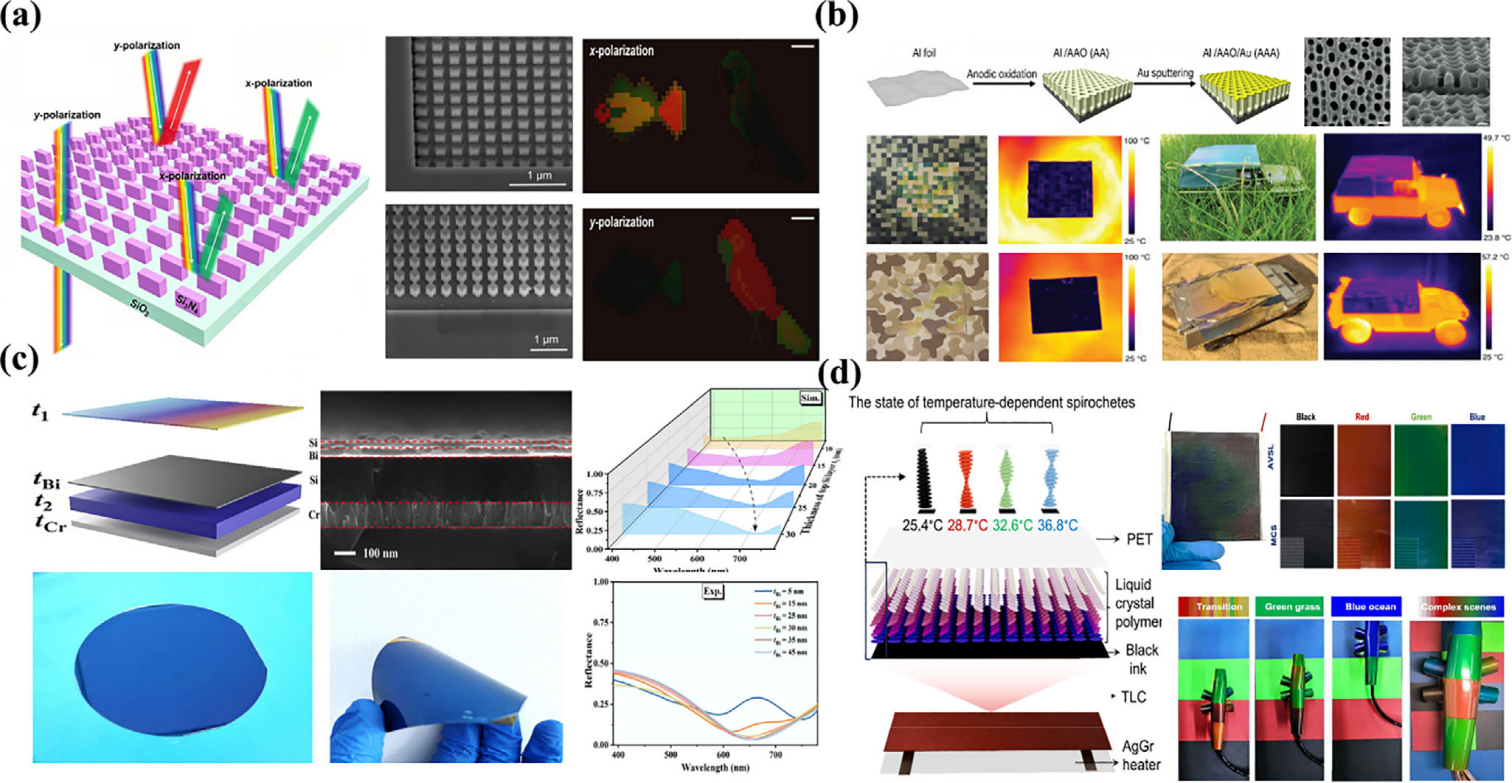
Visual camouflage metasurface. (a) Polarization‐Switchable All‐Dielectric Metasurface Composed of Si_3_N_4_ Mie‐Resonant Nanorod/Nanocross Arrays on Quartz Substrate for Full‐Color Printing and Optical Encryption. Reprinted with permission [110]. Copyright 2023 American Chemical Society. (b) Semi‐Open Fabry–Pérot Plasmonic Metasurface Based on Au‐Anodic Aluminum Oxide‐Aluminum (AAA) Multilayer Structure for Scalable Flexible Visible‐Infrared Compatible Camouflage. Reprinted under the terms and conditions of the Creative Commons Attribution (CC BY) license (https://creativecommons.org/licenses/by/4.0/) [111]. Copyright 2023 the authors. Published by Wiley‐VCH. (c) Deep‐Subwavelength Si/Bi/Si/Cr Multilayer Metacoating for Visible‐Infrared Camouflage. Reprinted under the terms and conditions of the Creative Commons Attribution (CC BY) license (https://creativecommons.org/licenses/by/4.0/) [113]. Copyright 2024 the authors. Published by John Wiley and Sons. (d) ITO/AgGr/TLC Multilayer Metasurface for Multispectral Camouflage. Reprinted with permission [115]. Copyright 2024 Elsevier.

All‐dielectric metasurfaces constructed from high‐refractive‐index, low‐loss media such as silicon, silicon nitride and titanium dioxide have become promising optical camouflage platforms, featuring excellent structural compatibility, chemical durability and mechanical strength. Their optical modulation capacity originates from material optical anisotropy and polarization/angle‐dependent responses of microstructures, supporting polarization‐switchable and angle‐adaptive color generation without extra active components. Furthermore, all‐dielectric materials possess outstanding thermal and chemical stability, guaranteeing long‐term service reliability of structural‐color devices under harsh conditions. Significant progress has been made in all‐dielectric structural‐color metasurfaces. Huang et al. [109] designed a biomimetic perturbation‐based optical metasurface consisting of a 15 nm silicon nanodisk perturbation layer stacked on a 305 nm silicon substrate. Guided by optical perturbation theory, the structure effectively suppresses unwanted modal resonance and accurately replicates the broadband transmission and phase characteristics of bulk silicon substrates. This transforms a pistol‐shaped target into a cat‐like optical profile to realize active optical deception without strict background matching, breaking the narrow bandwidth and limited observation angle bottlenecks of traditional camouflage. The device maintains stable performance over an ultra‐wide 138° observation angle and a 160 nm spectral bandwidth. Wang et al. [110] fabricated all‐dielectric metasurfaces based on silicon nitride and fused silica, realizing fully polarization‐switchable high‐saturation structural colors, as shown in Figure 12a. The film displays bright chromatic tones under long‐axis polarized light and uniform black under orthogonal polarization. Further optimized Si_3_N_4_ nano‐cross arrays generate three distinct display states, with reflection FWHM narrowed to 34–40 nm and a color gamut covering 90% of the standard sRGB range, offering a novel material platform for dynamic display and optical encryption. However, silicon all‐dielectric structural‐color devices still face practical obstacles. Unlike metals, dielectric nanostructures lack strong local field confinement, requiring larger geometric sizes to sustain sufficient resonance strength and restricting further ultrathinning. Besides, large‐area uniform color fabrication demands ultrahigh nanofabrication precision; tiny dimensional deviations cause resonant shifts and uneven color rendering.

##### 3.2.3.3 Multilayer Heterogeneous Metasurfaces

Multilayer heterogeneous thin films stack dielectric and plasmonic metal layers with complementary optical functions. Metallic layers deliver high reflectivity, low infrared emissivity and plasmonic modulation capability; dielectric interlayers enable precise tuning of visible structural colors. Accurate control of each layer's thickness optimizes optical interference effects to achieve improved spectral matching with backgrounds. Multilayer stacking also serves as a universal framework to integrate multiple optical mechanisms and is widely adopted for multispectral compatible camouflage. Xiong et al. [111] reported flexible plasmonic thin films based on semi‐open Fabry–Pérot cavities for visible‐infrared dual‐band camouflage, as illustrated in Figure 12b. The Au‐AA‐Al metal‐dielectric‐metal (MDM) architecture adopts porous anodic aluminum oxide (AAO) as the dielectric spacer. Tuning AAO thickness and surface metal coverage simultaneously regulates structural color and infrared emissivity. The design achieves minimal chromatic aberration with desert backgrounds in the visible band, while suppressing emissivity to roughly (0.17) (3–5 µm) and (0.143) (8–14 µm) in atmospheric windows. Wang et al. [112] constructed SiO_2_/Cu/SiO_2_/Pt multilayer heterostructures for integrated visible, infrared and laser camouflage. Through the combined effects of cavity resonance, destructive interference, and bimetallic defects, adjusting the thickness of SiO_2_ spacers modulates structural hues, while the top silica layer enables efficient laser absorption. The composite supports wide‐gamut color rendering, low thermal emissivity and weak laser reflection, suitable for multi‐mode detection camouflage scenarios. Tan et al. [113] proposed an ultrathin four‐layer deep‐subwavelength coating (Si/Bi/Si/Cr) with total thickness merely 355 nm, as shown in Figure 12c. Bismuth exhibits metallic optical properties in the visible band and lossy dielectric characteristics in infrared regions; visible hues are independently regulated by the top silicon layer thickness. The film maintains high emissivity within non‐atmospheric windows for radiative cooling and low emissivity across infrared atmospheric windows, covering a broad color range from purple to red. Infrared thermal tests show a radiation temperature reduction of 7.6°C compared with reference samples at 110°C. The coating retains reliable camouflage performance on flexible substrates and effectively weakens thermal signatures when attached to human skin, greatly expanding environmental adaptability.

##### 3.2.3.4 Active Tunable Optical Metasurfaces

Realizing adaptive camouflage requires structural‐color metasurfaces to possess dynamically adjustable optical responses, shifting from static color rendering to real‐time environment‐matching switching. Tunable metasurfaces integrate active functional media or micro‐electro‐mechanical systems (MEMS) into nanoarchitectures. External electrical, thermal or optical stimuli alter resonant conditions to actively control color, reflectivity and infrared emissivity. Such dynamic modulation remains a cutting‐edge research direction toward practical intelligent camouflage. Electrical tuning is one of the most straightforward, integrable strategies, where electric fields or currents change the intrinsic optical constants of materials and reshape light‐matter interactions. Liquid crystals are typical tunable dielectrics; voltage‐induced molecular reorientation modulates effective refractive index with millisecond response and low power consumption. Badloe et al. [114] fabricated electrically controlled Mie‐resonator metasurfaces integrated with liquid crystals. Applied voltage continuously adjusts the effective polarization of incident light and precisely tunes the resonance intensity of anisotropic silicon nanopillars, supporting smooth pixel transition from saturated color to near‐black states. The design achieves millisecond‐scale electrical modulation without extra polarization optical elements, simplifying system integration. Via voltage programming, identical metasurface patterns can hide encrypted information under bright colors or expose it against black backgrounds, providing high‐resolution, high‐contrast solutions for dynamic display and optical encryption. Zhang et al. [115] developed bio‐inspired multi‐band adaptive camouflage films relying on synergistic thermochromic liquid crystals, silver nanowire‐graphene composite and ITO layers, as depicted in Figure 12d. Thermochromic liquid crystals realize full‐spectrum color switching from black to blue via helical molecular rearrangement. Highly conductive Ag nanowire‐graphene films enable efficient electrothermal driving with a response speed as fast as 0.73 s and temperature control accuracy better than 0.3°C, achieving dynamic visible camouflage. Meanwhile, the ITO layer maintains an infrared emissivity of 0.24, and the silver nanowire‐graphene metasurface attains over 90% absorption efficiency within 10–37.5 GHz. The composite retains stable performance after 500 bending cycles, realizing integrated visible, infrared and radar adaptive camouflage. In addition to liquid crystal systems, phase‐change materials show distinct refractive index and extinction coefficient differences between amorphous and crystalline states. Embedding phase‐change thin films into metasurfaces enables dynamic adjustment of reflection/transmission color and absorption by switching crystalline states. Ruiz de Galarreta et al. [32] designed hybrid dielectric‐phase‐change metasurfaces for dynamic visible structural‐color tuning. The structure uses TiO_2_ nanopillars as Mie resonators embedded with low‐loss Sb_2_S_3_ phase‐change thin films. Reversible amorphous‐crystalline phase transition actively modulates Mie resonance. In the amorphous state, strong electric dipole resonance at 520 nm generates green structural color with conversion efficiency up to 60%. Thermal, optical or electrical pulses trigger crystallization to suppress resonance and fade the film to near white, realizing non‐volatile color on/off switching. Selectively embedding phase‐change media at electric field antinodes maintains high quality factors while retaining dynamic tunability, offering a new high‐efficiency route for visible‐band non‐volatile dynamic display and anti‐counterfeiting devices.

#### 3.2.4 Acoustic Metasurfaces

Acoustic stealth represents a cutting‐edge research field that manipulates sound propagation, scattering and reflection to reduce target acoustic detectability, with broad application prospects in military concealment, underwater surveillance, noise suppression and medical ultrasound imaging. Traditional acoustic media such as sound‐absorbing foams [116, 117] and rubber composites [118] suffer inherent drawbacks: their effective working bands are limited to narrow frequency ranges, which restricts full‐spectrum acoustic control under complex service environments.

As two‐dimensional artificial metamaterials constructed from periodic subwavelength microstructures, acoustic metasurfaces feature ultrathin thickness, light weight and flexible wavefront shaping capacity, and have become a core technical route for high‐performance acoustic stealth.

Transformation acoustics constitutes the fundamental design principle for acoustic stealth metasurfaces, originating from transformation optics in photonics [119]. Through mathematical coordinate transformation, this theory straightens the propagation trajectories of acoustic waves in inhomogeneous and anisotropic virtual space and maps them into physical space, thereby guiding the design of metamaterials with designated distributions of equivalent physical parameters. For a given coordinate transformation *x*′ =  *f*(*x*), where *x* and *x*′ denote the coordinates in physical and virtual space, respectively, specific relationships exist between the isotropic medium parameters in virtual space (mass density ρ′ and bulk modulus *K*′) and the equivalent anisotropic parameters in physical space ρ_*eff*_ and *K_eff_*. Assuming the virtual space is free space (ρ′ = ρ_0_ , *K*′ = *K*_0_ ), the equivalent parameters in physical space can be formulated as:

(16) $$\rho_{eff}=\rho_{0}\;\frac{F^{-1}F^{-T}}{det\left(F\right)},\;{K_{eff}}^{-1}=\frac{{K_{0}}^{-1}}{det\left(F\right)}\;$$

where $F=\frac{\partial x^{\prime }}{\partial x}$ is the Jacobian matrix of the coordinate transformation, det(F) denotes its determinant, and F^‐T^ represents the transpose of the inverse Jacobian matrix. Equation (16) reveals that acoustic cloaking requires metamaterials in physical space to possess spatially varying anisotropic effective density and bulk modulus distributions. However, fabricating bulk metamaterials with such complex parameter gradients poses substantial fabrication challenges. Acoustic metasurfaces provide a feasible simplified pathway: they reduce complex bulk parameter modulation to two‐dimensional planar phase modulation based on the generalized Snell's law. When acoustic waves impinge on a metasurface, each subwavelength unit imposes a predetermined phase shift on the incident wave. Rational layout of unit geometry and spatial arrangement generates arbitrary phase gradients on the planar interface, enabling precise directional steering of reflected or transmitted acoustic waves.

##### 3.2.4.1 Space‐Folding Acoustic Metasurfaces

**FIGURE 13:**
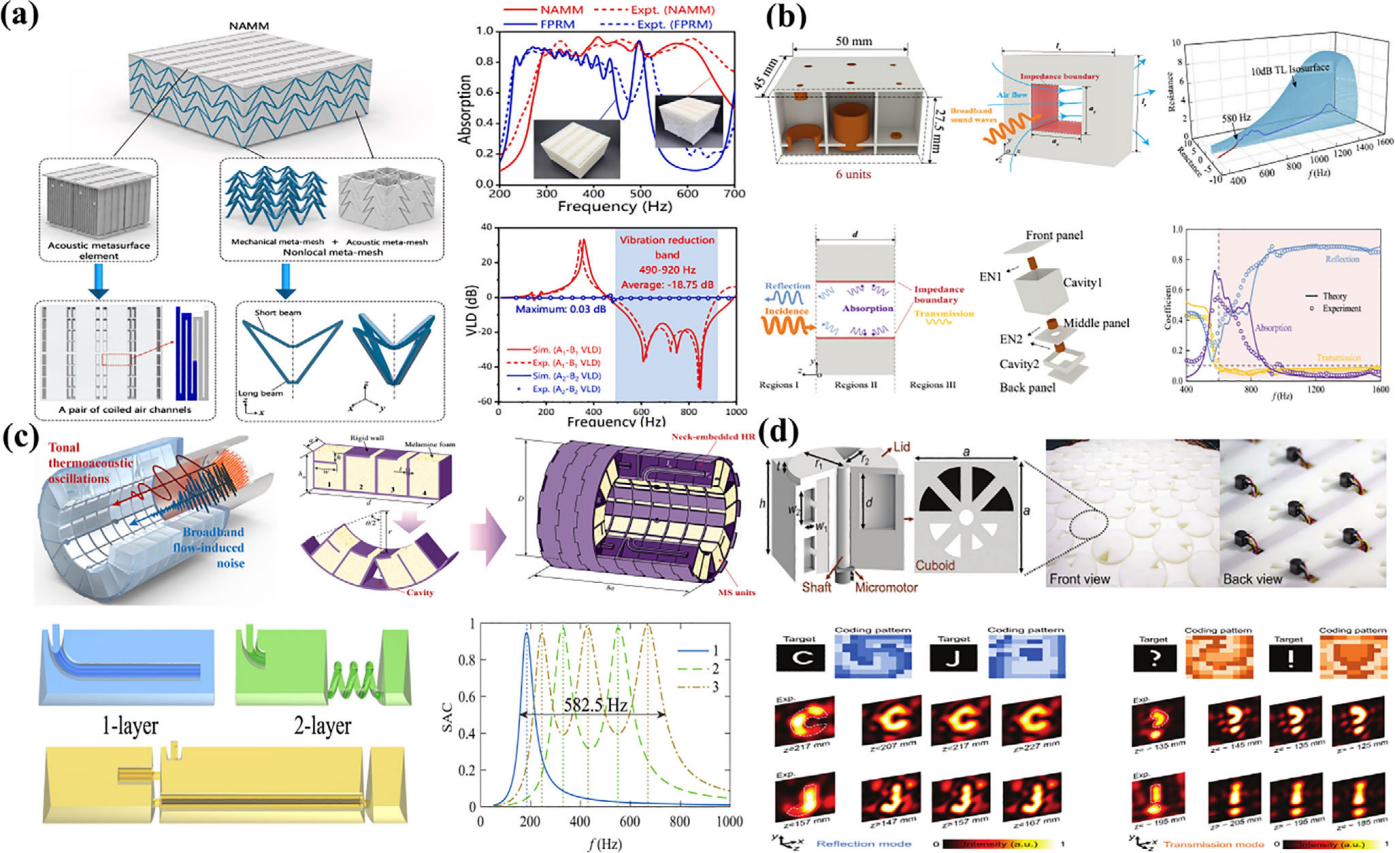
Acoustic stealth metasurface. (a) Space‐Folded Nonlocal Acoustic‐Mechanical Metasurface for Broadband Sound Absorption and Vibration Reduction. Reprinted under the terms and conditions of the Creative Commons Attribution (CC BY) license (https://creativecommons.org/licenses/by/4.0/) [120]. Copyright 2024 the authors. Published by Elsevier Ltd. (b) Nonlocal Metasurface with Coupled Helmholtz Resonators for Broadband Sound Absorption. Reprinted with permission [123]. Copyright 2025 Elsevier. (c) Space‐Folded Nonlocal Acoustic Metasurface with Embedded Helmholtz Resonators for Broadband Noise Reduction. Reprinted with permission [125]. Copyright 2025 Elsevier. (d) Motor‐Driven Programmable Acoustic Metasurface with 2‐Bit Coding for Full‐Space Sound Manipulation. Reprinted with permission [126]. Copyright 2024 Wiley‐VCH.

Space‐folding metasurfaces extend sound propagation paths via curled, labyrinth or multi‐fold channels within ultra‐thin planar layers to realize energy dissipation or phase accumulation at deep subwavelength scales. The core advantage lies in ultra‐thin profiles and excellent low‐frequency broadband absorption performance. Guo et al. [34] proposed ultrathin absorbers consisting of spiral neck resonators and curled back cavities. Spiral folded structures substantially lengthen acoustic trajectories and strengthen viscous dissipation, achieving an absorption coefficient up to 98% at 180 Hz with a total thickness of merely 13 mm (approximately λ/145, far thinner than conventional space‐folding absorbers). To broaden bandwidth, multiple resonators with distinct geometric dimensions are arranged in parallel sharing a single front panel. Multi‐resonance coupling generates dual‐band and broadband absorption, whose spectral response can be tuned by adjusting unit size. The structure maintains stable absorption under 30° and 45° oblique incidence. Furthermore, folded channel frameworks support multifunctional integration. Zeng et al. [120] designed nonlocal acousto‐mechanical composite metasurfaces, as illustrated in Figure 13a. Nonlocal meta‐lattices composed of 3D printed frames, porous media and double‐arrow units are arranged at acousto‐mechanical interfaces. Inter‐unit nonlocal coupling simultaneously optimizes sound absorption and vibration suppression while retaining lightweight characteristics. The composite delivers an average absorption coefficient over 0.9 within 267–638 Hz (58.6% performance improvement), and achieves vibration attenuation of 18.75 dB with a damping efficiency of 88.45% across 490–920 Hz.

Space‐folding acoustic metasurfaces enable ultrathin subwavelength acoustic modulation and can be readily fabricated for low‐frequency indoor acoustic regulation. Their practical performance is largely limited by intrinsic structural defects. Labyrinthine channels induce obvious viscous and thermal losses at high frequencies, reducing acoustic efficiency. The resonance‐based working principle also yields a narrow effective bandwidth. Accordingly, these structures are viable mainly for low‐frequency and confined‐space scenarios, with limited applicability for broadband and high‐intensity acoustic applications.

##### 3.2.4.2 Local Resonance Acoustic Metasurfaces

Local resonance metasurfaces integrate subwavelength resonant units (Helmholtz resonators, elastic oscillators, thin‐film membranes) to modulate acoustic scattering and absorption via strong frequency‐selective resonant responses, compensating scattering distortion induced by concealed targets. Representative configurations include membrane resonators and Helmholtz resonator (HR) arrays. Thin‐film metasurfaces realize efficient absorption at deep subwavelength sizes with light weight and tunable responses via tension or electrical bias [121, 122]. Nevertheless, low‐frequency resonance requires large membrane mass or low pretension, which compromises structural compactness and limits practical applicability. The Helmholtz resonator represents the most classical configuration for resonant acoustic metasurfaces. Its resonant frequency *f*_0_ is determined by the neck cross‐sectional area S, neck length L, cavity volume V and end correction δ, following $f_{0}=\frac{c}{2\pi }\;\sqrt{\frac{S}{V(L+\delta )}}$. Tuning these geometric parameters enables the design of meta‐atoms with tailored phase responses at designated frequencies. Based on this principle, Jia et al. [123] constructed nonlocal HR array metasurfaces for broadband ventilated sound barriers, as shown in Figure 13b. Strong inter‐unit coupling suppresses anti‐resonance dips and overcomes the narrowband limitation of single resonators. Based on mode matching theory, the metasurface realizes sound insulation efficiency above 90% within 600–1600 Hz while retaining a 20% open ventilation area, with total thickness only 50 mm. Assembling multi‐frequency HR supercells further expands absorption bandwidth for acoustic stealth applications. Ryoo et al. [124] fabricated thin multi‐resonance absorbing metasurfaces. Each supercell contains two adjacent HR subunits. A π phase difference generates destructive interference and near‐field wave trapping to lower the quality factor, achieving perfect single‐frequency absorption. Optimizing cavity and neck dimensions realizes multi‐frequency hybrid resonance. The absorber thickness is below λ/11, with average absorption above 0.8 over 395–815 Hz and stable performance at incident angles from 0° to 60°. Most existing research focuses on low‐frequency ultrathin absorbers. Targeting thermoacoustic high‐frequency noise, Zheng et al. [125] designed ultrathin metallic lining integrating gradient porous metasurfaces and embedded HRs, as depicted in Figure 13c. The metasurface converts incident sound into surface waves to enhance high‐frequency dissipation, while HR units strongly attenuate low‐frequency thermoacoustic tones. Rijke tube tests verify that the 2 cm thick lining reduces broadband high‐frequency noise by up to 36 dB and suppresses discrete thermoacoustic oscillations by 124.9 dB, maintaining stability under variable flow velocity and heating power.

Local resonance acoustic metasurfaces achieve effective subwavelength absorption at tunable low frequencies. Their performance is constrained by narrow working bandwidth and angle‐dependent acoustic response. Structural nonlinearity may degrade absorption performance under high sound pressure. Large‐scale fabrication also faces stability issues. These metasurfaces suit scenarios for targeted low‐frequency noise reduction, but are not applicable to broadband noise environments.

##### 3.2.4.3 Phase‐Gradient & Coding Acoustic Metasurfaces

Coding metasurfaces treat each subwavelength unit as an independent acoustic logic bit. Arranging units with discrete phase shifts in predefined sequences utilizes destructive interference to drastically reduce target scattering cross‐section (SCS), featuring wide bandwidth and adaptability to curved surfaces. Zhang et al. [126] proposed motor‐programmable reconfigurable acoustic metasurfaces, as shown in Figure 13d. Each cell consists of a fixed cuboid cavity and rotatable cover containing eight embedded resonators (four reflection channels, four transmission channels). Motor‐driven rotation achieves 2‐bit phase modulation, with near‐unity reflection amplitude and transmission amplitude above 0.75. A 10 × 10 unit array supports real‐time coding switching and dynamic acoustic focusing at 4000 Hz for both reflection and transmission modes. For underwater acoustic stealth, Yu et al. [127] designed broadband 2‐bit coded metasurfaces fabricated by perforating steel panels. Four‐hole coupling generates anti‐resonance effects, stably maintaining a π/2 phase interval between four coding units. Genetic algorithm optimization arranges unit distribution to suppress total scattering via wave interference. The structure achieves SCS reduction below −10 dB over 14.3–40.5 kHz with a fractional bandwidth of 95.62%, valid for incident angles 0° to 60° and curved targets. Compared with traditional carpet cloaks, it delivers wider bandwidth and simpler manufacturing for moving underwater platforms. Nevertheless, coding metasurfaces adopt discrete phase states, introducing step approximation errors during wavefront reconstruction; functional accuracy is limited by the number of phase discretization levels. Different from discrete coding designs, Fan et al. [128] reported continuously reconfigurable curved acoustic metasurfaces. Each tunable cell combines spiral channels and matching screws; rotating the screw adjusts spiral depth to realize full continuous 2π phase coverage within 2–7 kHz. Thirty‐one units are arranged on an arc substrate, whose phase distribution is engineered following the generalized Snell's law to switch between two core functions: acoustic carpet cloaking (restoring planar reflected wavefronts to hide obstacles) and terrain illusion (simulating arbitrary convex/concave surface reflection signatures). Experiments verify stable cloaking and illusion performance for normal incidence and 30° to 60° oblique incidence at 2.2 and 6.1 kHz. The design can be extended to 3D hemispherical targets, offering broadband tunable solutions for underwater acoustic camouflage and target concealment.

Phase‐gradient and coding acoustic metasurfaces manipulate acoustic wavefronts via discrete phase‐controlled unit cells, providing flexible directional modulation and programmable acoustic functions. Their practical performance is restricted by inherent technical constraints. Discrete phase coding tends to produce quantization errors and side lobes, while frequency‐dependent phase dispersion hinders broadband operation. High‐efficiency full‐phase modulation also requires precise impedance matching. Such metasurfaces are feasible for directional acoustic detection and communication, yet their performance is limited by fabrication accuracy and frequency adaptability.

### 3.3 Development Trends of Next‐Generation Electromagnetic Low‐Detectability Metasurfaces

#### 3.3.1 Multispectra‐Compatible Dynamically Reconfigurable Stealth Technology

With the rapid development of multi‐band integrated detection technologies, stealth schemes relying on single‐spectrum and static responses are insufficient for multi‐dimensional detection threats in complex operational environments. Multispectral compatibility and dynamic reconfigurability have thus become the core development directions of next‐generation low‐detectability technologies. Among the various spectral bands involved, the radar‐infrared compatibility challenge is the most critical and extensively studied bottleneck. This is because radar stealth and infrared stealth impose fundamentally conflicting requirements on the absorptivity of stealth materials. According to Kirchhoff's law of thermal radiation, infrared stealth requires low emissivity, which corresponds to low absorptivity; radar stealth, on the other hand, requires strong absorption of incident EM waves to minimize backscattering, demanding high absorptivity. This inherent contradiction in absorptivity constitutes the fundamental scientific difficulty in achieving radar‐infrared compatible stealth. More broadly, similar physical conflicts exist among other spectral bands—for example, visible‐light camouflage requires specific color and reflection properties that may interfere with infrared emissivity control or microwave absorption, and laser stealth demands selective absorption or reflection at discrete wavelengths that may not align with broadband radar or infrared requirements. Therefore, the core challenge of multispectral compatible stealth lies in reconciling these conflicting EM requirements across disparate frequency bands within a single material system.

Traditional static approaches attempt to address this challenge through two general strategies. The first strategy is to develop single‐layer materials that simultaneously fulfill multiple spectral requirements. A representative advancement is the single‐layer ITO chaotic coding metasurface by Wang et al. [129] which, through a chaotic paradigm combined with a multiscale strategy, simultaneously achieves >10 dB RCS reduction within 8–18 GHz (up to 45° incidence), infrared emissivity below 0.3, and 71.2% optical transmittance within an ultralight 3.35 mm single layer, demonstrating that even a passive single‐layer architecture can partially reconcile the compatibility conflict between radar and infrared functions through deterministic pseudo‐random coding and multiscale structural synergy. The second strategy is to composite multiple functional materials, each responsible for a specific band, while minimizing mutual interference. A representative advancement is the flexible multilayer metasurface by Wang et al. [130] which adopts a three‐functional‐layer architecture—optical camouflage layer (OCL), infrared functional layer (IRFL), and microwave functional layer (MFL)—and achieves, within a single device of less than 3 mm total thickness, simultaneous visible‐light color matching, >90% absorption of the 1.06 µm laser, infrared emissivity below 0.2 across the 2–14 µm band, and >90% microwave absorption in the X‐band (8–12 GHz), showcasing the integration capability of the multilayer composite route for multispectral compatibility. However, these static approaches can only achieve a compromised balance at the design stage; once fabricated, they cannot adapt to changing detection environments or dynamically switch between different operational modes. Consequently, there is growing interest in dynamically tunable multispectral stealth. Benefiting from their flexible EM wave manipulation capabilities, metasurfaces have emerged as promising platforms for multispectral‐compatible dynamic stealth. Through innovative material‐structure integrated designs—such as transparent conductive films, frequency‐selective surfaces (FSSs), phase‐change materials, and shared functional layers—metasurface‐based multispectral stealth systems can manage competing requirements among different spectral bands, enabling synchronous or independent, collaborative, and interference‐free dynamic regulation across two or more spectral regimes. This ultimately leads to intelligent, self‐adaptive stealth architectures capable of countering sophisticated integrated detection systems.

##### 3.3.1.1 Single‐Band Dynamically Reconfigurable Stealth Metasurfaces

**FIGURE 14:**
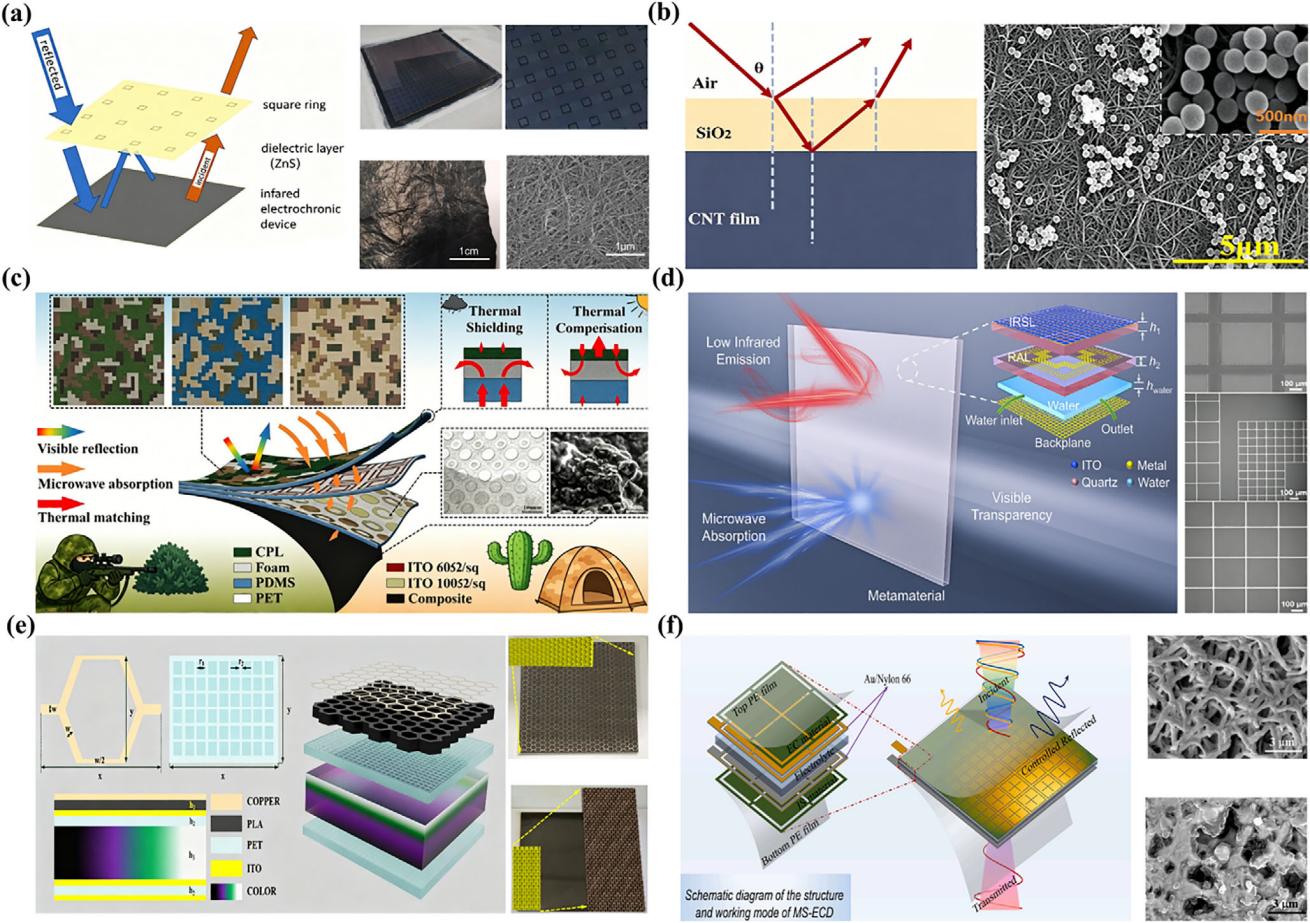
(a) ITO/ZnS/CNT Multilayer Metasurface for Radar Absorption and Dynamic Infrared Stealth. Reprinted with permission [131]. Copyright 2024 Elsevier. Published by Elsevier Ltd. (b) SiO_2_/ZnO‐CNT Composite Film for Visible Structural Color and Dynamic Infrared Modulation. Reprinted with permission [132]. Copyright 2025 Elsevier. (c) PDMS/ITO‐Based Fabry–Pérot Metasurface with Dynamic Visible/Infrared Modulation and Broadband Radar Absorption. Reprinted with permission [133]. Copyright 2025 Wiley‐VCH. (d) ITO/Au Mesh/Water Multilayer Metasurface for Dynamic Radar Absorption and Infrared Stealth. Reprinted with permission [35]. Copyright 2025 Wiley‐VCH. (e) Mechanically Reconfigurable Honeycomb Metasurface for Dynamic Radar Absorption, Thermochromic Visible Camouflage, and Static Infrared Stealth. Reprinted with permission [137]. Copyright 2025 Wiley‐VCH. (f) Polyaniline‐Based Electrochromic Metasurface with Frequency‐Selective Surface for Dynamic Visible/Infrared Modulation and Microwave Transmission. Reprinted with permission [138]. Copyright 2024 Elsevier.

This category of metasurfaces adopts one core responsive material targeted at a dominant detection band to realize precise dynamic tuning under external stimuli. For other detection bands including infrared, microwave, and laser, stable static multispectral compatibility is maintained by virtue of inherent properties of complementary static materials and structural optimization. Their prominent merits include concise material composition, clear modulation mechanisms, controllable power consumption and low engineering implementation cost. For dynamic infrared stealth, Qu et al. [131] proposed a composite structure integrating carbon nanotube (CNT) film‐based electrochromic infrared dynamic stealth devices and indium tin oxide–zinc sulfide (ITO‐ZnS) radar‐absorbing metamaterials, as shown in Figure 14a. The single‐layer CNT film serves simultaneously as the electrode of the electrochromic infrared device and the reflective backplane of the metamaterial. The design achieves broadband and high‐efficiency radar absorption while enabling reversible dynamic regulation of long‐wave infrared emissivity from 0.323 to 0.768 within the 8–14 µm atmospheric window, accompanied by over 90% microwave absorption across 9.67–16.04 GHz. Aiming at the limitation that the intrinsic strong visible‐light absorption of CNT films restricts their application in multispectral camouflage, Liu et al. [132] proposed two parallel structural coloration strategies, as illustrated in Figure 14b. Silica photonic crystals synthesized via the Stöber method and zinc oxide thin films prepared by magnetron sputtering endow CNT films with visible structural colors such as blue, yellow, and purple through photonic bandgap engineering and thin‐film interference effects, respectively, without relying on light‐colored substrates. Meanwhile, the inherent infrared emissivity modulation capability of CNT films up to 0.358 is well preserved. To address spectral crosstalk and insufficient environmental adaptability in existing multispectral camouflage systems, Yang et al. [133] developed a Fabry–Pérot matched‐loss metasurface that synergistically integrates adaptive visible–infrared camouflage and ultra‐broadband microwave absorption, as shown in Figure 14c. A reconfigurable camouflage pixel layer is constructed atop the Fabry–Pérot cavity to adaptively generate pixelated patterns matching woodland, ocean, and mountain backgrounds. The integrated system precisely regulates thermal radiation signatures, matching a 36°C target with gravel surroundings and restricting the thermal contrast below 3.2°C. Coupling the Fabry–Pérot cavity with double‐layer patterned ITO absorbers further realizes ultra‐broadband microwave absorption spanning 5.7–73.2 GHz. Furthermore, Meng et al. [35] reported a metamaterial‐inspired infrared electrochromic device. A reversible metal electrodeposition infrared electrochromic structure and an ITO‐based FSS are integrated onto a shared barium fluoride substrate. The monolithic device achieves reversible broadband emissivity modulation in the infrared regime and broadband microwave absorption within 8.5–18 GHz, eliminating the strong radar reflection defect induced by conventional electrodes in infrared electrochromic systems. Dynamic radar stealth focuses on real‐time reversible switching and tuning of microwave reflection and absorption while maintaining inherent static camouflage performance in infrared and visible bands. Lan et al. [134] designed a transparent tunable dual‐band stealth metamaterial based on water substrates and metallic meshes, as shown in Figure 14d. The top layer consists of ITO square patch arrays acting as an infrared shielding layer; the middle layer serves as a microwave absorber integrated with ultrathin gold meshes of varied periods; the bottom layer adopts a water substrate and metallic mesh reflective backplane. By adjusting the water layer thickness, the effective absorption bandwidth exceeding 90% can be switched between an ultra‐broadband mode of 3.6–15.8 GHz and dual narrowband modes of 3.6–5.9 GHz and 11.2–13.8 GHz, while maintaining a low infrared emissivity of approximately 0.37 and an average visible transmittance of 65.5%. Zhang et al. [36] proposed a water‐switchable metamaterial for radar‐infrared compatible stealth. The top ITO patch layer achieves low infrared emissivity of approximately 0.388 in the 3 to 14 µm band. By injecting or draining water in PDMS microfluidic channels, the structure switches between broadband absorption exceeding 90% from 12.44 to 28.72 GHz and broadband reflection across 10 to 30 GHz. Meanwhile, Liu et al. [135] proposed a multispectral compatible programmable coding metasurface with a thickness of approximately 0.1λ_0_. By integrating PIN diodes, the meta‐atoms achieve continuous amplitude dynamic modulation and 1‑bit phase dynamic modulation, enabling independent dynamic control of absorption intensity and scattering direction across a broad bandwidth. Simultaneously, owing to the surface ITO structures and copper mesh design, the PCM exhibits an average infrared emissivity of about 0.25 in the 3–14 µm band and an optical transmittance of 65.9% at 565 nm, demonstrating the potential of electronically controlled pathways for dynamic radar‑band stealth. For protective modulation against specific‐wavelength laser detection, Jiang et al. [136] developed a laser‐adaptive inverse‐designed metamaterial via the synergy of phase‐change materials and inverse‐engineered photonic structures. Utilizing the phase‐transition characteristics of VO_2_ or Sb_2_S_3_, the device adaptively switches within approximately 1 s from a high‐absorption camouflage mode for 1.06 µm, 1.55 µm and 10.6 µm laser incidence to a high‐reflection optical limiting mode for laser protection, while preserving continuous visible and infrared stealth performance throughout the transition process.

##### 3.3.1.2 Multi‐Band Dynamically Tunable Stealth Metasurfaces

Metasurfaces of this type integrate multiple responsive materials to construct material systems capable of cross‐band synergistic regulation. Through monolithic integrated design, they enable simultaneous or independent dynamic performance manipulation over two or more spectral bands, representing an active research direction of contemporary multispectral stealth technology. Wang et al. [137] presented a mechanically tunable honeycomb‐structured metamaterial, as shown in Figure 14e. The architecture consists of a deformable mechanical layer fabricated via 3D‐printed polylactic acid (PLA) composites, an infrared stealth layer composed of ITO thin films on PET substrates, and a color‐switching layer incorporating PDMS blended with organic thermochromic microcapsules. Mechanical compression reconfigures the honeycomb geometry to dynamically switch microwave absorption peaks between 7.66 GHz and 16.51 GHz and realize broadband absorption across 30–100 GHz. The thermochromic layer enables reversible color evolution from black, purple, and green to off‐white within 27–35°C, while the ITO layer maintains an infrared emissivity below 0.292. Ultimately, tunable microwave absorption, dynamic visible camouflage and high‐efficiency infrared stealth are integrated within a single flexible platform. Zhang et al. [138] reported a multispectral‐compatible electrochromic device based on polyaniline and triphenylamine‐modified polyaniline copolymers, as depicted in Figure 14f. The core innovation introduces the FSS concept into electrode design, constructing multifunctional electrodes with periodic subwavelength metallic mesh patterns via laser etching. The device enables reversible visible color switching between jungle dark green and desert khaki, delivers ultra‐wide infrared emissivity modulation amplitudes of 0.61 and 0.73 within atmospheric windows, and achieves efficient microwave transmission across 10.2–13.9 GHz by virtue of the bandpass filtering characteristics of the patterned electrode.

Phase‐change material‐based devices extend multispectral dynamic regulation to independent and synergistic management of optical and thermal radiation. Zeng et al. [139] proposed a reconfigurable hyperspectral camouflage metasurface based on Sb_2_S_3_ micropore structures. Using the refractive index contrast between amorphous and crystalline states, the device mimics green and brown vegetation spectra (SAM = 0.83 for both). It simultaneously achieves low mid‐infrared emissivity (ε_3__−__5 _µm = 0.2, ε_8__−__14 _µm = 0.33) and high infrared emissivity (ε_2_._5__−__3 _µm = 0.76, ε_5__−__8 _µm = 0.52) to balance stealth and passive radiative cooling. It also absorbs strongly at 1550 nm for LiDAR suppression. Laser‐induced Sb_2_S_3_ phase transitions enable reversible, spatially programmable optical state tuning. Yuan et al. [140] proposed a GST‐based responsive phase‐gradient metasurface. It uses a “0‐1‐2‐1‐0‐1‐2” coding sequence to create a multi‐level phase gradient across 3–18 µm. This achieves angle‐dependent directional reflection with a specular reflectance of about 0.2. By exploiting the GST phase transition, the device attains dynamic thermal emissivity modulations of 0.32 in 3–5 µm and 0.64 in 7.5–14 µm. It can switch between low‐ and high‐radiation‐temperature states. Directional reflection control and emissivity modulation operate independently without mutual coupling.

Collectively, these studies demonstrate that the synergy between active components (PIN diodes, varactors) and responsive materials (phase‐change materials, liquid crystals) is a key enabler for next‐generation multispectral adaptive stealth, making it possible to achieve independent spatial and spectral control within a unified, manufacturable platform.

To provide an intuitive comparison of different technical approaches for radar‐infrared compatible stealth metasurfaces, Table 3 summarizes both radar and infrared performance of representative works, revealing the current design landscape and the trade‐offs between radar and infrared tunability.

**TABLE 3:** Comparison of Reported Radar‐Infrared Compatible Stealth Metasurfaces.

Refs.	Microstructure Design	Technical Approach	IR Tun.	Radar Tun.	Radar Performance	Tunable IR Emissivity Performance
[131]	CNT / ITO‐ZnS square‐ring metamaterial	Electrochromism	√	×	Absorption: 9.67–16.04 GHz	Emissivity tunable: 0.323→0.768 (Δε = 0.445)
[35]	Cu RME / ITO Jerusalem‐cross FSS / BaF_2_		√	×	Absorption: 8.5–18 GHz	Emissivity tunable: Δε = 0.57 (3–5 µm); Δε = 0.48 (7.5–13 µm)
[138]	Au mesh / TPA‐PANI / nylon		√	√	Switchable peak: 10.2 GHz → 13.9 GHz (Δf = 2.7 GHz)	Emissivity tunable: Δε = 0.61 (3–5 µm); Δε = 0.73 (8‐14 µm)

[133]	Thermochromic pixel / ITO‐composite F‐P absorber	Thermochromic	√	×	Absorption: 5.7–73.2 GHz	Thermochromic regulation, thermal contrast <3.2°C

[134]	ITO patch / Au mesh / water substrate	Water switching	×	√	Switchable mode: Broadband (3.6–15.8 GHz) → Dual‐band (3.6–5.9 & 11.2–13.8 GHz)	ε≈0.37(8–14 µm)
[36]	ITO patch / PDMS microchannel / ITO‐PET		×	√	Switchable mode: Absorption (12.44–28.72 GHz) → Reflection (10–30 GHz)	ε≈0.39(3–14 µm)

[135]	ITO patch / Cu mesh with PIN diodes / PC‐PET	PIN diode	×	√	Switchable amplitude: ΔA = 0.65 (0.85→0.20)	ε≈0.25(3–14 µm)

[137]	Doped PLA honeycomb / PDMS+thermochromic / ITO‐PET	Mechanical compression	×	√	Switchable peak: 7.66 GHz → 16.51 GHz	ε = 0.292(3–14 µm)

#### 3.3.2 Multifunctionally Integrated Stealth Metasurfaces

With the progressive deployment of stealth technologies in advanced scenarios including aerospace systems, intelligent equipment, and wearable devices, single‐function stealth designs can no longer meet practical engineering requirements. Modern combat platforms are required not only to evade multispectral detection, but also to integrate comprehensive performances such as thermal management, structural durability, and environmental adaptability. Accordingly, multifunctional integration has become an emerging trend for advanced metasurface stealth technology. The core design philosophy abandons the traditional separated layout of functional devices and load‐bearing structural components. Through integrated structure–function co‐design, metasurfaces achieve low observability while embedding auxiliary capabilities including radiative cooling, mechanical load bearing, and flexible conformal adaptation, substantially improving environmental resilience, mechanical reliability, and engineering practicability. Benefiting from inherent two‐dimensional planar characteristics, metasurfaces provide a versatile platform for multifunctional integration. Precise modulation of micro‐nano geometric configurations, material combinations, and spatial arrangements enables a single metasurface to synergistically respond to EM, thermal, and mechanical multiphysics fields. This strategy realizes collaborative optimization of stealth and auxiliary functionalities, while avoiding redundant volume, additional weight, and poor compatibility caused by discrete component stacking.

##### 3.3.2.1 Radiative Cooling

**FIGURE 15:**
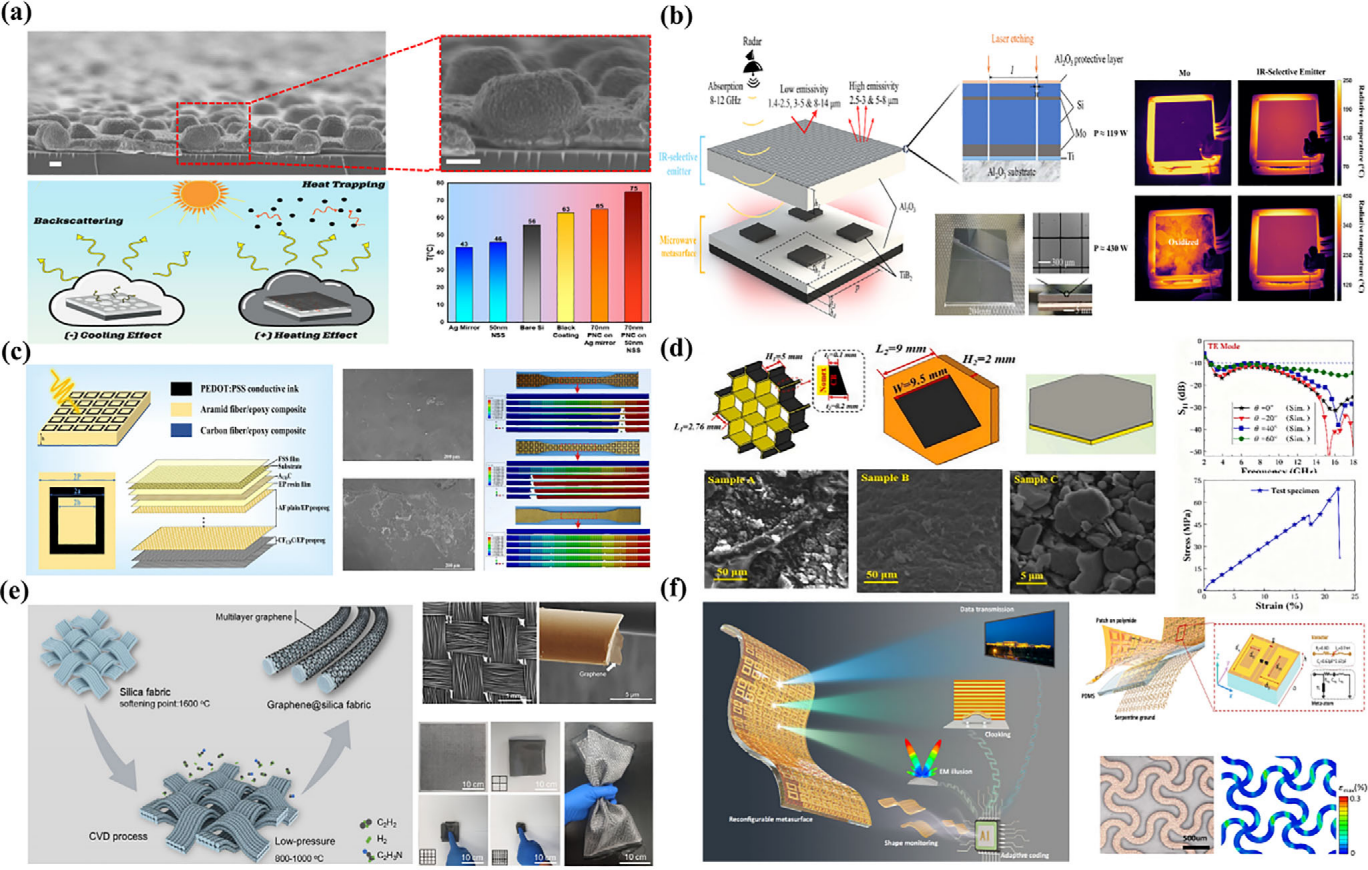
Multifunctionally integrated stealth metasurface. (a) Cloud‐inspired plasmonic metasurfaces integrating visible camouflage with radiative cooling. Reprinted under the terms and conditions of the Creative Commons Attribution (CC BY) license (https://creativecommons.org/licenses/by/4.0/) [143]. Copyright 2025 the authors. Published by Wiley‐VCH. (b) Integrated IR‐selective emitter and microwave metasurface for high‐temperature multi‐infrared and microwave stealth with radiative cooling. Reprinted under the terms and conditions of the Creative Commons Attribution (CC BY) license (https://creativecommons.org/licenses/by/4.0/) [144]. Copyright 2025 the authors. Published by Springer Nature. (c) Metasurface microwave‐absorbing composite integrated with high mechanical load‐bearing capacity. Reprinted with permission [145]. Copyright 2025 Springer Nature. (d) Low‐profile broadband microwave‐absorbing composite metasurface based on magnetic coating and artificial EM structures Reprinted with permission [147]. Copyright 2023 Elsevier. (e) Flexible Graphene@Silica Fabric Metasurface. Reprinted with permission [151]. Copyright 2025 Wiley‐VCH. (f) Flexible intelligent microwave metasurface with shape‐guided adaptive programming. Reprinted with permission [157]. Copyright 2025 Springer Nature.

Passive daytime radiative cooling (PDRC) has emerged as a vital research direction for metasurface‐based thermal management [141, 142]. Its fundamental principle relies on precise spectral engineering: the surface possesses ultrahigh infrared emissivity within atmospheric transparent windows to efficiently dissipate terrestrial heat to cold outer space via thermal radiation, while maintaining high reflectivity across the solar spectrum to minimize solar heat absorption. Such surfaces achieve sub‐ambient temperature cooling under direct sunlight with zero external energy consumption. Owing to their exceptional spectral tailoring capability, metasurfaces are promising candidates for high‐performance PDRC systems. Diversified radiative cooling metasurfaces have been developed based on dielectric media, photonic crystals, multilayer thin‐film interference, and metal–dielectric resonant meta‐atoms. Inspired by natural cloud–aerosol interactive mechanisms, Assad et al. [143] proposed white and grey plasmonic metasurfaces for thermally adaptive camouflage and thermal management, as shown in Figure 15a. The white plasmonic configuration mimics cloud cooling effects via strong backward scattering, reducing the substrate temperature by 10°C under standard solar irradiation. In contrast, the grey composite structure suppresses backward scattering and enhances optical trapping, producing a 10°C higher temperature than conventional black absorbers. Both configurations exhibit ultra‐low infrared emissivities of 0.11 and 0.08, respectively, integrating efficient radiative cooling and superior infrared thermal camouflage. To address the heat dissipation bottleneck of stealth materials under extreme high‐temperature conditions, Zhao et al. [144] developed a multispectral‐compatible stealth and thermal management device via monolithic integration of infrared selective emitters and microwave metasurfaces, as illustrated in Figure 15b. The infrared selective emitter adopts an alumina/silicon/molybdenum multilayer stack, which maintains low emissivity within short‐wave, mid‐wave, and long‐wave infrared atmospheric windows to suppress thermal signatures, while achieving high emissivity in the 5–8 µm non‐atmospheric window for efficient radiative heat dissipation. The underlying microwave metasurface consists of periodic titanium diboride arrays, alumina dielectric spacers, and titanium diboride backplates, realizing microwave reflection loss below −3 dB across the entire X‐band. Under an operating temperature of 700°C and aerodynamic thermal load equivalent to Mach 2.2 flight, the proposed structure achieves a maximum temperature reduction of 72.4°C compared with conventional low‐emissivity molybdenum substrates, simultaneously satisfying multispectral infrared stealth, microwave camouflage, and high‐temperature thermal regulation requirements. These advances demonstrate the great potential of metasurfaces for high‐efficiency, tunable PDRC and stealth integration systems.

##### 3.3.2.2 Structural Load Bearing

Traditionally, metasurfaces are regarded as planar EM modulation components, with research focus primarily placed on optimizing subwavelength‐unit‐based EM responses. Most existing studies prioritize EM performance while neglecting mechanical properties, especially structural load‐bearing capacity. Nevertheless, advanced aerospace vehicles, mobile robots, and wearable electronics impose stringent demands on comprehensive mechanical performances, including structural strength, stiffness, toughness, and lightweight load‐bearing capability, to withstand complex dynamic loads such as vibration, impact, tension, compression, and bending. In this context, load‐bearing multifunctional metasurfaces that integrate EM/acoustic stealth functions with structural mechanical performance have become an emerging and challenging research frontier. A typical strategy is to adopt high‐mechanical‐performance materials as metasurface substrates or structural units, including high‐strength engineering polymers, aramid fabrics, carbon fiber composites, and metallic foils. Yang et al. [145] fabricated microwave‐absorbing metasurface patterns on unidirectional aramid fabrics using high‐performance poly (3,4‐ethylenedioxythiophene): poly (4‐styrenesulfonic acid sodium) (PEDOT: PSS) conductive ink, achieving integrated microwave absorption and structural load‐bearing functions, as shown in Figure 15c. The composite delivers broadband microwave absorption with an effective bandwidth of 8.9 GHz at a thin thickness of merely 3.2 mm. Benefiting from excellent interfacial compatibility between aramid fabrics and epoxy matrices, the structure exhibits superior mechanical robustness, with substantially improved tensile strength, interlaminar shear strength, and impact resistance compared with polyimide‐based counterparts. This work verifies that rational substrate selection and fabrication optimization can endow metasurfaces with prominent load‐bearing capacity without sacrificing EM stealth performance. Beyond material optimization, elaborate meta‐unit geometry design and global topological layout provide another effective route for balanced stealth and mechanical performance. Embedding stealth metasurfaces into panel cores or filling absorbing media into cellular frameworks enables simultaneous structural support and EM modulation. As a typical demonstration, Jiang et al. [146] constructed integrated microwave absorption–transmission sandwich structures based on composite corrugated channels. The core framework is fabricated from fiberglass‐reinforced epoxy composites, with gradient‐length fishbone‐shaped metallic patterns decorated on outer surfaces and polymethacrylimide foam filled in internal cavities. Based on spatial dispersion engineering and EM resonance regulation, the structure achieves over 80% absorption within 5.74–7.84 GHz and over 80% transmission across 8.96–12.45 GHz. Mechanical tests verify that embedded foam significantly enhances compression resistance with a peak compressive strength of 25.21 MPa, outperforming conventional metallic sandwich structures and realizing integration of EM functionality and mechanical durability. Furthermore, Ren et al. [147] reported broadband composite microwave absorbers with integrated structural and stealth performances, as shown in Figure 15d. Resistive frequency selective surface (FSS) layers are embedded in polypropylene foam panels, carbon‐based absorbing fillers are patterned within Nomex honeycomb cavities, and carbonyl iron powder magnetic loss coatings are deposited on metallic backplates. The three‐dimensional synergistic design of EM architectures and functional media achieves broadband absorption exceeding 90% throughout 2.06–18 GHz at a thickness of 8.3 mm, accompanied by excellent mechanical stability with a maximum uniaxial tensile strength of 67.43 MPa.

##### 3.3.2.3 Flexible Conformal Adaptation

In recent years, stretchable, morphing, and conformal metasurfaces have attracted increasing attention as emerging research directions in flexible deformable stealth. For instance, Fan et al. [148] demonstrated a skin‐like stretchable metasurface for mechanically tunable terahertz modulation, and Han et al. [149] developed magnetic origami‐inspired flexible metastructures capable of large deformation at cryogenic temperatures. Among them, conformal metasurfaces have made particularly notable progress due to their close relevance to practical engineering requirements such as flexible substrates and surface conformability. The construction of conformal stealth metasurfaces relies on high‐quality flexible material systems, including flexible functional media such as graphene and metallized fabrics, as well as compliant supporting substrates. Polymeric materials such as polyimide, polydimethylsiloxane (PDMS), and polyethylene terephthalate (PET) are widely adopted as flexible substrates due to their superior flexibility, thermal stability, chemical inertness, and facile processability. Synergistic optimization of intrinsic material properties and structural design guarantees stable EM performance under curved attachment and dynamic mechanical deformation. Shi et al. [38] developed optically transparent, stealth‐compatible flexible metasurfaces based on high‐transparency, mechanically compliant PET substrates for integrated infrared and radar camouflage. Textile‐based substrates also exhibit inherent flexible advantages. Li et al. [150] proposed fabric‐based flexible EM absorbing metamaterials with native textile conformability. Fully flexible multilayer structures are constructed, where cobalt–nickel alloy‐coated carbon nanotubes and PEDOT:PSS composite layers provide synergistic magnetic and dielectric loss, while periodic metasurface patterns are fabricated on flexible aramid fabrics via laser‐induced graphene technology. The absorber achieves a minimum reflection loss of −26.4 dB and an effective absorption bandwidth of 8.0 GHz within 2–18 GHz, combining excellent radar stealth performance and reliable mechanical deformability. Targeting flexible and thermally stable absorbing materials for high‐speed aircraft, Cui et al. [151] designed flexible graphene–silica fabric metasurfaces via selective laser ablation, as shown in Figure 15e. Graphene layers are directly grown on silica fiber fabrics through chemical vapor deposition and further patterned via laser etching to regulate sheet resistance distribution. Such all‐inorganic architectures integrate ultra‐thin thickness, lightweight feature, superior flexibility, and exceptional high‐temperature resistance, enabling seamless conformal attachment to complex curved surfaces of high‐speed vehicles. Integrated with thermal insulation layers without introducing extra structural burden, the design achieves efficient EM absorption across 2–18 GHz with a minimum reflection loss of −42 dB, effectively suppressing the radar scattering cross‐section of aerial targets. Li et al. [152] further extended the multiscale design strategy of flexible conformal metasurfaces. Inspired by the hierarchical structure of skin, they combined directional freeze‐drying with screen printing to construct a multiscale metamaterial absorber (SMMA) consisting of flexible SiC@polyurethane aerogel synergized with a double‐layer resonant metasurface. It achieves an effective absorption bandwidth of 9.27 GHz and a peak reflection loss of –44.3 dB in the 2–18 GHz range. The vertical pore channels of the aerogel simultaneously provide excellent thermal insulation capability (reducing the surface temperature by nearly 48°C relative to a 100°C heat source), and the sample can conform to cylindrical and other complex curved surfaces. An et al. [153] prepared a flame‐retardant, thermally insulating, low‐dielectric, and superhydrophobic SiO_2_ fiber paper (SFP) via a papermaking process combined with surface modification, and subsequently constructed a high‐temperature‐resistant flexible two‐dimensional stealth metasurface by high‐precision screen printing. At a thickness of 400 µm, it achieves better than –8 dB absorption in the 10.9–16.4 GHz range; after 20 cycles of rolling, the absorption performance remains largely stable, and the backside temperature is reduced by 182°C compared with an alcohol lamp flame. Chen et al. [154] adopted a scalable femtosecond laser etching–transfer hybrid manufacturing strategy to construct MgF_2_/Ag/MgF_2_ hyperbolic metamaterial protrusion structures on a PDMS flexible substrate. This design simultaneously integrates three functions: high visible transparency (44%), low near‐infrared to infrared specular reflectance (R_s_,_1–2 _µm = 0.02, R_s_,_3–14 _µm = 0.10), and strong scattering in the lidar band (R_s_,_1_._06 _µm = 0.01). After 200 bending cycles, the emissivity remains stable around 0.2, and the device can conformally attach to complex curved surfaces such as vehicles and human hands. Liu et al. [155] integrated a VO_2_ phase‐change thin film with a gold nanobrick array on a mica flexible substrate. By current‐induced insulator‐to‐metal transition of VO_2_, they achieved reversible electrical switching between a half‐wave plate and a mirror in the infrared band, with polarization conversion efficiency remaining stable over a bending radius range of 9–19 mm, demonstrating flexible conformal infrared image steganography and dynamic camouflage.

In practical service scenarios including fluttering aircraft wings and soft robotic actuators, flexible conformal metasurfaces inevitably undergo unpredictable geometric distortion, which may cause severe EM performance degradation even for intrinsically flexible designs. Therefore, maintaining functional stability under dynamic mechanical deformation has become a core research challenge in conformal stealth technology. Nam et al. [156] proposed particle swarm optimization‐empowered flexible camouflage metasurfaces. Complementary split‐ring resonator‐patterned aluminum films are deposited on flexible polyimide substrates. Precise modulation of metallic layer thickness achieves an infrared emissivity modulation amplitude of 77.7% in the long‐wave infrared band, while maintaining a high microwave transmittance of 93.8% within the X‐band. The intrinsic flexibility of polyimide substrates and ultrathin metallic films guarantees superior bendability, tear resistance, and conformal attachability on complex curved surfaces. Moreover, geometry‐optimized single‐layer resonant layouts exhibit excellent mechanical robustness, retaining 89.8% infrared camouflage efficiency and stable microwave selective transmission under large bending deformation. A more advanced solution lies in intelligent closed‐loop adaptive regulation, which enables real‐time shape perception and active EM performance compensation against deformation. Li et al. [157] proposed a flexible intelligent metasurface platform consisting of three core modules: reconfigurable flexible metasurfaces, geometric morphology acquisition units, and bias voltage regulation systems, as shown in Figure 15f. Symmetric square‐ring resonators integrated with parallel varactor diodes constitute the reconfigurable meta‐array, enabling precise reflection phase modulation via external biasing, while serpentine interconnect layouts ensure intrinsic mechanical compliance. Back‐attached flexible sensor arrays acquire real‐time curvature distribution to monitor global morphological deformation. Embedded artificial neural network algorithms instantly process morphological data and output optimal bias voltage distribution to compensate deformation‐induced performance drift. The closed‐loop system achieves a rapid response speed of 16.7 ms, with excellent stability validated in EM illusion, carpet cloaking, and wireless communication applications. This pioneering work integrates shape sensing, intelligent algorithms, and reconfigurable metasurfaces to construct self‐adaptive closed‐loop systems, helping address the performance instability problem of flexible metasurfaces under dynamic deformation. It marks a critical technological transition from passive mechanical conformity to active intelligent conformal regulation.

Despite the substantial progress in flexible conformal stealth integration, multiple challenges remain. First, the EM loss characteristics, thermal endurance, long‐term durability, and environmental stability of flexible conductive and dielectric materials under harsh conditions (high humidity, salt fog, extreme temperature) require further systematic optimization. Second, scalable, high‐precision, and low‐cost manufacturing techniques for flexible metasurfaces, especially active reconfigurable architectures, still need further improvement. Third, theoretical modeling and numerical prediction of EM responses for highly stretchable and sharply bent metasurfaces remain computationally intensive, demanding more efficient design and simulation methodologies. Finally, practical engineering deployment imposes strict constraints on power consumption, system integration level, operational reliability, and control latency of intelligent adaptive stealth platforms.

#### 3.3.3 Data‐Driven Intelligent Stealth Metasurfaces

Conventional design and optimization of metasurfaces face substantial inherent challenges. Highly nonlinear and intricate correlations exist between geometric parameters, material compositions, and resultant EM responses, rendering traditional strategies heavily reliant on empirical experience and intuition. Such workflows inevitably involve numerous time‐consuming full‐wave EM simulations and trial‐and‐error iterations. For intelligent stealth scenarios imposing stringent requirements including multifunctional integration, broadband operation, wide‐angle performance, and dynamic tunability, the enormous computational overhead and prolonged design cycles severely restrict practical deployment. Against this backdrop, data‐driven methodologies, particularly machine learning and artificial intelligence, have opened up diverse innovative avenues for the rational design, systematic optimization, and real‐time control of intelligent stealth metasurfaces. These techniques not only improve design efficiency and overall device performance but also endow metasurface systems with enable adaptive capabilities, enabling environmental perception, autonomous decision‐making, and adaptive response to dynamic and complex stealth demands.

##### 3.3.3.1 Neural Network‐Based Metasurface Design and Optimization

**FIGURE 16:**
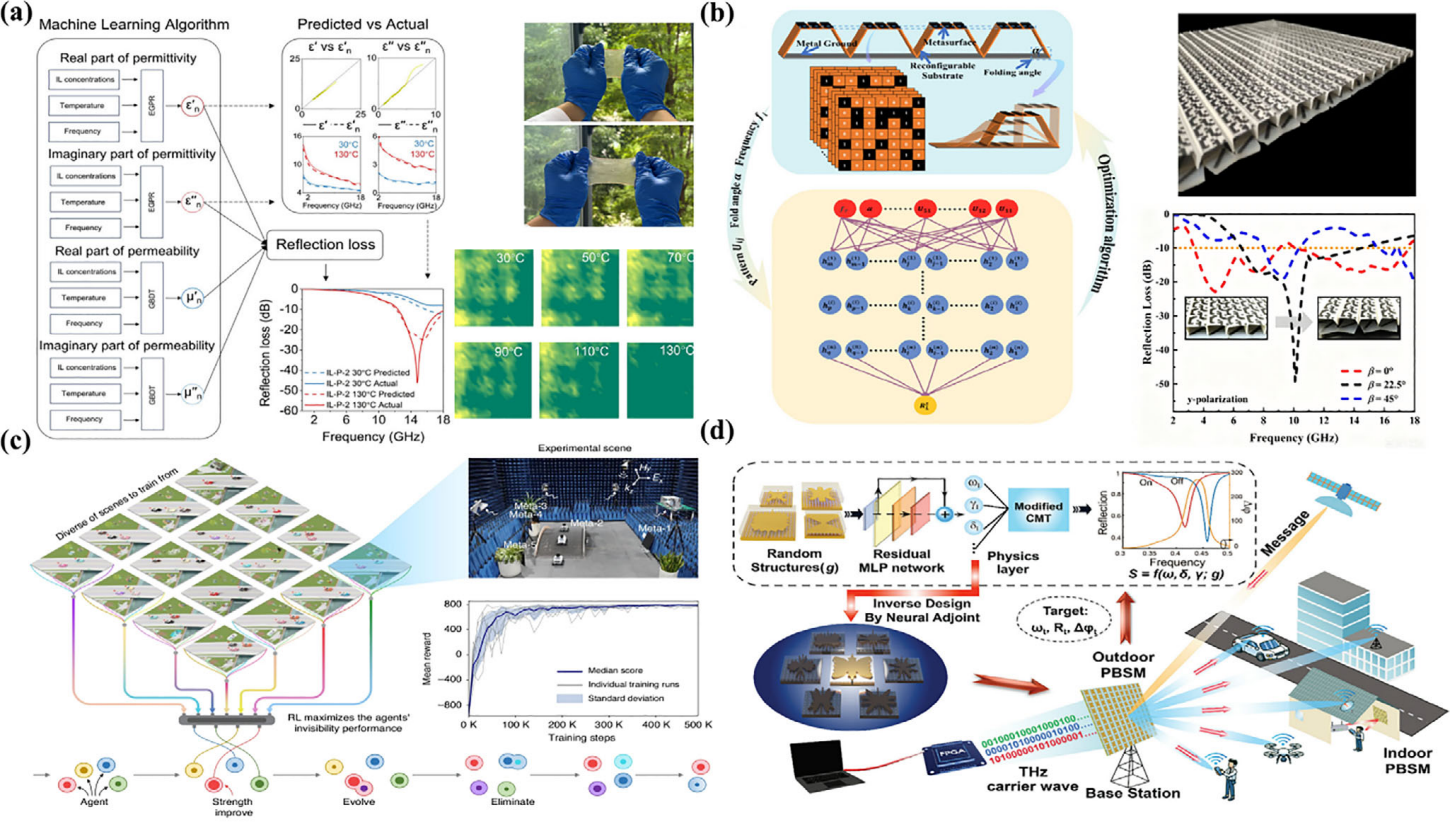
Data‐driven intelligent metasurface. (a) Ionic Liquid Polymer Metasurface with Neural Network‐Driven Microwave Modulation. Reprinted under the terms and conditions of the Creative Commons Attribution (CC BY) license (https://creativecommons.org/licenses/by/4.0/) [159]. Copyright 2026 the authors. Published by Springer Nature. (b) Deep Learning Inverse Design of Origami‐Kirigami MXene Metasurface for Reconfigurable Microwave Absorption. Reprinted with permission [160]. Copyright 2024 American Chemical Society. (c) MetaSeeker: Model‐based reinforcement learning framework integrating recognition, representation, dynamics, and prediction networks for autonomous decision‐making and adaptive optimization of distributed metasurfaces. Reprinted with permission [162]. Copyright 2025 Springer Nature. (d) Physics‐Informed Inverse Designed Liquid Crystal Metasurface for Multi‐Bit Terahertz Beam Steering. Reprinted under the terms and conditions of the Creative Commons Attribution (CC BY) license (https://creativecommons.org/licenses/by/4.0/) [164]. Copyright 2024 the authors. Published by Wiley‐VCH.

As a core branch of machine learning, neural networks possess powerful nonlinear mapping and high‐dimensional data processing capacities, and have become a widely used framework for data‐driven metasurface engineering. In intelligent stealth metasurface research, neural networks undertake two core tasks: serving as high‐speed forward surrogate models to rapidly predict EM responses of given metasurface layouts, and acting as inverse designers to directly output optimal structural parameters matching target EM performance. Both schemes effectively bypass the core drawbacks of traditional design pipelines, that is, massive computational cost, long cycles, and over‐reliance on domain expertise. For forward surrogate modeling, well‐trained neural networks establish explicit mappings linking structural geometry, material properties, and EM characteristics; training datasets are generally generated from large‐scale full‐wave simulations. After full convergence, such surrogate models deliver precise performance predictions at millisecond‐level speed, far outperforming traditional numerical solvers. Niu et al. [158] proposed a machine‐learning‐assisted design strategy combined with droplet printing for flexible graphene terahertz absorbing metamaterials. By building forward surrogate models that correlate critical geometric dimensions with effective absorption bandwidth, the framework enables fast performance evaluation and structural optimization. Given the characteristic sizes of coupled split‐ring resonators, the model accurately predicts spectral regions with absorptivity over 90%, accelerating the screening of optimal unit configurations. Guided by the trained network, the final structure is obtained via only a handful of iterative trials and fabricated by aerosol jet printing. The flexible absorber achieves average absorptivity above 90% across 1.16–1.63 THz at an ultrathin thickness of roughly 45 µm, while maintaining excellent mechanical flexibility and long‐term environmental stability. This integrated data‐enabled design‐to‐manufacturing workflow outperforms conventional trial‐and‐error schemes in both efficiency and prediction accuracy. Dong et al. [159] constructed a hybrid machine learning framework combining Gaussian process regression and gradient boosting decision trees to tailor microwave‐tunable surfaces based on ionic liquid polymers, as illustrated in Figure 16a. The forward model quantifies the relationships among component concentration, ambient temperature, and intrinsic dielectric constants, realizing fast prediction and programmable tuning of EM parameters. Using only ionic liquid proportion and temperature as input variables, the model precisely recovers complex permittivity and permeability spectra, further supporting inverse formulation design for customized microwave modulation. Materials optimized via this method are fabricated by solution casting, achieving continuous permittivity tuning from 0.2 to 0.6 within 8–12 GHz with stable performance over 1000 thermal cycles. For inverse design, neural networks directly build inverse mappings from target EM responses to feasible structural geometries, eliminating redundant forward iteration loops required by traditional optimization algorithms. A mainstream implementation adopts paired neural network architectures, which take target spectral responses as input and output corresponding meta‐atom geometric parameters. Li et al. [160] realized fast inverse design of origami‐based absorbing structures using artificial neural networks, as shown in Figure 16b. Encoded layout patterns and folding angles are imported to predict absorption spectra within milliseconds; the trained network is embedded into genetic algorithms to retrieve optimal geometries satisfying designated absorption criteria in minutes. Origami metasurfaces screen‐printed with low‐concentration MXene‐PEDOT:PSS ink realize efficient microwave absorption in the X‐band and Ku‐band under unfolded and folded states, respectively. This intelligent design combined with additive manufacturing provides a scalable route for on‐demand customization and rapid prototyping of reconfigurable metamaterials.

These advantages notwithstanding, neural‐network‐based inverse design retains several inherent limitations that become pronounced for cross‐scale multispectral stealth metasurfaces. A fundamental issue is the one‐to‐many mapping ambiguity: a given target spectrum admits countless realizations across atomic dopants, microscale morphologies, and macroscale lattice patterns. Networks trained with mean‐square‐error loss tend to collapse these possibilities into an averaged compromise; absent explicit manufacturability constraints, the predicted geometry may prove geometrically invalid or violate fabrication limits. A further practical constraint is data scarcity. High‐fidelity multiphysics simulations for multispectral stealth metasurfaces incur substantial computational cost, while experimental datasets remain sparse and imbalanced. Prediction accuracy degrades markedly when operating conditions—incident angle, temperature, material loss—deviate from the training distribution. Moreover, the idealized boundary conditions assumed in simulation engender a systematic gap between predicted and measured performance. Finally, the black‐box character of neural networks constrains engineering confidence: the model establishes input‐output mappings without elucidating how atomic losses, interface effects, and lattice geometry collectively govern RCS suppression, precluding the extraction of transferable cross‐scale design principles and complicating failure diagnosis.

##### 3.3.3.2 Reinforcement Learning Enabled Dynamic Control and Adaptive Stealth of Metasurfaces

Reinforcement learning (RL) is a powerful machine learning paradigm where intelligent agents maximize cumulative reward values through continuous environmental interaction and trial‐and‐error exploration, providing solid theoretical frameworks and practical solutions to dynamic stealth problems. For metasurface dynamic regulation and adaptive camouflage, RL agents first acquire real‐time EM environmental states, including incident wave parameters and scattered field data collected by integrated sensors. The agents then execute corresponding control actions, such as adjusting meta‐unit bias voltages or switching phase states of phase‐change materials, and subsequently receive quantitative reward signals that quantify stealth performance, such as RCS reduction, signal optimization degree, and matching level with ideal stealth signatures. Through sufficient interactive iteration and policy updating, RL agents derive optimal control policies suitable for complex dynamic scenes, enabling real‐time self‐adaptive tuning of metasurface EM responses. Tan et al. [161] proposed deep reinforcement learning empowered intelligent terahertz beamforming. The core innovation introduces RL frameworks for on‐demand phase distribution prediction. Taking time‐varying target terminal positions as input, the system instantly generates optimal metasurface phase configurations. Loss functions are defined based on the deviation between predicted and target radiation patterns, with automatic differentiation supporting online network parameter updating and millisecond‐scale adaptive beam steering. This scheme fundamentally overcomes the slow response bottleneck of traditional numerical optimization, and experimental tests on silicon metasurfaces verify its capacity to precisely steer EM beams toward multiple dynamically moving terminals. A landmark contribution is the MetaSeeker platform proposed by Chen's research group [162] a clustered metasurface system driven by self‐play reinforcement learning, designed to construct continuous EM stealth regions under time‐varying surrounding environments. The system adopts a dual‐layer optimization architecture integrating deep reinforcement learning and swarm optimization algorithms, achieving autonomous coordinated regulation of distributed metasurface arrays with a minimum response latency of 89 ms and stealth fidelity exceeding 99.5%, as shown in Figure 16c. This pioneering work upgrades intelligent stealth technology from single‐device intelligence to spatial collective intelligence, demonstrating great application potential of RL for complex distributed dynamic stealth architectures. Coordinated operation of randomly distributed reconfigurable metasurface clusters forms continuous reconfigurable invisible EM domains without full target encapsulation, allowing arbitrary moving objects inside to maintain stable stealth performance. The core swarm RL‐based MetaSeeker framework contains four functional sub‐networks for environment recognition, feature extraction, dynamic state analysis, and future trend prediction. Combined with Monte Carlo Tree Search (MCTS) and Population Based Training (PBT), it builds virtual environment mappings consistent with real physical fields to support autonomous agent evolution and in situ environmental adaptation. Furthermore, the SHAP interpretability algorithm is embedded to reveal the inherent decision logic of intelligent controllers. Experimental results confirm the system achieves an environmental similarity ratio above 99.5% across 4.5–6.5 GHz under multi‐angle detection, realizing broadband stealth over a 1.5 GHz bandwidth and omnidirectional camouflage. Comprehensive dynamic functional tests, including target size scaling, high‐speed motion tracking, and scene switching, have been successfully conducted. This research breaks the technical limitations of traditional stealth strategies, driving metasurface applications from single‐structure modulation to cluster cooperative manipulation. It also establishes an innovative paradigm for remote intelligent control of complex EM spaces, offering design references for wireless communication and non‐line‐of‐sight imaging systems.

Reinforcement learning confers real‐time adaptability upon stealth metasurfaces, yet its practical deployment entails several unresolved difficulties. A primary challenge is reward signal conflict. Microwave absorption and infrared suppression are physically antagonistic; maximizing one typically compromises the other. A single weighted reward function cannot reconcile this tension, and consensus metrics for dynamic multispectral backgrounds remain lacking. A second issue is temporal latency. Non‐negligible delay separates the agent's decision from the actual response of the actuation hardware—PIN diodes or phase‐change media. Under rapidly evolving threat environments, this latency manifests as transient gaps in camouflage coverage. A third concern is interpretability. Conventional RL policies are often difficult to interpret: the physical rationale underlying a given control sequence is rarely traceable, which raises reliability concerns for safety‐critical deployments.

##### 3.3.3.3 Physics‐Informed Intelligent Design Methodology

Pure data‐driven machine learning models such as neural networks and generative adversarial networks (GANs) have achieved remarkable progress in metasurface design. Nevertheless, such methods demand massive simulated datasets for adequate training, and their intrinsic black‐box characteristic leads to poor physical interpretability and limited generalization performance. To resolve these drawbacks, physics‐informed intelligent design strategies have been proposed. Their core principle explicitly embeds well‐established physical laws, prior domain knowledge, and fundamental physical constraints into the construction and training pipelines of machine learning models, including Maxwell's equations, energy conservation, and definite boundary conditions. Such methodologies show broad application prospects in advanced metasurface engineering. Bao et al. [163] built a physics‐augmented deep learning framework integrating extended macroscopic physical models and residual networks. Targeting programmable digital coding metasurfaces, this framework constructs quantitative mappings between target beam directions and digital coding sequences, rapidly generating optimal coding layouts for single‐beam and dual‐beam radiation. Constrained by rigorous physical priors, the model achieves satisfactory convergence using only a small volume of labeled simulation data and outputs physically consistent coding configurations within milliseconds. The resulting designs feature lower sidelobe levels than those obtained via traditional optimization, and experimental measurements verify the optimized coding patterns realize precise directional beam emission as designed. Xu et al. [164] embedded modified coupled‐mode theory (MCMT) as a physical constraint layer into residual multilayer perceptrons, realizing high‐efficiency inverse metasurface design with only three input variables: target phase shift, reflection amplitude, and operating frequency, as illustrated in Figure 16d. This hybrid framework simultaneously improves design accuracy and model interpretability, achieving an ultra‐wide phase tuning range up to 300° at 465 GHz. The fabricated liquid‐crystal programmable metasurfaces support 1‐bit, 2‐bit, and three‐state coding layouts, with a maximum beam deflection angle of 68° and greatly expanded angular coverage. This work provides a simple, efficient route for customized reconfigurable EM devices, with promising applications in 6G wireless communications, hyperspectral imaging, and holographic displays.

Physics‐informed methods alleviate certain data and interpretability limitations, yet introduce trade‐offs intrinsic to the framework. Most implementations employ simplified Maxwell equations that omit higher‐order coupling effects such as multipole interactions. This approximation preserves tractability but constrains the discovery of unconventional meta‐atom topologies. For multispectral designs spanning radar and infrared bands, embedding the complete set of coupled partial differential equations incurs prohibitive memory overhead and impedes convergence, forcing an irreducible trade‐off between accuracy and computational affordability. More critically, current physical loss terms constrain only the macroscopic EM response, leaving atomic‐scale doping levels and microscale filler morphology unencoded. The inverse‐designed geometry therefore still requires post‐hoc manual material selection, undermining the objective of fully automated end‐to‐end design. Finally, many emerging physical phenomena and novel functional materials lack mature analytical descriptions, which limits the universal deployment of fully physics‐constrained stealth design schemes.

Combined with the above exclusive drawbacks of three branches of data‐driven algorithms, we summarize five universal fundamental limitations shared by current AI frameworks, which fundamentally hinder large‐scale deployment of multispectral stealth metasurfaces. First, high‐quality multi‐physics coupled datasets are extremely costly to generate and suffer severe sample imbalance. Second, many models are trained primarily on idealized simulation data, resulting in widespread performance degradation after physical fabrication. Third, deep learning‐based methods are inherently black‐box systems lacking quantitative cross‐scale physical interpretability. Fourth, existing architectures cannot fully embed coupled EM and thermal radiation constraints, and may output configurations that violate fundamental physical laws and fail to balance multi‐band camouflage performance. Fifth, predefined meta‐atom topological templates restrict innovative structural exploration, while training large‐scale models requires massive GPU resources and high power consumption. To systematically resolve these universal bottlenecks, an integrated intelligent design framework combining physics‐informed neural networks, multi‐fidelity transfer learning, uncertainty quantification, and closed‐loop simulation‐fabrication‐characterization iteration should be constructed to remedy deficiencies in dataset quality, physical constraints, interpretability and fabrication robustness, representing an indispensable pathway toward mass‐producible, adaptive next‐generation intelligent stealth metamaterials.

## 4 Conclusions and Outlook

### 4.1 Conclusions

Stealth technology has shifted its core design logic. Early schemes rely on modifying bulk materials, while modern approaches center on structure‐driven design. Metasurfaces are two‐dimensional artificial microstructures capable of precise subwavelength EM control. They break multiple inherent physical limits of traditional stealth materials in bandwidth, thickness, weight and dynamic tuning capacity. Three core modulation mechanisms jointly create their competitive strengths. Wavefront shaping offers flexible control over EM propagation paths. Local resonance strengthens energy capture and near‐field loss. Spatial dispersion widens effective working bandwidth. These mechanisms jointly solve classic drawbacks of lossy materials, including the thickness‐bandwidth tradeoff and Snoek restriction, as well as static‐response limitations. Various tunable routes relying on electric, magnetic, optical, thermal or mechanical stimuli can dynamically adjust stealth frequency and scattering signatures. Advanced design ideas such as multi‐layer meta‐units, programmable coding and AI inverse design further expand broadband performance and optimize multi‐mode wave control. Current metasurface research covers radar, infrared, visible and acoustic bands, forming diverse absorbing and reflection‐modulating stealth schemes. These designs provide new solutions for military camouflage, EM compatibility and thermal management. In practical performance, metasurfaces balance ultrathin thickness, lightweight form factor, dynamic tuning and multispectral compatibility. Their absorption bandwidth, adjustable reflection loss and environmental stability outperform traditional materials, supporting the transition toward adaptive and programmable EM control.

### 4.2 Outlook

- *Embodied intelligence for conformal integration and self‐adaptive stealth systems*: Combining embodied intelligence with metasurface design transforms traditional passive coatings into integrated dynamic platforms that support sensing, analysis and active regulation. Such structures can fit complex curved surfaces of robots and special equipment, realizing coordinated structural and stealth functions to avoid defects of separate static camouflage layers. Metasurfaces act as intelligent skins tightly attached to carrier surfaces, embedded with miniature EM sensors, infrared detectors and flexible actuators. Sensors deployed across the surface collect incident wave spectra, background thermal radiation and equipment posture data during covert tasks. Intelligent algorithms then generate targeted adjustment strategies. The system can switch between low‐emissivity forest camouflage and broadband microwave absorption for urban environments, and adjust equipment posture together with EM responses to lower exposure risks. For grouped equipment deployment, distributed communication synchronizes the phase of separated metasurface units. The overall radar echo signal converges to the level of small single targets and forms omnidirectional stealth shielding. Flexible polymer and stretchable conductive meta‐units adapt to robot joints and curved components. For example, serpentine metal arrays maintain reflection loss fluctuation within 1 dB during repeated bending. Modular layout allows fast function reconfiguration to adapt to various mission scenarios.
- *Scenario‐oriented multifunctional integration and performance synergy*: Future metasurfaces will no longer only realize single stealth effects. They can serve integrated platforms that deliver stealth, wireless communication, environmental sensing and thermal management, following the principle that structure determines multi‐scenario functions. For military equipment, integrated metasurfaces achieve multispectral camouflage, anti‐interference communication and battlefield signal detection. Devices stay low‐observable in covert states and switch to high‐transmission mode for real‐time data transmission when communication is needed, while built‐in sensors monitor surrounding radar signals. In aerospace skin applications, metasurfaces combine wideband microwave absorption and passive cooling to cut radar cross‐section and suppress infrared radiation. For wearable devices, the material balances shielding performance and wearing comfort, and can be matched with physiological monitoring modules to expand application value. Conformal structural design is the core way to realize multi‐function coordination. Optimized single‐layer meta‐layouts support multiple physical modulation effects at the same time. Typical designs use multi‐mode resonance to realize microwave absorption and biological signal testing, or finely tuned micro‐nano structures to balance visible camouflage and infrared emissivity control.
- *Bionics‐driven cross‐domain innovation and performance enhancement*: Bionic thinking becomes an important driving force for metasurface innovation. Structures and regulation modes evolved in natural creatures offer references for more efficient, environment‐adaptive stealth materials. In structural design, butterfly wing hierarchical gratings coordinate structural color and infrared radiation control. Beetle shell multi‐layer laminates create lightweight high‐strength composite substrates. Porous textures inspired by desert plants build multi‐function metasurfaces with moisture control, radiative cooling and wave absorption, which work stably under harsh conditions. For dynamic response, cuttlefish multi‐layer pigment and muscle systems inspire microfluidic electrochromic metasurfaces. Pigment migration and electro‐optic switching together realize continuous visible‐infrared spectrum matching. Chameleon skin deformation principles guide stretchable meta‐arrays that adjust camouflage patterns and multi‐band EM responses under mechanical force. Bamboo fiber hollow foam structures support lightweight broadband absorption and improved impact resistance. Dolphin skin viscoelastic layers are adopted for underwater devices to achieve simultaneous drag reduction, sound absorption and tunable EM scattering.
- *Standardization and low‐cost manufacturing for engineering implementation*: Large‐scale application of metasurfaces requires breakthroughs in mass production, long‐term stability and unified industrial standards, to transfer laboratory prototypes into practical engineering products. Manufacturing processes need low‐cost methods compatible with existing industrial lines, including AI optimized roll‐to‐roll printing, laser direct writing and composite 3D printing. Machine vision monitors machining errors in real time. Intelligent compensation algorithms adjust unit geometry automatically to keep consistent structure and reduce waste. Bionic template replication can cut the cost of flexible stealth skins to a fraction of traditional precision microfabrication and support mass production. Accelerated aging test data support AI performance decay models. These models predict service life and performance loss under high temperature, high humidity, salt spray and continuous vibration. Prediction results guide anti‐corrosion coating and interfacial reinforcement to improve long‐term stability. Uniform industrial standards can be built via big data and AI analysis. The standards cover meta‐unit dimensional tolerance, EM test specifications and unified mechanical‐electrical interfaces. Complete basic module systems support interchange between different manufacturers and accelerate industrialization for national defense and civilian EM compatibility equipment.

## Author Contributions

**Shuhao Wang**: writing – review and editing, investigation, writing – original draft. **Xing Yang**: funding acquisition, supervision, visualization, project administration. **Liping Liu**: investigation, methodology, supervision, writing – review and editing, writing – original draft. **Yang Peng**: investigation, validation, formal analysis. **Yangyang Wang**: funding acquisition, project administration. **Hualiang Lv**: writing – original draft, supervision, validation, visualization, methodology.

## Conflicts of Interest

The authors declare no conflicts of interest.

## Data Availability

The data that support the findings of this study are available on request from the corresponding author. The data are not publicly available due to privacy or ethical restrictions.
